# Glycoprotein-induced phase separation drives unconventional secretion of galectin-3

**DOI:** 10.1038/s41467-026-76321-w

**Published:** 2026-08-06

**Authors:** Zihan Zhao, Zhen He, Hongming Gu, Andong Zhou, Yibing Wang, Zhaoyi Liang, Jie Geng, Xuejiao Xu, Menghui Wang, Chunyao Li, Yuying Fan, Hairong Cheng, Lin Sun, Kevin H. Mayo, Avraham Raz, Guihua Tai, Yifa Zhou

**Affiliations:** 1 Engineering Research Center of Glycoconjugates Ministry of Education, Jilin Provincial Key Laboratory of Chemistry and Biology of Changbai Mountain Natural Drugs, School of Life Sciences, Northeast Normal University, Changchun, China; 2 Department of Biochemistry, Molecular Biology and Biophysics, University of Minnesota, Minneapolis, MN USA; 3 Department of Oncology, School of Medicine, Karmanos Cancer Institute, Wayne State University, Detroit, MI USA

**Keywords:** Protein transport, Glycobiology, Endosomes

## Abstract

The mechanism of unconventional protein secretion remains an unresolved issue. Here, we describe an unconventional protein secretion pathway for galectin-3 that is mediated by phase separation and condensation. Using four lysosomal damage models, we observed a rapid, pronounced release of galectin-3 in large, non-exosomal particles. This secretion is driven by glycoprotein-induced galectin-3 phase separation and is independent of pyroptosis and secretory autophagy. During phase separation, the S-face of galectin-3 carbohydrate recognition domain binds glycoproteins that triggers galectin-3 N-terminal tail release and condensation. These condensates then recruit ALG-2 via the exposed N-terminal tail. ALG-2 directs the condensates to the endoplasmic reticulum–late endosome interface. After translocation into late endosomes, galectin-3 condensates are secreted into the extracellular milieu by SNARE-dependent vesicular transport. This mechanism of exporting phase-separated protein condensates may serve as a clean-up response to membrane damage.

## Abstract (web summary)

Unconventional protein secretion enables the extracellular release of proteins lacking canonical endoplasmic reticulum and Golgi targeting signals. Here, the authors describe an unconventional secretory pathway for galectin-3 that is mediated by phase separation and condensation, revealing a cellular strategy for maintaining homeostasis under lysosomal stress.

## Introduction

Protein secretion is a fundamental cellular process that enables the release of bioactive molecules, thereby regulating intercellular communication and facilitating adaptation to physiological or pathological challenges^1–3^. While conventional protein secretion relies on signal peptide-directed trafficking through the endoplasmic reticulum (ER) and Golgi apparatus, a significant number of proteins lacking these canonical signals utilize alternative pathways collectively termed unconventional protein secretion (UcPS)^4–6^. UcPS is an enigmatic and complex phenomenon that has garnered increasing research attention. In recent years, several UcPS routes have been proposed^7–16^, including extracellular vesicle-mediated release, pyroptosis-mediated pore extrusion, transmembrane emp24 domain-containing protein 10 (TMED10)-mediated transmembrane transport, and ubiquitin-specific protease 19 (USP19)-mediated misfolding-associated protein secretion (MAPS). These pathways mediate the extracellular delivery of cytokines, alarmins, chaperones, and MAPS, which are essential for immune surveillance, stress adaptation, and disease pathogenesis^9–11,13^. Nevertheless, the mechanisms underlying UcPS remain poorly understood. The diversity of pathways and molecular triggers under various physiological and pathological conditions still requires extensive investigation.

Galectin-3 (Gal-3), a prototypical UcPS protein, features a long collagen-like N-terminal tail (NT) and a C-terminal carbohydrate recognition domain (CRD)^17^. Like all galectins, the Gal-3 CRD is composed of a six-stranded β-sheet folded against a five-stranded β-sheet, forming a β-barrel tertiary structure. The S-face β-sheet (six strands) binds glycans, while the NT transiently interacts with the opposing F-face β-sheet (five strands)^18^. Upon binding to multivalent glycans, Gal-3 oligomerizes via intermolecular interactions between NTs and between NTs and CRDs^18,19^. Gal-3 has emerged as a key mediator in inflammation, cancer metastasis, and fibrosis^20–23^. Elevated Gal-3 levels have been detected in the serum of patients with various diseases^24–29^. Despite these findings, the mechanisms and triggers underlying Gal-3 secretion in different disease states remain poorly defined.

Furthermore, elevated serum Gal-3 levels often coincide with lysosomal damage-associated pathological conditions, including Alzheimer’s disease (AD), pulmonary fibrosis, SARS-CoV-2 infection, and fungal infections^30–34^. Additionally, Gal-3 rapidly accumulates at sites of lysosomal damage^35,36^, raising the possibility that lysosomal membrane rupture may be associated with Gal-3 secretion.

Recent studies have shown that Gal-3 undergoes liquid-liquid phase separation (LLPS) upon binding to lipopolysaccharides (LPS) and glycoproteins^37,38^. Gal-3 LLPS is a dynamic oligomerization/condensation process in which Gal-3 molecules are rapidly exchanged between condensates and the surrounding solution^38^. LLPS has emerged as a central mechanism in diverse cellular processes and diseases^39–41^. Notably, lysosomal damage can induce Gal-3 phase separation, resulting in the formation of macromolecular condensates that may themselves contribute to cellular stress^38^. This raises an important question: do such phase-separated condensates participate in Gal-3 secretion? The role of LLPS in UcPS remains unexplored in the current literature.

Here, we report a LLPS-driven UcPS pathway for Gal-3. In this mechanism, glycoproteins exposed on damaged lysosomal membranes induce Gal-3 phase separation. The resulting condensates then recruit the effector molecule ALG-2 (apoptosis-linked gene 2 protein homolog), which mediates Gal-3 secretion by vesicular transport.

## Results

### Lysosomal damage induces Gal-3 unconventional secretion

**Fig. 1:**
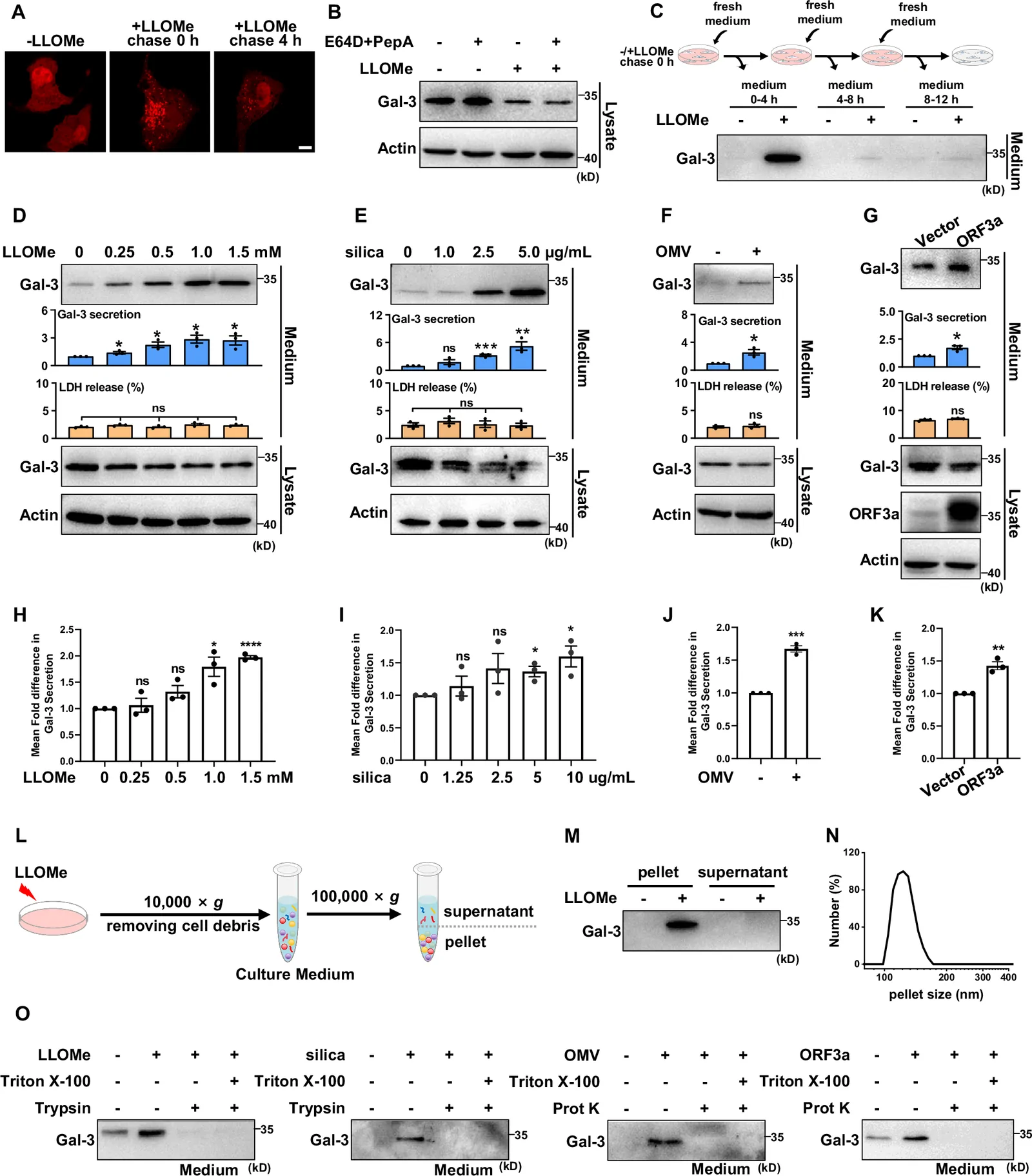
Lysosomal damage induces Gal-3 secretion*.* **A** Representative images of Gal-3 puncta in HeLa cells transfected with the mCherry-Gal-3 plasmid. Cells were treated with 1 mM LLOMe for 30 min, followed by a 4 h incubation (chase) in serum-free medium (SFM) after LLOMe washout. Scale bar, 10 µm. **B** Intracellular Gal-3 levels in HeLa cells. Cells pretreated with or without E64D and pepstatin A (Pep A) were treated with LLOMe as in (**A**). Cell lysates were analyzed by Western blotting. **C** Time-dependent secretion of Gal-3 in LLOMe-treated HeLa cells. Cells were treated with 1 mM LLOMe for 30 min, followed by a 12 h incubation in SFM after LLOMe washout. Culture medium was collected and analyzed by Western blotting in 0–4 h, 4–8 h, and 8–12 h intervals. **D**–**G** Western blotting of Gal-3 secretion in various lysosomal damage models. HeLa cells were treated with increasing concentrations of LLOMe for 30 min (**D**), silica particles for 30 min (**E**), outer membrane vesicles (OMV) for 2 h (**F**), or transfected with pCMV3-C-ORF3a-FLAG plasmid for 24 h (**G**). After treatment, cells were rinsed and incubated in SFM for 4 h, and the medium and cell lysates were analyzed. Bars indicate Gal-3 secretion and LDH release. *n* = 3 independent experiments. **H**–**K** ELISA-based quantification of Gal-3 secretion in the lysosomal damage models described in (**D**–**G**). Data represent *n* = 3 independent experiments. **L** Schematic diagram showing separation of the 100,000 × *g* pellet by ultracentrifugation. **M** Western blotting of Gal-3 in the 100,000 × *g* pellet and supernatant. **N** Particle size distribution in the 100,000 × *g* pellet analyzed by dynamic light scattering (DLS). **O** Protease sensitivity of secreted Gal-3. Cells were treated with LLOMe, silica, OMV, or ORF3a as described in (**D**–**G**). Conditioned medium was digested with trypsin or proteinase K in the presence or absence of Triton X-100. Data are shown as the mean ± SEM. *p < 0.05, **p < 0.01, ***p < 0.001, and ****p < 0.0001, two-tailed Student’s *t* test. ns, not significant. Source data are provided as a Source Data file.

Although previous studies have shown that Gal-3 is recruited to sites of endomembrane damage^35^, the fate of these Gal-3 molecules remains unclear. Using an L-leucyl-L-leucine methyl ester (LLOMe)-induced lysosomal damage model^42^ (Supplementary Fig. 1A, F), we observed the formation of Gal-3 puncta during LLOMe treatment (chase 0 h), which gradually disappeared over time (chase 4 h; Fig. 1A), accompanied by a reduction in intracellular Gal-3 levels (Fig. 1B).

To determine whether these Gal-3 puncta were trafficked to and degraded by lysosomes, we treated cells with E64D and pepstatin A, inhibitors of lysosomal proteases. These inhibitors failed to prevent Gal-3 reduction (Fig. 1B), suggesting that the decrease in intracellular Gal-3 is not due to lysosomal degradation. Notably, we detected a rapid and significant increase in extracellular Gal-3 within the first 4 h following LLOMe treatment (Fig. 1C). This observation was confirmed across multiple cell lines (HeLa, RAW264.7, A549, HT-22, and Hepa 1–6) by both Western blotting (Fig. 1D and Supplementary Fig. 2A–D) and ELISA (Fig. 1H and Supplementary Fig. 2F). Furthermore, lysosomal damage accompanied by Gal-3 secretion was also observed in cells overexpressing SARS-CoV-2 ORF3a (mimicking viral infection), cells treated with bacterial outer membrane vesicles (OMVs, mimicking bacterial infection), and cells exposed to silica (mimicking silicosis) (Fig. 1E–G, I–K and Supplementary Figs. 1B–E, G, and 2E, G). These findings indicate that lysosomal damage consistently triggers Gal-3 secretion. Such secretion is not attributable to plasma membrane damage or increased cell death because neither a significant increase in LDH release (Fig. 1D–G and Supplementary Fig. 2A–E) nor a decrease in cell viability (Supplementary Fig. 3) was observed in these models, compared to normal cells.

To further characterize the secreted Gal-3, we subjected culture medium from LLOMe-treated cells to ultracentrifugation at 100,000 × *g*, separating it into pellet and supernatant fractions (Fig. 1L). Nearly all Gal-3 was recovered in the pellet as particles approximately 120 nm in diameter (Fig. 1M, N). To determine whether these Gal-3 particles were membrane-enclosed, we treated the medium with trypsin or proteinase K in the presence or absence of Triton X-100, a membrane-disrupting agent. In both cases, Gal-3 was degraded (Fig. 1O), indicating that the particles lack membrane encapsulation.

We then investigated whether this lysosomal damage-induced Gal-3 secretion occurred via previously proposed mechanisms. First, Gal-3 is not secreted via exosomes because secreted Gal-3 was not membrane-enclosed, as demonstrated above (Fig. 1O). Second, Gal-3 secretion is independent of pyroptosis. Although the pyroptosis-related protein gasdermin D (GSDMD) has been previously implicated in the secretion of Gal-3 and Galectin-1 (Gal-1)^43–45^, our study shows that lysosomal damage activates gasdermin E (GSDME), but not GSDMD, as evidenced by an increase in the active N-terminal fragment GSDME-N (Supplementary Fig. 4A). However, this activation is not required for Gal-3 secretion because: (a) treatment with Z-VAD-FMK, a pan-caspase inhibitor that blocks GSDME activation, does not affect Gal-3 secretion (Supplementary Fig. 4B), and (b) knockdown of GSDME expression does not suppress Gal-3 secretion (Supplementary Fig. 4C). Third, Gal-3 secretion does not occur via secretory autophagy^46^. Neither pharmacological inhibition of autophagy (using 3-methyladenine [3-MA] and DC-LC3in-D5) nor knockdown of autophagy-related proteins (TRIM16, Beclin1, ULK1, and ATG16L1) reduces Gal-3 secretion (Supplementary Fig. 5). On the contrary, inhibition by 3-MA or DC-LC3in-D5, as well as knockdown of TRIM16 and ATG16L1, enhances Gal-3 secretion.

Together, these data demonstrate that lysosomal damage triggers an unconventional mechanism of Gal-3 secretion.

### Gal-3 secretion depends on *N*-glycosylated lysosomal proteins

**Fig. 2:**
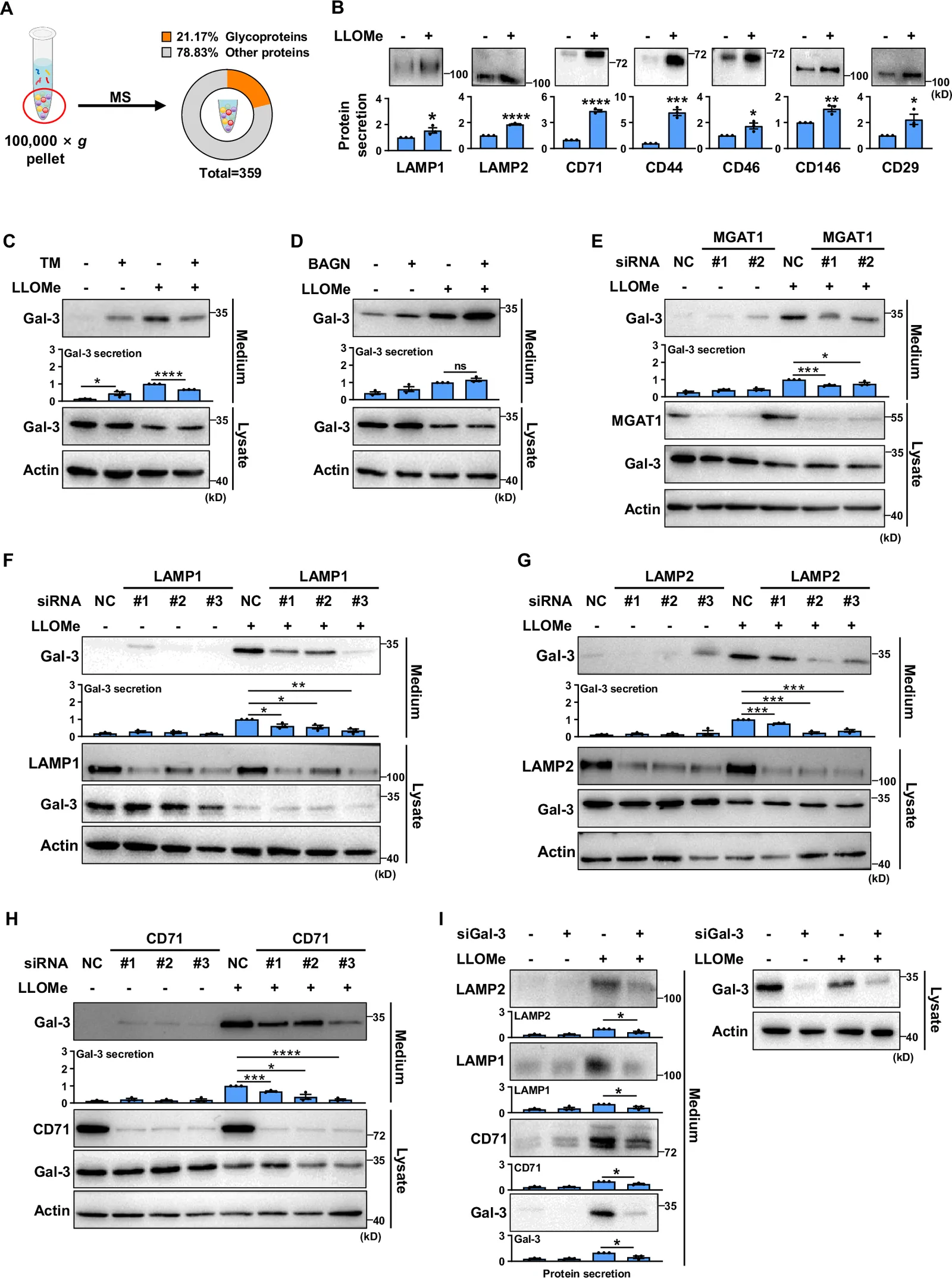
Glycoproteins mediate Gal-3 secretion. **A**, **B** Analysis of glycoprotein secretion. HeLa cells were treated with 1 mM LLOMe for 30 min, followed by a 4 h incubation in SFM after LLOMe washout. The 100,000  × *g* pellet was isolated from the medium and analyzed by mass spectrometry (MS) (**A**) and Western blotting (**B**). *n* = 3 independent experiments. **C**, **D** Effects of glycosylation inhibitors on Gal-3 secretion. HeLa cells pretreated with tunicamycin (TM) (**C**) or benzyl 2-acetamido-2-deoxy-α-D-galactopyranoside (BAGN) (**D**) were treated with 1 mM LLOMe for 30 min, followed by a 4 h incubation in SFM. The medium and cell lysates were analyzed by Western blotting. *n* = 3 independent experiments. **E**–**I** Effects of gene knockdown on the secretion of Gal-3 and glycoproteins. HeLa cells were transfected with siRNAs targeting MGAT1 (**E**), LAMP1 (**F**), LAMP2 (**G**), CD71 (**H**), or Gal-3 (**I**). Cells were then treated with 1 mM LLOMe for 30 min, followed by a 4 h incubation in SFM. For each condition, the medium was analyzed by Western blotting for secreted Gal-3 or glycoproteins, and cell lysates were probed to assess knockdown efficiency and Gal-3 level. *n* = 3 independent experiments. Bars indicate protein secretion. Data are shown as the mean ± SEM. *p < 0.05, **p < 0.01, ***p < 0.001, and ****p < 0.0001, two-tailed Student’s *t* test. ns, not significant. Source data are provided as a Source Data file.

We next asked whether other proteins are co-released with Gal-3 during lysosomal damage. Mass spectrometry (MS) (Fig. 2A and Supplementary Data 1) revealed that, in addition to Gal-3, many glycoproteins are released, constituting approximately 21% of the total proteins identified in the 100,000  × *g* pellet fraction—an observation further supported by Western blotting (Fig. 2B). This finding is notable, because glycoproteins are physiological ligands of Gal-3. In normal cells, lysosomal glycoproteins reside on the luminal side of lysosomes but are exposed to the cytoplasm (a reservoir for Gal-3) when the lysosomes are damaged. Moreover, some lysosomal glycoproteins significantly affected in our proteomic analyses (e.g., lysosome-associated membrane protein (LAMP)1, LAMP2, and CD71) have been shown to mediate recruitment of Gal-3 to damaged lysosomes and function in lysosomal repair^35,36^.

The co-release of Gal-3 and these lysosomal glycoproteins suggests a role for glycoproteins in Gal-3 secretion. To explore this, we inhibited glycosylation pathways using tunicamycin (TM) to block *N*-glycosylation and benzyl 2-acetamido-2-deoxy-α-D-galactopyranoside (BAGN) to inhibit *O*-glycosylation^47–49^. As shown in Fig. 2C, D, Gal-3 secretion was significantly reduced by TM but not by BAGN, indicating that *N*-glycosylation is essential for this process. To investigate this further, we knocked down mannoside acetylglucosaminyltransferase 1 (MGAT1), a key enzyme required for the biosynthesis of hybrid and complex *N*-glycans^50^. As shown in Fig. 2E, MGAT1 knockdown markedly reduced Gal-3 secretion.

We then assessed the contribution of specific glycoproteins by focusing on LAMP1, LAMP2, and CD71. Knockdown of any of these proteins led to a significant decrease in Gal-3 secretion (Fig. 2F–H), suggesting that multiple *N*-glycosylated proteins, rather than a single effector, contribute to this secretory process. Notably, knocking down Gal-3 also reduced the secretion of LAMP1, LAMP2, and CD71 (Fig. 2I), indicating a reciprocal relationship. Together, these results suggest that glycoprotein and Gal-3 secretion are interdependent during lysosomal damage.

### Glycoprotein-induced phase separation drives Gal-3 secretion

**Fig. 3:**
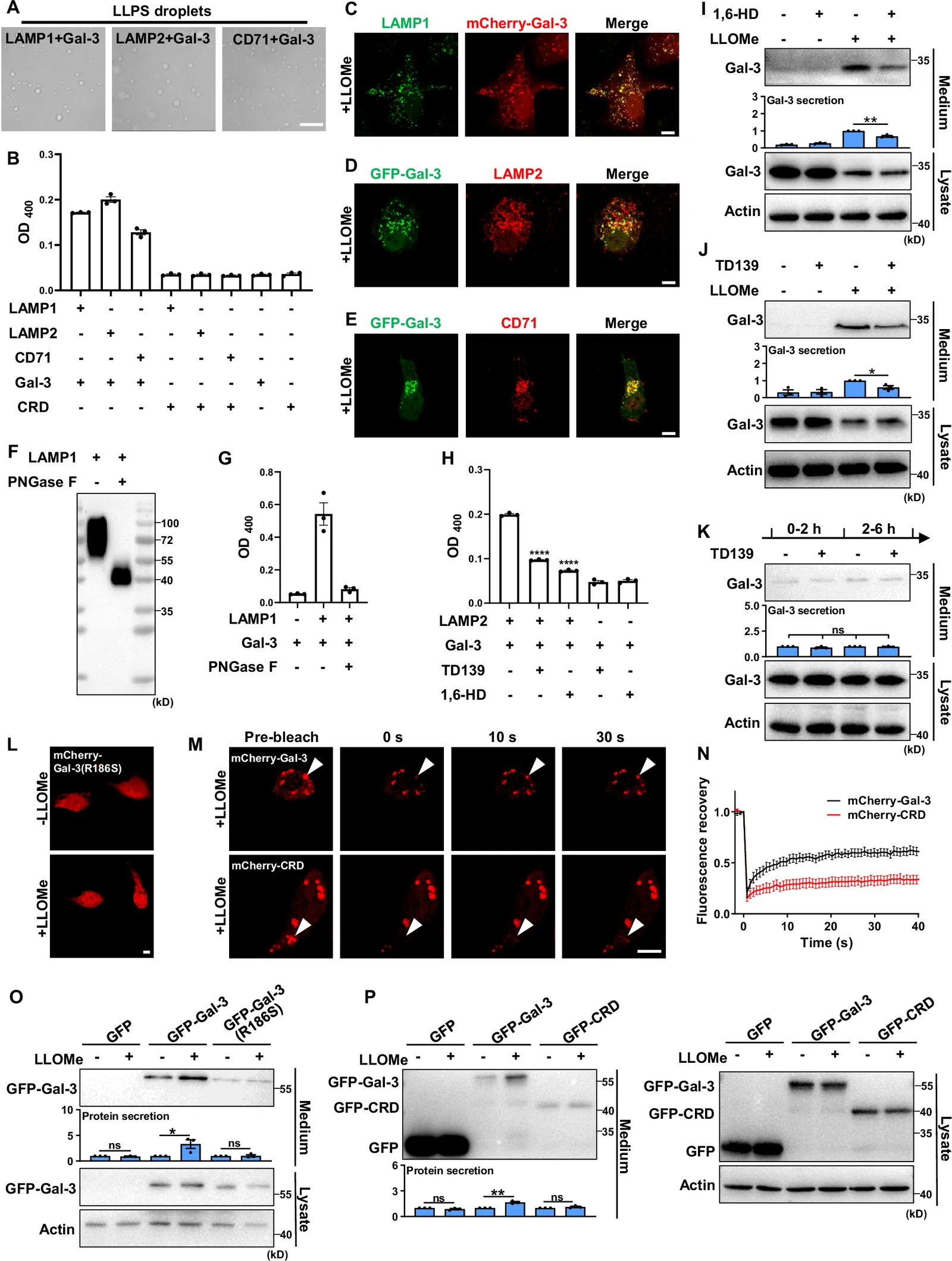
Gal-3 secretion is mediated by glycoprotein-induced Gal-3 phase separation/condensation. **A** Representative images of LLPS droplets formed by 70 μM Gal-3 and 0.5 μM glycoproteins (LAMP1, LAMP2, or CD71). **B** Turbidity analysis of LLPS induced by 30 μM Gal-3 (or CRD) and glycoproteins (0.4 μM LAMP1, 0.4 μM LAMP2, or 0.6 μM CD71). *n* = 3 independent experiments. **C**–**E** Representative images showing co-localization of Gal-3 and LAMP1 (**C**), LAMP2 (**D**), or CD71 (**E**) in HeLa cells. **F** Treatment of LAMP1 with PNGase F. The enzymatic products were analyzed by Western blotting. **G** Turbidity analysis of LLPS induced by 10 μM Gal-3 and 50 nM LAMP1 or PNGase F-treated LAMP1. *n* = 3 independent experiments. **H** Inhibition of LAMP2-induced Gal-3 LLPS by TD139 and 1,6-hexanediol (1,6-HD). Mixtures containing 0.4 μM LAMP2, 30 μM Gal-3, 5 μM TD139, and/or 5% 1,6-HD were assessed by turbidity analysis. *n* = 3 independent experiments. **I** Inhibition of LLOMe-induced Gal-3 secretion by 1,6-HD. Bars indicate Gal-3 secretion. *n* = 3 independent experiments. **J** Inhibition of TD139 to LLOMe-induced Gal-3 secretion. Bars indicate Gal-3 secretion. *n* = 3 independent experiments. **K** Effect of TD139 on Gal-3 secretion. HeLa cells were treated with 1 μM TD139 for 2 h in SFM, followed by 4 h incubation in SFM after TD139 washout. Bars indicate Gal-3 secretion. *n* = 3 independent experiments. **L** Representative images of Gal-3 mutant R186S puncta in HeLa cells. Cells transfected with mCherry-Gal-3(R186S) plasmid were treated with 1 mM LLOMe for 30 min. **M**, **N** FRAP analysis. HeLa cells transfected with mCherry-Gal-3 or mCherry-CRD plasmid were treated with 1 mM LLOMe for 30 min. **M** Representative images. **N** Quantification of FRAP data. *n* = 8 independent experiments. **O**, **P** Effects of Gal-3 mutation and truncation on its secretion. U2OS cells transfected with GFP, GFP-Gal-3, or GFP-Gal-3(R186S) (**O**), or GFP-CRD (**P**) plasmid were treated with 1 mM LLOMe for 30 min. Bars indicate protein secretion. *n* = 3 independent experiments. Data are shown as the mean ± SEM. *p < 0.05, **p < 0.01, and ****p < 0.0001. Two-tailed Student’s *t* test. ns, not significant. Scale bars, 10 μm. Source data are provided as a Source Data file.

The observation that multiple glycoproteins contribute to Gal-3 secretion raised the question of how these glycoproteins regulate Gal-3 release in lysosome-damaged cells. Our previous work demonstrated that glycoproteins can induce Gal-3 phase separation or condensation^38^, with NT acting as a sticker and CRD as a spacer (Supplementary Fig. 6). Using a LLPS assay, we have shown here that the glycoproteins involved in Gal-3 secretion (i.e., LAMP1, LAMP2, and CD71) can indeed induce Gal-3 droplet formation in vitro (Fig. 3A, B) and co-localize with Gal-3 condensates in lysosome-damaged cells (Fig. 3C–E). Furthermore, de-glycosylation with PNGase F (cleaving both complex and high-mannose/hybrid glycans) abrogated the ability of glycoproteins to induce Gal-3 LLPS (Fig. 3F, G, and Supplementary Fig. 7A), whereas de-glycosylation with Endo H (cleaving only high-mannose/hybrid glycans) did not impair the capacity of glycoproteins to drive Gal-3 LLPS (Supplementary Fig. 7A–C). Thus, complex-type glycans on glycoproteins are critical for driving Gal-3 LLPS.

To determine whether Gal-3 secretion depends on phase separation, we employed two inhibitors: 1,6-hexanediol (1,6-HD), a broad-spectrum LLPS disruptor^51^, and 3,3′-deoxy-3,3′-bis-(4-[m-fluorophenyl]-1H-1,2,3-triazol-1-yl)-thio-digalactoside (TD139), a Gal-3-specific inhibitor that impairs glycoprotein-induced Gal-3 phase separation (Fig. 3H). Both compounds are membrane-permeable and act intracellularly^51,52^. Treatment with either 1,6-HD or TD139 significantly inhibited lysosomal damage-induced Gal-3 secretion (Fig. 3I, J), underscoring the importance of phase separation in this process. Notably, TD139 alone did not induce Gal-3 phase separation (Fig. 3H) or enhance Gal-3 secretion (Fig. 3K), despite its intracellular interaction with cytoplasmic Gal-3. These findings further support the conclusion that Gal-3 phase separation is essential for its secretion.

To reinforce this conclusion, we overexpressed mCherry- or green fluorescent protein (GFP)-tagged wild-type Gal-3, the Gal-3(R186S) mutant (that attenuates glycan binding to Gal-3), and a truncated Gal-3 CRD (that lacks the NT) in U2OS cells that express Gal-3 at low levels. Previous studies have identified R186 and the NT as being critical for glycoprotein-induced Gal-3 phase separation^38^. Consistent with this observation, we found that Gal-3 condensation was significantly impaired in the R186S mutant, and fluorescence recovery after photobleaching (FRAP, reflecting the dynamic properties of condensates) was markedly reduced in the truncated CRD mutant (Fig. 3L–N). Accordingly, secretion of GFP-Gal-3(R186S) and GFP-CRD was significantly lower than that of wild-type GFP-Gal-3 (Fig. 3O, P). Collectively, these results demonstrate that glycoprotein-induced Gal-3 phase separation/condensation mediates Gal-3 secretion in lysosome-damaged cells.

### Phase-separated Gal-3 recruits ALG-2 to mediate Gal-3 secretion

**Fig. 4:**
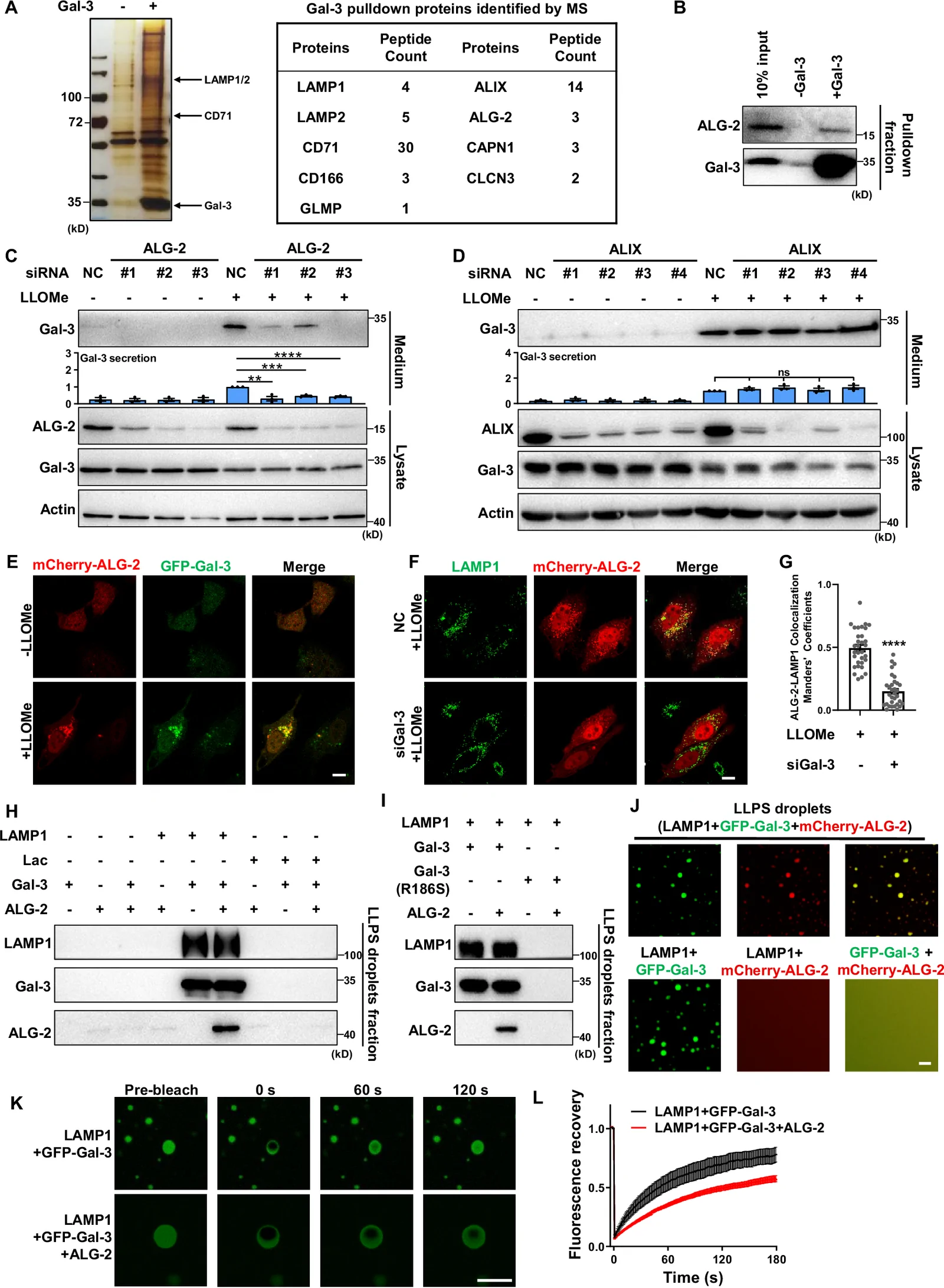
Gal-3 droplets/condensates recruit ALG-2 to mediate Gal-3 secretion. **A** Screening of Gal-3-interacting proteins. His-Gal-3 was immobilized on protein A/G magnetic beads pre-coated with anti-His antibody. HeLa cell lysates were incubated with Gal-3-coated beads. Pulled-down proteins were analyzed by silver staining (left) and mass spectrometry (MS, right). **B** Pull-down assays showing Gal-3 interaction with endogenous ALG-2. Gal-3 was immobilized on lactose beads and incubated with HeLa cell lysates. Pulled-down proteins were detected by Western blotting.  **C, D **Gal-3 secretion in ALG-2 knockdown (**C**) and ALIX knockdown (**D**) HeLa cells. Cells were treated with 1 mM LLOMe for 30 min, followed by a 4 h incubation in SFM after LLOMe washout. Medium and cell lysates were analyzed by Western blotting. Bars indicate Gal-3 secretion. *n* = 3 independent experiments. **E** Co-localization of ALG-2 and Gal-3. HeLa cells transfected with GFP-Gal-3 and mCherry-ALG-2 plasmids were treated with 1 mM LLOMe for 30 min and analyzed by confocal microscopy. **F**, **G** Gal-3 knockdown reduces co-localization of LAMP1 and ALG-2. HeLa cells transfected with mCherry-ALG-2 plasmid were subjected to Gal-3 knockdown, treated with 1 mM LLOMe for 30 min, and immunostained for LAMP1. **F** Representative images. **G** Quantification of LAMP1 and ALG-2 co-localization. *n* = 45 fields from three independent experiments. **H**, **I** Recruitment of ALG-2 to Gal-3 droplets. LAMP1 (1 μM) or lactose (Lac, 50 mM), Gal-3 (20 μM) or Gal-3(R186S) (20 μM), and ALG-2 (20 μM) were mixed and analyzed for LLPS droplet formation via Western blotting. **J** Representative images of LLPS droplets. LLPS droplets generated by mixing LAMP1 (0.6 μM), Gal-3 (24 μM), GFP-Gal-3 (4.2 μM), and mCherry-ALG-2 (20 μM) were visualized using confocal microscopy. **K**, **L** ALG-2 stabilizes Gal-3 droplets. LAMP1-induced Gal-3 droplets with or without ALG-2 were subjected to FRAP analysis. **K** Representative FRAP images. **L** Quantitative FRAP data. *n* = 8 independent experiments. Data are shown as the mean ± SEM. **p < 0.01, ***p < 0.001, and ****p < 0.0001. Two-tailed Student’s *t* test. ns, not significant. Scale bars, 10 μm. Source data are provided as a Source Data file.

At sites of lysosomal damage, the local concentration of Gal-3 increases markedly due to phase separation. We hypothesized that these Gal-3 condensates may recruit additional functional proteins to facilitate their secretion. To identify potential interacting partners, we conducted pulldown assays using Gal-3-immobilized beads incubated with cell lysates, followed by SDS-PAGE and MS analysis. As shown in Fig. 4A and Supplementary Data 2, several proteins were enriched in the bound fraction, including glycoproteins LAMP1, LAMP2, CD71, and CD166, as well as known Gal-3 interactors, such as calpain 1 (CAPN1), chloride channel protein 3 (CLCN3), and ALG-2-interacting protein X (ALIX). Notably, a cytoplasmic protein, ALG-2, was also identified and further validated (Fig. 4B). Among these proteins, ALG-2 and ALIX drew our attention because both are known to localize to damaged lysosomes^36,53^. We first investigated whether they contribute to Gal-3 secretion. As shown in Fig. 4C, D, ALG-2 knockdown significantly inhibited Gal-3 secretion, whereas knocking down ALIX had no effect. This indicates that ALG-2, but not ALIX, plays a crucial role in lysosomal damage-induced Gal-3 secretion, even though ALIX is known to participate in constitutive Gal-3 secretion^54^.

We next asked whether Gal-3 recruits ALG-2 in lysosome-damaged cells. As shown in Fig. 4E, ALG-2 co-localized with Gal-3 condensates in damaged, but not in untreated cells, suggesting that Gal-3 condensation is necessary for ALG-2 recruitment. Furthermore, ALG-2 co-localized with glycoproteins involved in Gal-3 condensation, such as LAMP1 (Fig. 4F), and this co-localization was lost upon Gal-3 knocking down (Fig. 4F, G). These findings indicate that ALG-2 recruitment is dependent on glycoprotein-induced Gal-3 condensation. To validate this, we performed LLPS droplet assays using purified ALG-2, Gal-3, and LAMP1. Substantial ALG-2 recruitment into Gal-3 droplets occurred only when all three components were present (Fig. 4H). Notably, when LAMP1 was replaced with lactose (Lac, a disaccharide known to bind to Gal-3 but not to induce phase separation), ALG-2 was not recruited into the droplets (Fig. 4H). Furthermore, replacement of WT Gal-3 with Gal-3(R186S), a mutant that does not induce phase separation (Fig. 3L), disrupted ALG-2 recruitment (Fig. 4I). These findings emphasize the necessity of Gal-3 phase separation for ALG-2 recruitment. Microscopy further confirmed the co-localization of ALG-2 and Gal-3 within the droplets (Fig. 4J). Finally, FRAP analysis revealed that ALG-2 recruitment reduced the dynamic exchange rate of Gal-3 (Fig. 4K,L), suggesting that ALG-2 stabilizes Gal-3 condensates.

### Molecular insight into LAMP1, Gal-3, and ALG-2 associations

**Fig. 5:**
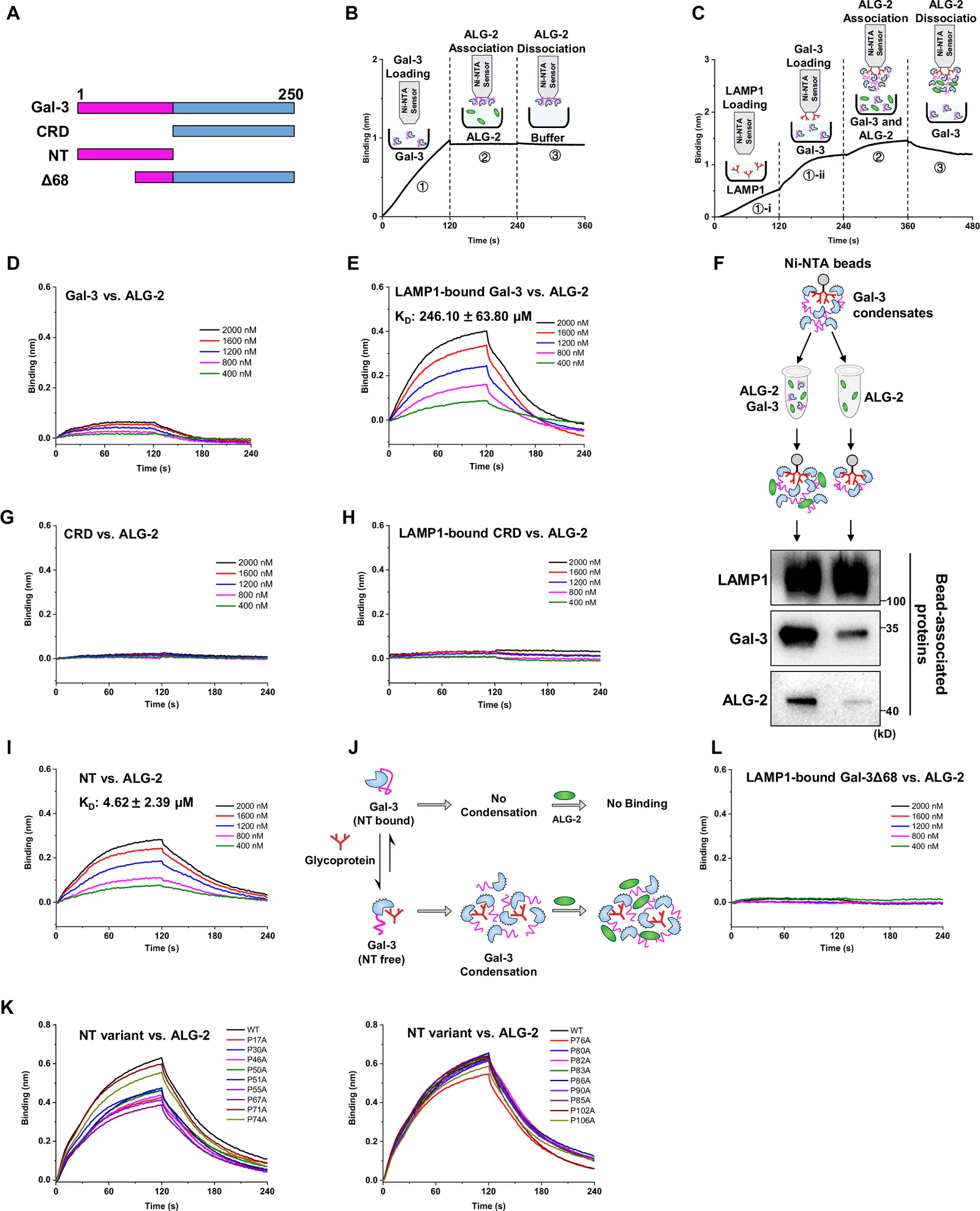
Interactions between Gal-3 and ALG-2. **A** Schematic diagram of wild-type (WT) Gal-3 and its truncation variants. **B**, **C** Schematic illustration of biolayer interferometry (BLI). **B** Direct immobilization of His-tagged Gal-3 (or its variants) onto Ni-NTA sensors. **C** Indirect immobilization of untagged Gal-3 (or its variants) onto Ni-NTA sensors via His-tagged LAMP1. **D**, **E** BLI analysis of interactions between Gal-3 and ALG-2. Gal-3 was either directly immobilized onto Ni-NTA sensors via His-tags or indirectly immobilized through binding to pre-immobilized LAMP1. The sensors were then exposed to increasing concentrations of ALG-2. **D** Direct interaction between full-length Gal-3 and ALG-2. **E** Interaction between LAMP1-bound Gal-3 and ALG-2. **F** Recruitment of ALG-2 to Gal-3 condensates. Ni-NTA beads immobilized LAMP1 were first incubated with Gal-3 and then exposed to ALG-2 in the presence or absence of Gal-3, and bound proteins were analyzed by Western blotting. **G**–**I** BLI analysis of interactions between Gal-3 truncates and ALG-2. Gal-3 truncates were either directly immobilized onto Ni-NTA sensors via His-tags or indirectly immobilized through binding to pre-immobilized LAMP1. The sensors were then exposed to increasing concentrations of ALG-2. **G** Interaction between Gal-3 C-terminal carbohydrate recognition domain (CRD) and ALG-2. **H** Interaction between LAMP1-bound CRD and ALG-2. **I** Interaction between Gal-3 N-terminal domain (NT) and ALG-2. **J** Proposed model illustrating interactions among glycoprotein, Gal-3, and ALG-2. **K** BLI analysis of interactions between ALG-2 and proline-mutant NT. Equivalent amounts of WT or mutant NT were immobilized on Ni-NTA sensors via His-tags and exposed to ALG-2. **L** BLI analysis of interactions between LAMP1-bound Gal-3Δ68 and ALG-2. Gal-3Δ68 was immobilized via binding to pre-immobilized LAMP1 and exposed to increasing concentrations of ALG-2. Source data are provided as a Source Data file.

The association between LAMP1, Gal-3, and ALG-2 was further investigated using biolayer interferometry (BLI). Our BLI assay consists of three main steps: (1) loading Gal-3 variants onto Ni-NTA sensors either directly (via interaction of their His-tags with the Ni-NTA sensors, Fig. 5B) or indirectly (via interaction with the pre-immobilized glycoprotein LAMP1, Fig. 5C), (2) dipping the sensors in buffer containing ALG-2 variants to monitor association, and (3) dipping the sensors in buffer without ALG-2 variants to monitor dissociation. For clarity, partial sensorgrams with only association and dissociation steps are shown. Here, we found that directly loaded full-length Gal-3 does not interact with ALG-2 (Fig. 5A, D). However, when Gal-3 is indirectly loaded via LAMP1, it subsequently interacts with ALG-2, with a dissociation constant (*K*_D_) of 246.10 ± 63.80 μM (Fig. 5E). This indicates that LAMP1 binding is a prerequisite for the interaction between Gal-3 and ALG-2. These findings are consistent with our LLPS droplet assay that showed that ALG-2 is recruited into droplets only when both Gal-3 and glycoproteins are present (Fig. 4H).

It is worth noting that, in the indirect loading method, Gal-3 was included throughout the loading, association, and dissociation steps to maintain constant surface levels of Gal-3 (Fig. 5C); otherwise, a fraction of loaded Gal-3 molecules quickly dissociated from the sensors. This dynamic behavior is consistent with our previous findings that glycoproteins immobilized on solid surfaces (like sensors or latex beads) can induce Gal-3 phase separation^38^. In this regard, the data from the indirect method reflect/represent the interaction between Gal-3 condensates and ALG-2. The process and rationale of the indirect method can be mimicked easily with Ni-NTA beads and test tubes. As illustrated in Fig. 5F, we loaded Gal-3 onto Ni‑NTA beads via pre-immobilized LAMP1 and then placed the beads in tubes containing ALG-2 with or without Gal-3, followed by detection of bead-associated Gal-3 and ALG-2. Results showed that the content of bead-associated Gal-3 markedly reduced in the tube without Gal-3, an indication of the dynamic nature of bead-associated Gal-3. In this case, ALG-2 recruitment was also markedly impaired. Together, these results support our finding that ALG-2 association depends on Gal-3 condensates.

To determine which region of Gal-3 mediates ALG-2 binding, we examined interactions using a truncated CRD and the NT peptide. Whereas the CRD showed no binding to ALG-2 regardless of the presence or absence of glycoprotein (Fig. 5G, H), the isolated NT peptide exhibited binding to ALG-2 even in the absence of LAMP1 (*K*_D_ = 4.62 ± 2.39 μM) (Fig. 5I). These results identify the NT region of Gal-3 as the ALG-2 binding domain. This raises the question: why does the full-length Gal-3 fail to bind ALG-2 unless pre-bound to LAMP1, whereas the isolated NT peptide does? A plausible explanation is that, in the full-length Gal-3 protein, the NT transiently interacts with the F-face of the CRD^18^, masking ALG-2 binding sites. Upon binding to glycoproteins, conformational changes in the CRD alter this CRD-NT equilibrium, favoring the NT-free state^18^, which exposes the ALG-2 binding region and facilitates interaction between the NT and ALG-2 (Fig. 5J). We refer to this mechanism as glycoprotein-activated binding to distinguish it from the intrinsic binding characteristic of intact Gal-3.

Our previous work has shown that proline residues in the NT are essential for Gal-3 function^38^. To identify which NT prolines contribute to ALG-2 interaction, we used a library of proline-to-alanine NT mutants. We found that proline residues near the N-terminus (P17, P30, P46, P50, P51, and P55) are essential for ALG-2 binding. These mutant NTs displayed substantially reduced ALG-2 binding signals compared to WT NT under identical measuring conditions (Fig. 5K). In accordance with this, we found that these mutants showed weaker binding affinities than WT NT (Supplementary Fig. 8). In contrast, we found that mutation of downstream prolines (P71, P74, P76, P80, P82, P83, P86, P90, P95, P102, and P106) had little effects on binding signals (Fig. 5K), indicating that these prolines are not involved in ALG-2 binding. This aligns with our observation that deletion of the first 68 NT residues abrogates ALG-2 binding (Fig. 5L), further confirming that the NT portion (residues 1–68) is critical for this interaction.

**Fig. 6:**
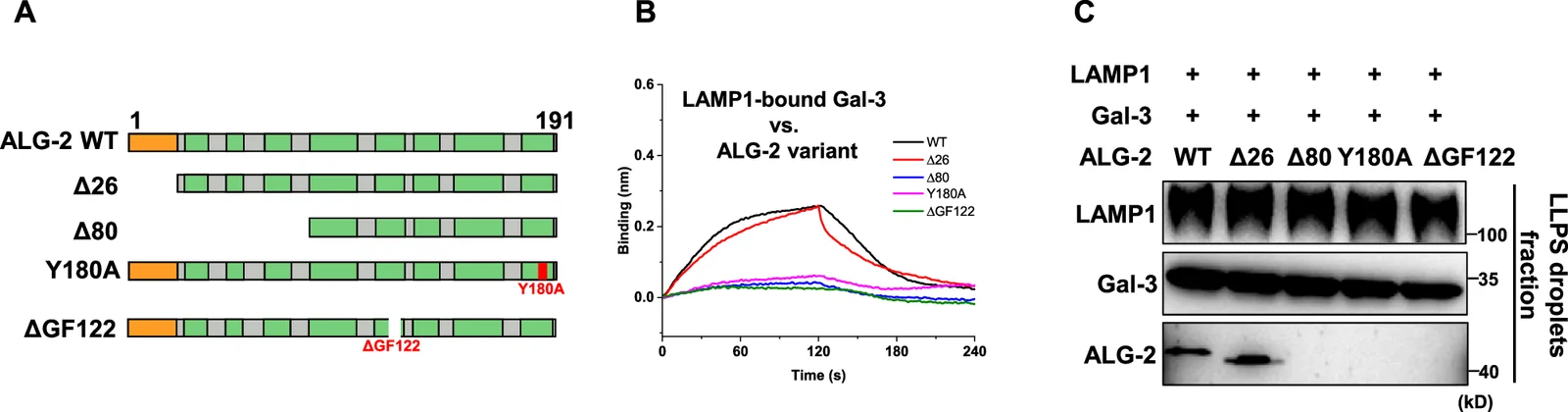
Identification of Gal-3 binding sites on ALG-2. **A** Schematic illustration of WT ALG-2 and its mutant variants. **B** BLI analysis of interactions between LAMP1-bound Gal-3 and ALG-2 mutants. Gal-3 was immobilized via binding to LAMP1, and sensors were exposed to 2 μM WT or mutant ALG-2. **C** Recruitment of ALG-2 mutants to Gal-3 droplets. LLPS droplets generated from the mixture of LAMP1 (400 nM), Gal-3 (20 μM), and WT or mutant ALG-2 (20 μM) were analyzed by Western blotting. Source data are provided as a Source Data file.

To map Gal-3 binding sites on ALG-2, we generated several ALG-2 truncation and point mutants targeting the three previously identified binding pockets^55,56^ (Fig. 6A). BLI analysis revealed that LAMP1-activated full-length Gal-3 binds to ALG-2 variant Δ26, but not to variants Δ80 (associated with pocket 3), ΔGF122 (pocket 2), or Y180A (pocket 1) (Fig. 6B). These data suggest that all three binding pockets on ALG-2 are required for effective interaction with Gal-3 and for recruitment into LAMP1-induced Gal-3 droplets (Fig. 6C).

### ALG-2 directs Gal-3 condensates to the ER and late endosomes

We next investigated the role of ALG-2 in Gal-3 secretion. Previous studies have shown that ALG-2 can localize in the ER and late endosomes (LEs) in some circumstances^57,58^. This prompted us to examine the subcellular localization of Gal-3 before and after lysosomal damage.

**Fig. 7:**
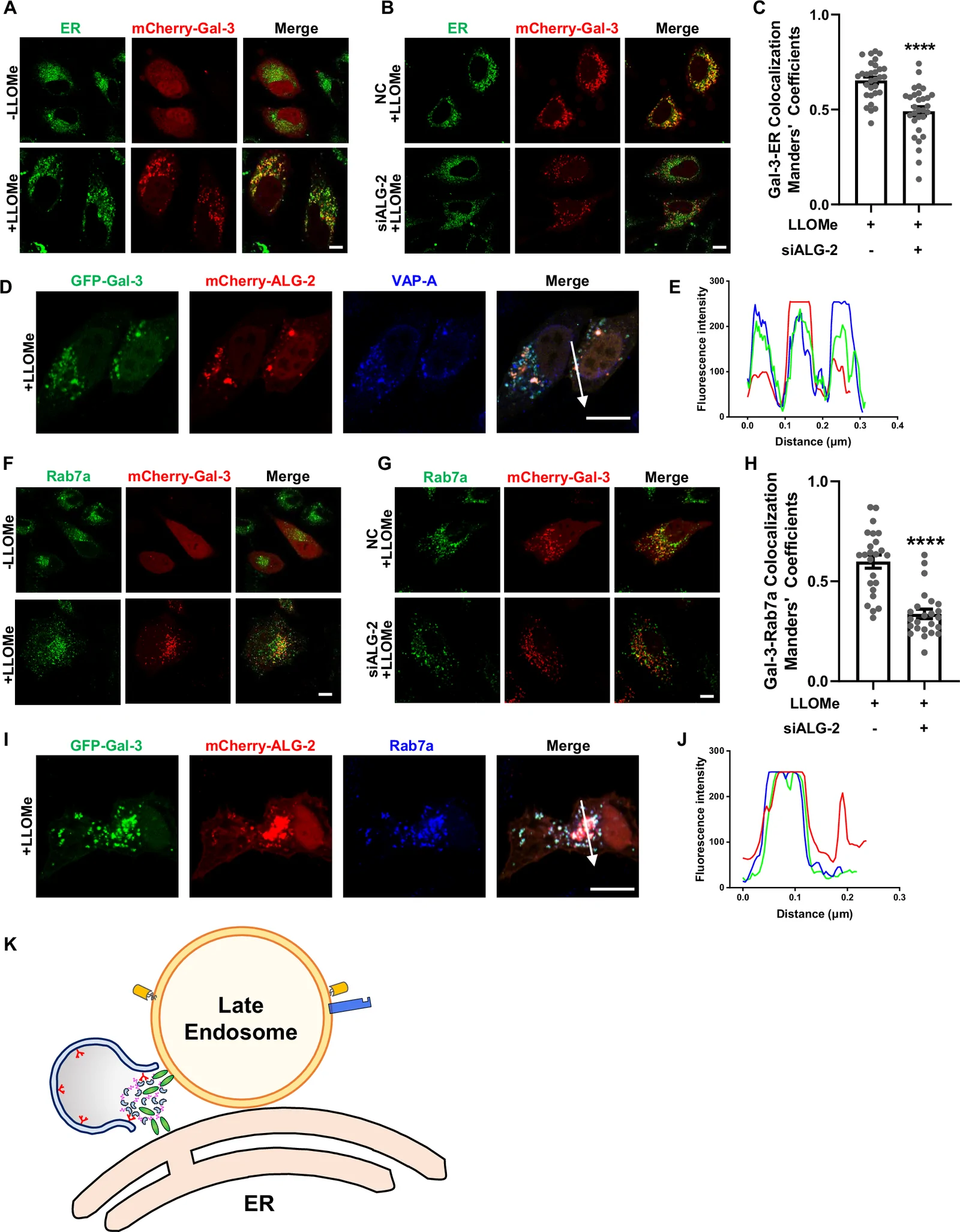
Gal-3 is targeted to the ER and late endosomes (LEs) by recruiting ALG-2. **A** Representative confocal images showing co-localization of Gal-3 and the endoplasmic reticulum (ER). HeLa cells transfected with the mCherry-Gal-3 plasmid were treated with 1 mM LLOMe for 30 min and stained with ER-Tracker™ Green. **B**, **C** ALG-2 knockdown reduces co-localization of Gal-3 and the ER. HeLa cells transfected with the mCherry-Gal-3 plasmid were subjected to ALG-2 knockdown, treated with 1 mM LLOMe for 30 min, and stained with ER-Tracker™ Green. **B** Representative images. **C** Quantification of Gal-3 and ER co-localization. *n* = 45 fields from three independent experiments. **D**, **E** Representative images showing co-localization of VAP-A, ALG-2, and Gal-3. HeLa cells transfected with GFP-Gal-3 and mCherry-ALG-2 plasmids were treated with 1 mM LLOMe for 30 min and immunostained with anti-VAP-A antibody. **D** Representative images. **E** Line scan analysis of fluorescence intensity across the area indicated by the white arrow in the merged image. **F** Representative images showing co-localization of Gal-3 and Rab7a in HeLa cells. Cells transfected with mCherry-Gal-3 plasmid were treated with 1 mM LLOMe for 30 min and immunostained with antibody against Rab7a. **G**, **H** ALG-2 knockdown impairs co-localization of Rab7a and Gal-3. HeLa cells transfected with mCherry-Gal-3 plasmid were subjected to ALG-2 knockdown, treated with 1 mM LLOMe for 30 min and immunostained with anti-Rab7a antibody. **G** Representative images. **H** Quantification of co-localization between Rab7a and Gal-3. *n* = 45 fields from three independent experiments. **I**, **J** Co-localization of Rab7a, ALG-2, and Gal-3. HeLa cells transfected with GFP-Gal-3 and mCherry-ALG-2 plasmids were treated with 1 mM LLOMe for 30 min and immunostained with anti-Rab7a antibody. **I** Representative images. **J** Line scan analysis of fluorescence intensity across the area indicated by the white arrow in the merged image. **K** Schematic model depicting the dual role of ALG-2 in targeting Gal-3 to both the ER and LEs. Data are presented as mean ± SEM. ****p < 0.0001, two-tailed Student’s *t* test. Scale bars, 10 μm. Source data are provided as a Source Data file.

Using a pan-ER marker (ER-Tracker™ Green), we observed that Gal-3 co-localizes with the ER in lysosome-damaged cells, but not under normal conditions (Fig. 7A). This ER co-localization is significantly diminished upon ALG-2 knockdown (Fig. 7B, C), indicating that ALG-2 is required for directing Gal-3 condensates to the ER. Supporting this conclusion, Gal-3, ALG-2, and VAP-A (another ER marker) co-localize in lysosome-damaged cells (Fig. 7D, E).

Recent studies have demonstrated that Tau and α-synuclein (proteins that lack signal peptides) can be secreted via LEs that either mature on the ER or remain in close association with it^11,59,60^. This prompted us to investigate whether Gal-3 is similarly transferred from the ER to LEs. Localization studies revealed that Gal-3 co-localizes with Ras-related protein Rab7a (an LE marker) in lysosome-damaged, but not in undamaged cells (Fig. 7F). Such co-localization is significantly reduced in ALG-2 knockdown cells (Fig. 7G, H). Triple staining confirmed co-localization of Gal-3 with both Rab7a and ALG-2 in lysosome-damaged cells (Fig. 7I, J). These data suggest that ALG-2 also facilitates targeting of Gal-3 condensates to LEs.

Together, these results suggest a dual role for ALG-2: guiding Gal-3 condensates to both the ER and LEs, potentially through ER–LE membrane contact sites (Fig. 7K).

### Gal-3 is secreted via LE-mediated vesicular transport

**Fig. 8:**
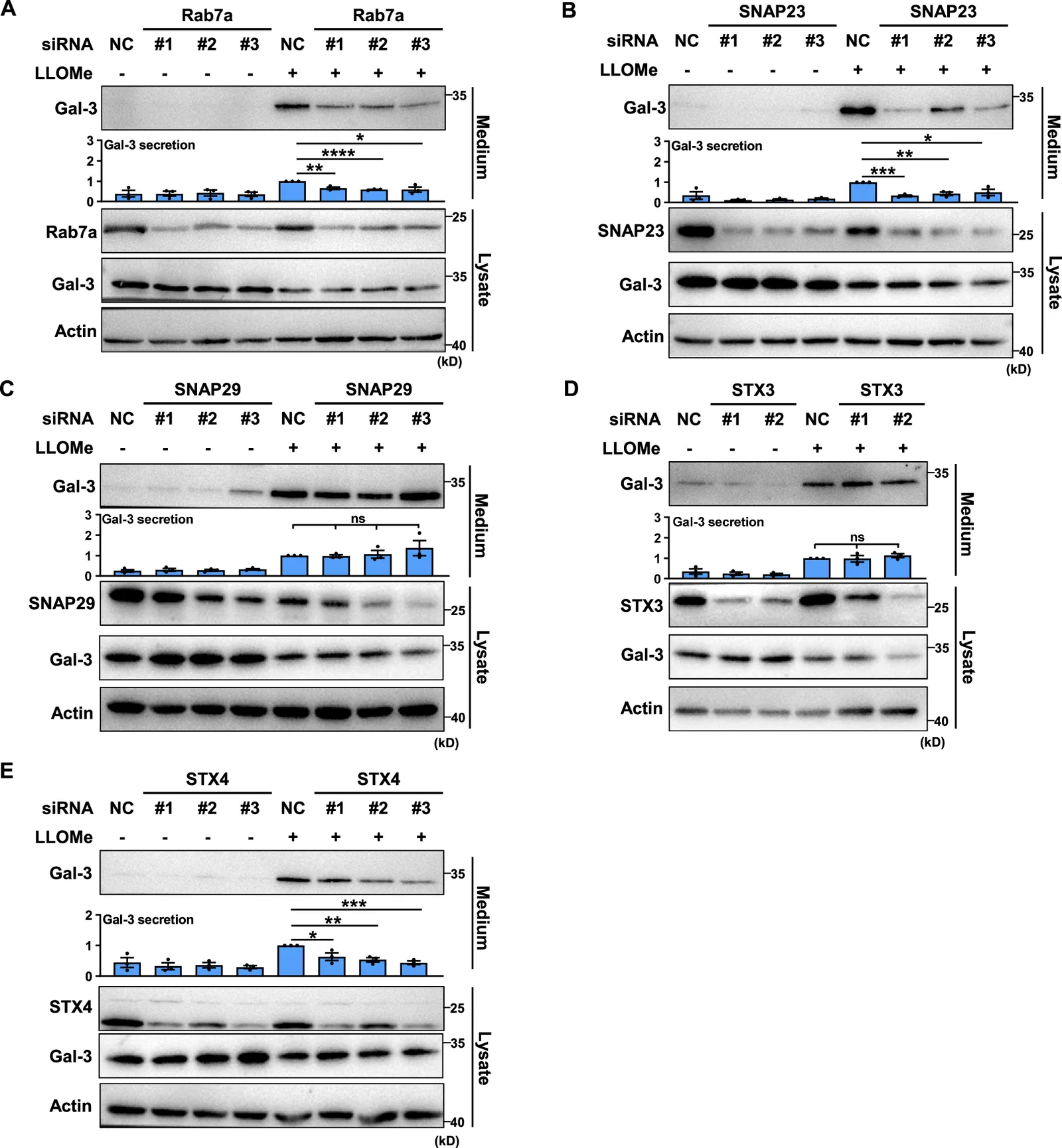
Gal-3 secretion depends on LE-PM trafficking. Gal-3 secretion in HeLa cells following knockdown of Rab7a (**A**), SNAP23 (**B**), SNAP29 (**C**), STX3 (**D**), and STX4 (**E**). Cells were treated with 1 mM LLOMe for 30 min, followed by 4 h incubation in SFM after LLOMe washout. Gal-3 levels in the medium and protein levels in the cell lysate were analyzed by Western blotting. Bars indicate Gal-3 secretion. *n* = 3 independent experiments. Data are shown as the mean ± SEM. *p < 0.05, **p < 0.01, ***p < 0.001, and ****p < 0.0001, two-tailed Student’s *t* test. ns, not significant. Source data are provided as a Source Data file.

**Fig. 9:**
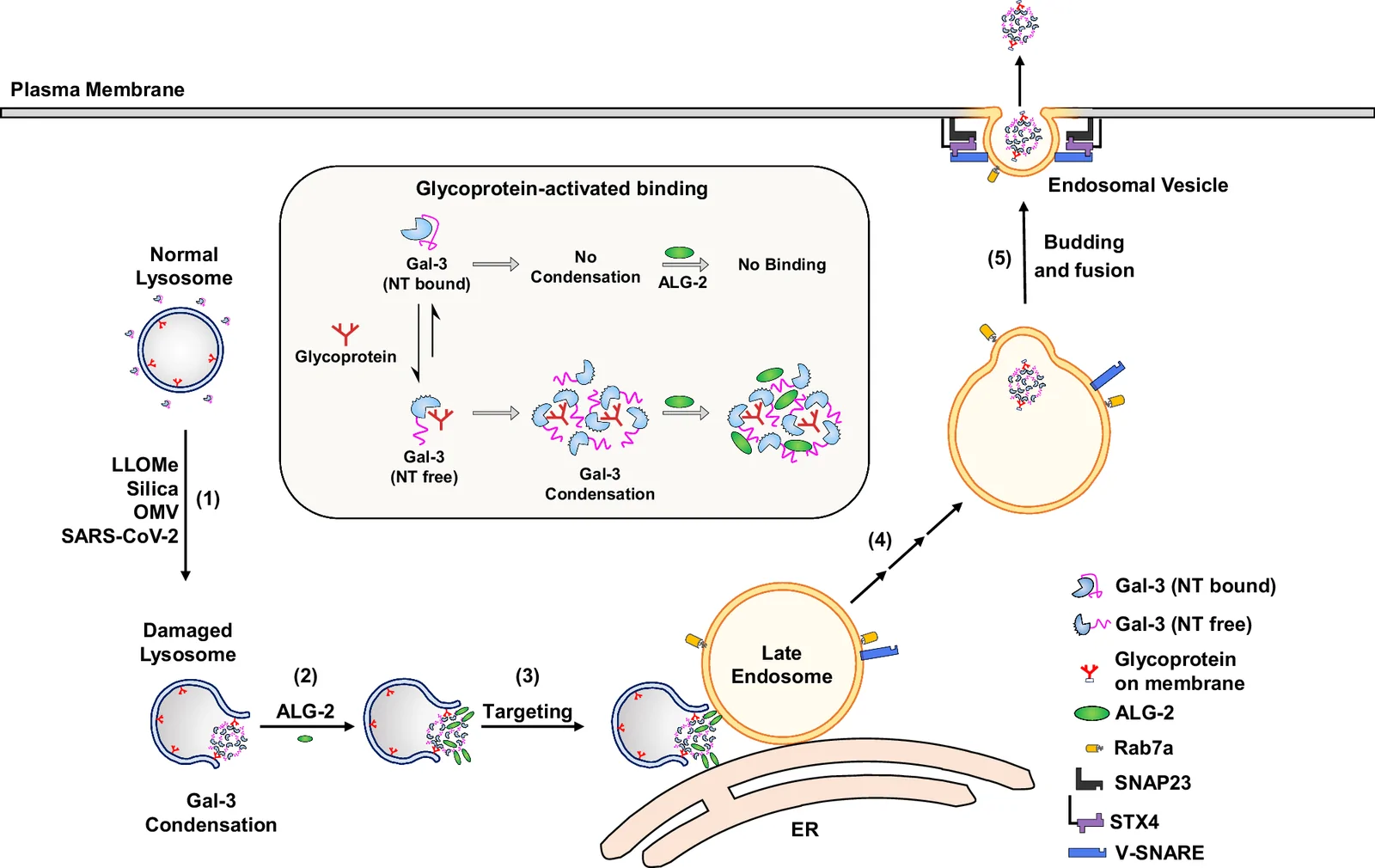
Proposed model of Gal-3 secretion. (1) Exposed glycoproteins within damaged lysosomes bind and recruit cytosolic Gal-3. Glycoprotein binding induces conformational changes in the F-face of the Gal-3 CRD, leading to Gal-3 NT release and condensation. (2) Gal-3 molecules within these condensates use their free NTs to recruit ALG-2. (3) ALG-2 directs the condensates to the interface between the ER and LEs. (4) The Gal-3 condensates are translocated into the LEs. (5) Finally, as cargo, Gal-3 condensates within LEs are delivered to the plasma membrane and released into the extracellular milieu by SNARE-dependent vesicular transport.

Identification of Gal-3 localized at the LE suggests that Gal-3 is likely secreted from this organelle. In line with this suggestion, we found that knocking down Rab7a (a key functional molecule in LE-mediated vesicular transport) significantly inhibited Gal-3 secretion (Fig. 8A). Furthermore, knockdown of key molecules involved in LE-to-plasma membrane (PM) trafficking (synaptosomal-associated protein (SNAP)23, and syntaxin 4 (STX4)) led to a marked reduction in Gal-3 secretion (Fig. 8B, E). Here, SNAP29 and STX3 were used as negative controls (Fig. 8C, D). These results suggest that Gal-3 molecules (in the form of condensates) are loaded into Rab7a-positive vesicles, which traffic to the PM and subsequently fuse with the membrane via SNAP23 and STX4, releasing Gal-3 condensates into the extracellular space (Fig. 9). This hypothesis is consistent with our observation that Gal-3 molecules secreted upon lysosome damage were all present in the non-membranous macromolecular particles (Fig. 1M–O).

## Discussion

UcPS plays a critical role in cellular responses to stress and various pathological conditions^61,62^. The pathways, molecular triggers, and mechanisms of UcPS are diverse and context-dependent, varying with physiological and pathological states. In this study, we investigated the UcPS of Gal-3, whose extracellular secretion is elevated in several pathological disorders^24,26–28^. We identified a distinct mechanism of unconventional Gal-3 secretion in lysosome-damaged cells. Uniquely, this UcPS pathway is driven by glycoprotein-triggered phase separation of Gal-3, representing a mechanism distinct from previously proposed models.

This UcPS pathway involves the following. (1) Exposed glycoproteins within damaged lysosomes bind and recruit cytosolic Gal-3. Glycoprotein binding induces conformational changes in the F-face of the Gal-3 CRD, leading to Gal-3 NT release and condensation. (2) Gal-3 molecules within these condensates use their free NTs to recruit ALG-2. (3) ALG-2 directs the condensates to the interface between the ER and LEs. (4) The Gal-3 condensates are translocated into the LEs. (5) Finally, as cargo, Gal-3 condensates within LEs are delivered to the plasma membrane and released into the extracellular milieu by SNARE-dependent vesicular transport. We demonstrated this mechanism using both LLOMe- and silica-induced lysosomal damage models in HeLa and A549 cells (Figs. 2E, 4C, and Supplementary Fig. 9A–D). The observation of this UcPS pathway in different cell types suggests it is broadly applicable. By linking glycoprotein-induced phase separation with UcPS, our findings establish a mechanistic framework for Gal-3 secretion and highlight LLPS as a central organizing principle in the UcPS response to lysosomal stress.

LLPS is an emerging biological phenomenon implicated in cellular processes, such as proteasomal degradation and autophagy, DNA damage and repair, and regulation of gene expression^39–41^. Here, we report a role for LLPS in UcPS. While a previous study showed that Grh1, a Golgi matrix protein involved in UcPS, can undergo phase separation in vitro^63^, our work establishes a direct functional link between phase separation and UcPS. We demonstrated that glycoprotein-induced phase separation is indispensable for the unconventional secretion of Gal-3. First, disruption of phase separation using 1,6-HD or TD139 inhibited Gal-3 secretion (Fig. 3I, J). Second, structural modifications to Gal-3, such as mutation or truncation, which impair its ability to undergo phase separation, also reduced Gal-3 secretion (Fig. 3O,P). Third, TD139—a glycan that interacts with Gal-3 but does not induce its phase separation—failed to promote Gal-3 secretion (Fig. 3H, K). Finally, only phase-separated Gal-3 condensates served as a platform to recruit the functional molecule ALG-2, essential for secretion, whereas monomeric Gal-3 was unable to recruit it (Fig. 4H). Although our findings show that phase-separated Gal-3 condensates mediate their own secretion, this mechanism may also be co-opted or hijacked by other cytoplasmic proteins that interact with the system, such as Gal-1 and various glycoproteins. Preliminary data from our group suggest that Gal-3 plays a role in the secretion of Gal-1 (Supplementary Fig. 10) and certain glycoproteins (Fig. 2I). Further investigations into this phenomenon are currently underway in our laboratory.

Here, we report a distinct interaction mode of Gal-3, namely glycoprotein-activated interaction. In this context, glycoprotein-treated full-length Gal-3 binds ALG-2, whereas untreated Gal-3 does not. Glycoprotein functions as a key that unlocks structural changes in Gal-3, the lock. Upon glycoprotein engagement, Gal-3 undergoes conformational and dynamic changes that enable interactions with other molecules. In contrast, without glycoprotein binding, Gal-3 co-exists with this molecule in the cytoplasm without interaction. Notably, glycoprotein-binding not only exposes ALG-2 binding sites but also triggers Gal-3 condensation. Efficient ALG-2 binding relies on both changes. The importance of Gal-3 condensation in ALG-2 binding is highlighted in two experiments: (1) without Gal-3 condensation, as shown in the droplet assay, ALG-2 binding was not observed (Fig. 4H), and (2) disruption of Gal-3 condensation, as shown in the binding assay (Fig. 5F), recruitment of ALG-2 is minimal. This mode of glycoprotein-activated interaction may extend to other Gal-3-associated molecules (e.g., ALIX and TRIM16)^36,64^.

Early studies suggested that Gal-3 is secreted via exosomes^12,65^. However, in our system, secreted Gal-3 is not membrane-enclosed, indicating that it is not released through the exosome pathway. Given that Gal-3 forms large particles or condensates under lysosomal damage—structures that may be harmful to cells—it is not surprising that cells might favor a more efficient secretion mechanism over the relatively slow and steady exosome-mediated pathway that typically facilitates constitutive secretion^66^. Autophagy has also been implicated in Gal-3 secretion^46^. However, in cells with lysosomal damage, autophagic flux is disrupted. Interestingly, autophagy and secretion appear to compete with each other: inhibition of autophagy or knockdown of autophagy-related proteins (e.g., TRIM16 and ATG16L1) increases Gal-3 secretion (Supplementary Fig. 5A–D). Recent studies have shown that gasdermin D (GSDMD) drives Gal-3 secretion in LPS/ATP-stimulated bone marrow-derived macrophages (BMDMs)^43^. In our system, however, GSDMD is not activated (Supplementary Fig. 4A). The differing experimental reagents and models likely account for these discrepancies. For example, LPS binds Gal-3^67^ and may produce different outcomes compared to the use of LLOMe or silica to induce lysosomal damage. Although GSDME is activated in our model, this activation is unrelated to Gal-3 secretion (Supplementary Fig. 4A–C). Gal-3 can also be secreted via a vesicular transport pathway mediated by TMED10 in HEK293T cells^7^, while ERGIC is a critical intermediate compartment for the TMED10-channelled UcPS^7^. In our model, however, Gal-3 does not colocalize with the ERGIC marker ERGIC53 and knockdown of TMED10 does not affect Gal-3 secretion (Supplementary Fig. 11). Thus, lysosomal damage-induced Gal-3 secretion differs from the ERGIC and TMED10-mediated UcPS, and distinct secretion pathways are activated in response to different cellular conditions.

In our model, Gal-3 condensates are secreted by LE-mediated vesicular transport in accordance with the observation that the secretion depends on Gal-3 LE localization and Rab7a, a key functional protein in LE-mediated vesicular transport (Figs. 7F–H and 8A). However, the mechanism by which Gal-3 enters the LE compartment remains unclear. Invagination is a well-known mechanism implicated in translocating cytosolic proteins or aggregates into membranous compartments. However, this mechanism seems unlikely, because invagination of Gal-3 condensates would result in membrane-enclosed Gal-3 vesicles within a membranous compartment (i.e., multivesicular bodies), leading to extracellular vesicle release that contradicts our findings (Fig. 1O). Certain unconventionally secreted cytosolic proteins can access membrane-bound compartments by translocation mechanisms that involve transient pore formation by membrane-associated factors^7,11^. This raises the possibility that a similar mechanism may drive the entry of Gal-3 into LEs. In addition, lysosome–LE fusion occurs during endo-lysosomal trafficking and organelle maturation^68^. This fusion process may represent an additional route through which Gal-3 condensates are delivered into membrane-enclosed compartments. To resolve this issue, further investigations are required.

Here, we also found that *N*-glycans are pivotal to lysosomal damage-induced Gal-3 secretion. In normal cells, Gal-3 levels in culture medium are increased by tunicamycin treatment or by knocking down MGAT1, because they decrease cell surface glycoprotein levels and thus retain less Gal-3^69^. And although we do observe this in control cells (Fig. 2C, E), Gal-3 levels in the medium of lysosome-damaged cells (i.e., LLOMe or silica-treated cells) are decreased, and not increased, by tunicamycin treatment or by knocking down MGAT1 (Fig. 2C, E, and Supplementary Fig. 9A, C). Therefore, *N*-glycans play opposite roles in constitutive and lysosomal damage-triggered secretion. In the former, *N*-glycans are required to anchor secreted Gal-3 on the cell surface, whereas in the latter, *N*-glycans are required to mediate Gal-3 secretion itself. The results depicted here show that inhibition of *O*-glycosylation not only enhances constitutive secretion of Gal-3 in normal cells but also increases lysosomal damage-triggered Gal-3 secretion (Fig. 2D). Thus, *O*-glycans hinder secretion in both cases.

Different members of the galectin family, such as Gal-1 and Gal-8, can also be recruited to damaged lysosomes^35^, raising the question of whether Gal-1 and Gal-8 are secreted in a manner similar to Gal-3. Under the same conditions that induce Gal-3 secretion, Gal-8 neither forms puncta nor is secreted (Supplementary Fig. 10A, B). However, when LLOMe concentration and treatment duration are increased, Gal-8 forms detectable puncta, although this coincides with cell death (Supplementary Fig. 10C, D). In contrast, Gal-1 behaves more like Gal-3, forming visible puncta (Supplementary Fig. 10A). To explore a potential relationship between Gal-3 and Gal-1 secretion, we individually knocked down Gal-1 and Gal-3. We found that Gal-1 secretion is reduced upon Gal-3 depletion (Supplementary Fig. 10E), whereas Gal-3 secretion remains unaffected by Gal-1 knockdown (Supplementary Fig. 10F). These results suggest that Gal-1 secretion depends on Gal-3, but not vice versa. One possible explanation is that Gal-1 requires physical interaction with Gal-3 to be secreted, a hypothesis supported by previous studies reporting hetero-complex formation between Gal-1 and Gal-3^70–72^.

Gal-3 hypersecretion has emerged as a key factor in the development of several pathological disorders, including AD, pulmonary fibrosis, and COVID-19^21,27,73^. Excessive extracellular Gal-3 has been shown to amplify pro-inflammatory cascades during cytokine storms^74^ and mediate intercellular crosstalk between senescent endothelial cells and effector cells, contributing to fibrotic remodeling^75^. Although these studies establish Gal-3 as a pleiotropic effector of disease pathogenesis, the fundamental mechanism driving its pathological secretion has remained unresolved. Our results demonstrate that aberrant glycan signaling and condensate formation underlie the pathological accumulation of extracellular Gal-3. This finding not only elucidates the underlying mechanism of secretion but also identifies a potential therapeutic target in lysosomal disorder-associated diseases. Given the pivotal role of LLPS in Gal-3 secretion, disrupting LLPS using membrane-permeable inhibitors or drugs (e.g., TD139) may represent a promising therapeutic strategy.

Cellular homeostasis depends on multiple stress-adaptation pathways. Previous studies have shown that Gal-3, Gal-8, and Gal-9 are all recruited to damaged endomembranes, where they mediate autophagy—a classical response to membrane injury^35,64,76–78^. In contrast to autophagy-mediated intracellular degradation, the LLPS-driven UcPS pathway exports harmful Gal-3 condensates and membrane debris to the extracellular space. This form of Gal-3 secretion may represent a primordial clean-up mechanism for insoluble aggregates, functioning alongside autophagy in the cellular response to membrane damage. Our findings suggest that the UcPS pathway is not merely an alternative secretion route, but rather a critical fail-safe mechanism for maintaining proteostasis under lysosomal stress—bearing broad implications for diseases characterized by toxic condensate accumulation.

## Methods

### Antibodies and reagents

The primary antibodies used in this study were as follows: mouse anti-Actin (Cat# 612657, BD Transduction Laboratories), mouse anti-ALG-2 (Cat# H00010016-M01, Abnova, use for immunofluorescence), rabbit anti-ALG-2 (Cat# ab109181, Abcam, use for Western blotting), rabbit anti-ATG16L1 (Cat# 8089, Cell Signaling Technology), rabbit anti-Beclin1 (Cat# 3495, Cell Signaling Technology), rabbit anti-caspase-3 (Cat# 9662, Cell Signaling Technology), rabbit anti-CD44 (Cat# 37259, Cell Signaling Technology), rabbit anti-CD46 (Cat# ab108307, Abcam), rabbit anti-CD71 (Cat# ab214039, Abcam), rabbit anti-CD146 (Cat# ab75769, Abcam), rabbit anti-cleaved N-terminal GSDMD (Cat# ab215203, Abcam), rabbit anti-ERGIC53 (Cat# 13364-1-AP, Proteintech), rabbit anti-TMED10 (Cat# ab34948, Abcam), mouse anti-Flag (Cat# RM1002, Beijing Ray Antibody Biotech), rabbit anti-galectin-1 (Cat# 12936, Cell Signaling Technology), rabbit anti-galectin-3 (Cat# 87985, Cell Signaling Technology), mouse anti-galectin-8 (Cat# G5671, Sigma-Aldrich), rabbit anti-GFP (Cat# 2956, Cell Signaling Technology), rabbit anti-GSDME (Cat# ab215191, Abcam), mouse anti-His (Cat# RM1001, Beijing Ray Antibody Biotech), rabbit anti-LAMP1 (Cat# 9091, Cell Signaling Technology), rabbit anti-LAMP2 (Cat# MAB6228, R&D Systems, use for immunofluorescence), rabbit anti-LAMP2 (Cat# ab199946, Abcam, use for Western blotting), mouse anti-LC3B (Cat# 83506, Cell Signaling Technology), rabbit anti-MGAT1 (Cat# ab180578, Abcam), rabbit anti-Rab7 (Cat# 9367, Cell Signaling Technology), rabbit anti-SNAP23 (Cat# ab3340, Abcam), rabbit anti-SNAP29 (Cat# ab138500, Abcam), rabbit anti-syntaxin 3 (Cat# ab101744, Abcam), rabbit anti-syntaxin 4 (Cat# ab184545, Abcam), rabbit anti-TRIM16 (Cat# ab72129, Abcam), mouse anti-ULK1 (Cat# 8054, Cell Signaling Technology), and mouse anti-VAP-A (Cat# sc-293278, Santa Cruz Biotechnology).

The fluorescent secondary antibodies used in this study were as follows: Cy3 Goat Anti-Mouse IgG (H+L) (Cat# AS008, Abclonal), Cy3 Goat Anti-Rabbit IgG (H+L) (Cat# AS007, Abclonal), FITC Goat Anti-Mouse IgG (H+L) (Cat# AS001, Abclonal), and FITC Goat Anti-Rabbit IgG (H+L) (Cat# AS011, Abclonal).

The following reagents were purchased from the manufacturers as noted: benzyl 2-acetamido-2-deoxy-α-D-galactopyranoside (BAGN, Cat# B4894, Sigma Aldrich), DC-LC3in-D5 (Cat# HY-141882, MedChemExpress), Endo H (Cat# P0702S, New England Biolabs), ER-Tracker™ Green (Cat# E34251, Invitrogen), E64D (Cat# HY-100229, MedChemExpress), Hoechst 33342 (Cat# HY-15559, MedChemExpress), LAMP1 Protein, Human, Recombinant (His Tag) (Cat# 11215-H08H, Sino Biological), LAMP2 Protein, Human, Recombinant (His Tag) (Cat# 13555-H08H, Sino Biological), L-leucyl-L-leucine methyl ester (LLOMe, Cat# L7393, Sigma Aldrich), Lipofectamine™ 3000 Transfection Reagent (Cat# L3000001, Invitrogen), LysoTracker™ Deep Red (Cat# L12492, Invitrogen), Nano-SiO2 (Cat# tlrl-sio-2, Invivogen), Pepstatin A (Cat# HY-P0018, MedChemExpress), Nickle-beads (Cat# 30250, Qiagen, GmbH, Hilden, Germany), PNGase F (Cat# P0704S, New England Biolabs), Proteinase K, from Tritirachium album (Cat# P6556, Sigma Aldrich), RPMI 1640 Medium (Cat# 31800022, Gibco), Olitigaltin (TD139, Cat# HY-19940, MedChemExpress), Tris(2-carboxyethyl) phosphine hydrochloride (Cat# S16054, Shanghai yuanye Bio-Technology Co., Ltd), Thrombin (Cat# S10117, Shanghai yuanye Bio-Technology Co., Ltd), Transferrin Receptor/TFR1/CD71 Protein, Human, Recombinant (ECD, His Tag) (Cat# 11020-H07H, Sino Biological), Trypsin (Cat# S10032, Shanghai yuanye Bio-Technology Co., Ltd), Tunicamycin (TM, Cat# 11089-65-9, Aladdin), Z-VAD-FMK (Cat# HY-16658B, MedChemExpress), 1,6-Hexanediol (1,6-HD, Cat# 240117, Sigma Aldrich), 3-Methyladenine (3-MA, Cat# HY-19312, MedChemExpress), and Protein A/G Magnetic Beads (Cat# HY-K0202, MedChemExpress).

### Bacterial strains

*E. coli* DH5α and BL21 (DE3) strains were obtained from Synbio Technologies (Suzhou, China) and cultured in Luria-Bertani (LB) medium.

### Cell lines and culture

HeLa (Cat# CCL-2) and RAW264.7 (Cat# TIB-71) cell lines were obtained from American Type Culture Collection (ATCC). A549 (Cat# SCSP-503), HT-22 (Cat# GNM47), U2OS (Cat# SCSP-5030), and Hepa 1–6 (Cat# SCSP-512) cell lines were obtained from the Cell Bank/Stem Cell Bank of the Chinese Academy of Sciences. HeLa, A549, HT-22, Hepa 1–6, and RAW264.7 cells were cultured in Dulbecco’s Modified Eagle’s Medium (DMEM, Cat# 12100046, Gibco) supplemented with 10% fetal bovine serum (FBS, Cat# F0193, Sigma Aldrich) and 1% penicillin-streptomycin. U2OS cells were cultured in McCoy’s 5A (Modified) Medium (Cat# 16600082, Gibco) with 10% FBS and 1% penicillin-streptomycin. All cell lines were maintained in a humidified incubator at 37 °C with 5% CO_2_.

### Lysosomal damage models

L-leucyl-L-leucine methyl ester (LLOMe), silica particles, outer membrane vesicles (OMVs), or SARS-CoV-2 ORF3a to induce lysosomal damage. For LLOMe treatment, cells were incubated in SFM containing 0.25, 0.5, 1, 1.5, or 2 mM LLOMe for 30 min. For silica treatment, cells were incubated in SFM containing 1, 2.5, 5, 10, 20, or 40 µg/mL silica for 30 min. For OMV treatment, cells were incubated in SFM containing 120 µg OMV/well (6-well plate) for 2 h. For ORF3a treatment, cells were transfected with the plasmid pCMV3-C-ORF3a-FLAG (Synbio Technologies) for 24 h. After each treatment, cells were rinsed and maintained in SFM for 4 h or as otherwise indicated. Lysosomal damage was assessed, and the culture medium was collected for evaluation of galectin-3 (Gal-3) secretion, as described below.

In some experiments, prior to induction of lysosomal damage, cells were pre-treated with various reagents: E64D+Pepstatin A (both 10 µM, 30 min), Z-VAD-FMK (10 µM, 30 min), 3-methyladenine (3-MA, 5 mM, 1 h), DC-LC3in-D5 (10 µM or 30 µM, 24 h), tunicamycin (TM, 1 µM, 24 h) and benzyl 2-acetamido-2-deoxy-α-D-galactopyranoside (BAGN, 2 mM, 24 h).

### Assessment of protein secretion

Following the lysosomal damage treatments, the culture medium was collected and centrifuged at 10,000 × *g* for 5 min to remove cellular debris. The supernatant was concentrated using a 3 kDa Amicon Ultra filter (Cat# UFC5003, Millipore, USA) and analyzed for Gal-3 and other proteins by Western blotting or enzyme-linked immunosorbent assay (ELISA), according to the manufacturer’s protocols. Human Galectin-3 ELISA Kit (Cat# abs551016, Absin, Shanghai, China) and Mouse Galectin-3 ELISA Kit (Cat# EK0765, Boster, Wuhan, China) were used in this study.

### Assessment of TD139 on Gal-3 secretion

To evaluate the effect of TD139 on Gal-3 secretion, cells were first treated with 50 mM lactose for 2 h, to remove Gal-3 bound to the cell surface. The cells were then pre-treated with 1 µM TD139 for 2 h before induction of lysosomal damage. Gal-3 secretion was subsequently assessed as described.

### Assessment of 1,6-hexanediol (1,6-HD) on Gal-3 secretion

Cells were treated with 1 mM LLOMe for 30 min, and 1% 1,6-HD was added at the 25 min mark to impair phase separation. After washing out LLOMe, cells were incubated with SFM for an additional 4 h. The culture medium was collected and analyzed by Western blotting.

### Assessment of lysosomal damage by LysoTracker assay

Cells were seeded in 24-well plates with round coverslips. Following lysosomal damage, cells were incubated with 200 nM LysoTracker Red dye for 30 min at 37 °C. Cells were then gently rinsed with phosphate-buffered saline (PBS) and fixed in 4% paraformaldehyde (PFA) for 15 min. After additional PBS washes, cells were stained with 200 μg/mL Hoechst 33342 for 10 min and observed and imaged using a LSM880 confocal laser scanning microscope (Carl Zeiss, Oberkochen, Germany) under identical settings.

### Preparation of outer membrane vesicles (OMVs)

OMVs were prepared from *E. coli* BL21 (DE3). Bacteria were grown in 200 mL LB medium until the optical density at 600 nm (OD_600_) reached approximately 0.5. The culture was centrifuged at 10,000 × *g* for 10 min at 4 °C, and the supernatant was filtered through 0.45 μm filters (Millipore) to remove residual cells. The filtrate was ultracentrifuged at 284,000 × *g* for 1.5 h at 4 °C using an Optima MAX-XP Ultracentrifuge (Beckman Coulter, Brea, CA, USA) with an MLA-55 fixed-angle rotor. The resulting pellet containing OMVs was recovered and resuspended in 300 μL sterile PBS. To ensure the absence of bacterial contamination, OMV samples were subjected to agar plating. The size distribution of OMVs was assessed by dynamic light scattering (DLS), and protein concentration was determined using a bicinchoninic acid (BCA) Protein Assay Kit (AR0146, Boster, Wuhan, China) following the manufacturer’s instructions.

### Preparation and analysis of the 100,000 × *g* pellet fraction

HeLa cells grown in 10 cm tissue culture dishes were treated with 1 mM LLOMe as described in the lysosomal damage model section. Following treatment, the culture medium was collected and centrifuged at 10,000 × *g* for 5 min to remove cell debris, followed by ultracentrifugation at 100,000 ×  *g* for 1.5 h at 4 °C (Optima MAX-XP Ultracentrifuge, Beckman Coulter) with the MLA-55 fixed-angle rotor. The resulting pellet fraction was resuspended in PBS and analyzed either by DLS for particle size or by Western blotting for Gal-3 and glycoproteins content.

For mass spectrometry (MS) analysis, 100 mL of culture medium was used to prepare the 100,000 × *g* pellet fraction.

### Pull-down assay with protein A/G magnetic beads

To perform the pull-down assay, 30 μL of protein A/G magnetic beads was pre-incubated with 2 μL of His-tag antibody for 2 h at 4 °C. Then, 100 μg of HeLa cell lysate and the pre-treated beads were incubated with or without 100 μg His-tagged Gal-3 overnight. The beads were subsequently analyzed by silver staining or subjected to MS.

### Pull-down assay with lactose beads

Lactose (Lac)-beads were prepared as previously described^38^. Briefly, 30 μL Lac-beads were incubated in 300 μL PBST containing 50 μg Gal-3 at 4 °C for 2 h. The Lac-beads coated with Gal-3 were then incubated at 4 °C overnight in 200 μg HeLa lysates. Beads were washed three times with PBST, and proteins were detected by Western blotting.

### Binding assay with Ni-NTA beads

Ni-NTA beads were purchased from Qiagen, GmbH (Hilden, Germany). All proteins used in the binding assay were dissolved in PBST. Briefly, 25 μL Ni-NTA beads were incubated with 7 nM His-LAMP1, followed by 0.5 μM Gal-3 in 300 μL PBST at 4 °C for 2 h. The beads were washed twice with PBST and then incubated in 300 μL PBST containing 0.5 μM mCherry-ALG-2 with or without 0.5 μM Gal-3 at 4 °C for 2 h. After three washes with PBST, bound proteins were detected by Western blotting (Fig. 5F).

### Mass spectrometry (MS) analysis of the 100,000 × *g* pellet fraction and pulldown proteins

MS was performed according to our previously published protocol^79^. Briefly, proteins from the 100,000 × *g* pellet fraction and pulldown samples were treated sequentially with 10 mM dithiothreitol (DTT), 20 mM iodoacetamide (IAA), and 2 μg trypsin. The resulting peptides were desalted and spin-dried using a C18 column. Peptides were re-suspended in 0.1% formic acid prior to separation, and they were analyzed using an EASY-nLC 1200 liquid chromatographic system coupled online to a Q-Exactive mass spectrometer (Thermo Fisher Scientific, San Jose, CA, USA) equipped with a nano-electrospray ion source. For each injection, 3 μL of peptide sample was loaded onto an Acclaim PepMap^TM^ 100 trap column (C18, 100 μm × 2 cm, 5  μm, 100 Å) at a flow rate of 5 μL/min and separated on an Acclaim PepMap^TM^ RSLC analytical column (C18, 50 μm × 15 cm, 2 μm, 100 Å) at a flow rate of 300 nL/min. Buffer A consisted of 0.1% formic acid, and Buffer B was 80% acetonitrile with 0.1% formic acid. The linear gradient was programmed as follows: 5–30% Buffer B for 80 min, followed by 40% Buffer B for 16 min, then maintained at 100% Buffer B for 8 min. The electrospray voltage was 3 kV. The mass spectrometer was operated in data-dependent acquisition (DDA) mode. Full MS scans were acquired from m/z 350 to 2000 at a resolution of 70,000 (measured at *m/z* 200), an automatic gain control (AGC) target of 3e^6^, and a maximum injection time of 50 ms. The top 15 precursor ions (charge +2 to +5) were fragmented via higher-energy collisional dissociation (HCD) with a normalized collision energy of 28%. Data were collected at a resolution of 17,500, an AGC target of 1e^5^, and a maximum injection time of 45 ms. Dynamic exclusion was done with an exclusion duration of 30 s and a mass window of 10 ppm. Raw MS files were processed using Proteome Discoverer 2.2 and searched against the UniProt human proteome database. Database searches were performed with trypsin/P specificity, allowing a maximum of two missed cleavages. Cysteine carbamidomethylation was a fixed modification, whereas methionine oxidation and protein N-terminal acetylation were variable modifications. Mass tolerances were set to 10 ppm for precursor ions and 0.02 Da for fragment ions. Peptide and protein identifications were filtered at 1% FDR using the target-decoy strategy, with a threshold requiring at least one unique peptide per identified protein.

### Particle size assessment by dynamic light scattering (DLS)

The diameters of OMVs and the 100,000 × *g* pellet fraction were determined using a NanoBrook Omni instrument (Brookhaven, NY, USA). All measurements were performed at a scattering angle of 90° and repeated three times at 25 °C for OMVs and at 37 °C for the pellet fraction.

### Assessment of sensitivity to trypsin/proteinase K

To determine whether secreted Gal-3 was enclosed within membrane structures, the culture medium obtained from each lysosomal damage model was treated with or without 1% Triton X-100 for 30 min at 37 °C. Subsequently, trypsin or proteinase K was added at a final concentration of 200 or 50 μg/mL, respectively. After incubation at 37 °C for 30 min, the digested samples were denatured in sample loading buffer by boiling and analyzed by Western blotting.

### Protein expression and purification

Recombinant human Gal-3 and its variants/mutants were prepared as previously described^80,81^. Briefly, the *Gal-3* gene was cloned into the pET22b vector to produce an untagged protein. *Gal-3* truncates (CRD, NT, Δ68) and various proline mutants were cloned into the pET28a vector to generate His-tagged proteins. Constructs for *GFP-Gal-3*, *GFP-Gal-3(R186S)*, and *GFP-CRD* were also cloned into the pET28a vector to produce His-tagged GFP fusion proteins. All constructs were synthesized by Synbio Technologies (Suzhou, China).

*E. coli* BL21 (DE3) cells, transformed with the respective plasmids, were grown in LB medium containing 100 μg/mL kanamycin at 37 °C until reaching an OD_600_ of 0.6–0.8, and protein expression was induced by adding 0.5 mM isopropyl-β-d-thiogalactopyranoside (IPTG) at 25 °C overnight. Cells were harvested and lysed by high-pressure homogenization in PBS (pH 7.4). The lysates were centrifuged at 14,300 × *g* for 60 min at 4 °C. Target proteins in the supernatant were purified using nickel beads (for His-tagged proteins) or lactose-Sepharose CL-6B beads (for untagged proteins)^80,81^. In some cases, the His-tag was cleaved with thrombin^80,81^.

ALG-2 and its truncates (Δ26, Δ80, and ΔGF122) as well as the Y180A mutant were prepared as mCherry fusion proteins. The *mCherry-ALG-2* gene and its variants were cloned into the pET28a plasmid (Synbio Technologies, Suzhou, China) and transformed into *E. coli* BL21 (DE3), and expressed as described above. Cells were lysed in a buffer containing 50 mM Tris, pH 7.4, 150 mM NaCl, and 0.2 mM Tris(2-carboxyethyl) phosphine hydrochloride. The target proteins were purified using nickel beads^80,81^.

For the construction of GFP- or mCherry-fusion proteins, the *GFP* or *mCherry* gene was fused to the 5′ end of the target gene (Synbio Technologies, Suzhou, China).

Purified proteins were aliquoted and stored at −80 °C. Protein purity was assessed by SDS-PAGE (Supplementary Fig. 12).

Detailed information is presented in Supplementary Data 3.

### Immunofluorescence

Cells grown on round coverslips were subjected to plasmid transfection, siRNA transfection, or drug treatment. Cells were fixed with 4% PFA for 15 min at 25 °C, rinsed three times with cold PBS, permeabilized with 1% Triton X-100 for 8 min, and rinsed three times with cold PBS. After blocking for 1 h at 25 °C in blocking buffer (PBS containing 5% fetal calf serum [FCS] and 1% bovine serum albumin [BSA]), cells were incubated overnight at 4 °C with primary antibodies diluted in blocking buffer: anti-ERGIC53 (1:100), anti-Gal-1 (1:50), anti-Gal-3 (1:400), anti-Gal-8 (1:100), anti-Rab7a (1:50), anti-LAMP1 (1:100), anti-LAMP2 (MAB6228, R&D Systems) (1:20), and anti-CD71 (1:100). After washing three times with cold PBS, cells were incubated with fluorescent secondary antibodies diluted 1:100 in blocking buffer for 45 min at 25 °C. Secondary antibodies used included: fluorescein Isothiocyanate (FITC) Goat Anti-Rabbit IgG (H+L), FITC Goat Anti-Mouse IgG (H+L), Cy3 Goat Anti-Rabbit IgG (H+L), and Cy3 Goat Anti-Mouse IgG (H+L). Images were captured using a LSM880 confocal laser scanning microscope (Carl Zeiss, Oberkochen, Germany) with a 20× objective (Supplementary Fig. 1), a 63× oil objective (Figs. 3L, M, and 4J, K), or a 100× oil objective (Figs. 1, 3, 4, and 7). For Supplementary Fig. 10, imaging was performed using the Operetta CLS high-content imaging system (PerkinElmer, Massachusetts, USA) with a 63× water objective. Quantification was carried out using the ImageJ software.

### Enzymatic deglycosylation assay

LAMP1 was enzymatically deglycosylated using PNGase F or Endo H. For both treatments, 4 µg of LAMP1 was first denatured by heating at 100 °C for 10 min. The denatured sample was then incubated with 1 U of either PNGase F or Endo H at 37 °C for 1 h or a series of time points (1, 2, 4, 8, and 24 h) in a final reaction volume of 20 µL. All other conditions followed the manufacturer’s instructions.

### Liquid-liquid phase separation (LLPS) assay

The LLPS assay was performed as previously described^38^. Briefly, various combinations of glycoproteins (LAMP1, LAMP2, or CD71), Gal-3 variants, ALG-2 variants were mixed as indicated and incubated at 37 °C for 5 min to allow phase separation (droplet formation). The degree of separation was assessed using confocal microscopy and differential interference contrast (DIC) imaging (Figs. 3A and 4J), by measuring turbidity at 400 nm (Fig. 3B, G, H), by analyzing droplet composition via Western blotting (Figs. 4H, I, and 6C), and/or by detecting droplet dynamics by FRAP (Fig. 4K, L).

### FRAP analysis

HeLa cells transfected with mCherry-Gal-3 or mCherry-CRD plasmids were treated with 1 mM LLOMe for 30 min. FRAP analysis was performed using a LSM880 confocal laser scanning microscope (Carl Zeiss, Oberkochen, Germany) equipped with a Plan-Apochromat 63× oil immersion objective at 25 °C. Fluorescence within mCherry-Gal-3 or mCherry-CRD puncta was photobleached using a 546 nm laser at 20–60% intensity, and recovery of fluorescence intensity was recorded every 0.7 s.

For droplet FRAP, mixtures containing LAMP1 (3 μM), Gal-3 (35 μM), GFP-Gal-3 (12.5 μM), and mCherry-ALG-2 (10 μM) were incubated to allow droplet formation. Droplets were analyzed using the same microscope with a 100× oil immersion objective. GFP-Gal-3 droplets were photobleached using a 488 nm laser at 20–60% intensity, and recovery of fluorescence intensity was recorded every 0.7 s.

For quantitative analysis, fluorescence intensity prior to photobleaching was normalized to 1. Results are expressed as the mean ± SEM. Image intensity was quantified using the mean region of interest (ROI) and further analyzed using GraphPad Prism 6 software.

### Plasmid transfection

The pcDNA3.1 plasmid constructs containing *GFP-Gal-3*, *GFP-CRD*, *GFP-Gal-3(R186S)*, *mCherry-Gal-3*, *mCherry-CRD*, *mCherry-Gal-3(R186S)*, and *mCherry-ALG-2* genes were synthesized by Synbio Technologies (Suzhou, China). Plasmids were transfected into HeLa or U2OS cells using Lipofectamine™ 3000 according to the manufacturer’s protocol. After transfection (24 h), cells were treated with LLOMe as described in the Lysosomal damage model section. Cells were then either analyzed by FRAP or stained with ER-Tracker™ Green dye or fixed with PFA for immunofluorescence analyses. In some experiments, cells were rinsed and maintained in SFM for 4 h, after which the culture medium was collected for evaluation of Gal-3, CRD, or Gal-3(R186S) secretion.

Detailed information can be found in Supplementary Data 3.

### Gene knockdown with siRNA

siRNA targeting the following proteins were used: Gal-3, Gal-1, SNAP23, SNAP29, STX3, STX4, Rab7a, GSDME, TMED10, TRIM16, ULK1, Beclin1, ATG16L1, magnesium transporter protein 1 (MGAT1), LAMP1, LAMP2, CD71, ALIX, and ALG-2. siRNAs were synthesized by JTS Scientific (Nanjing, China), dissolved in diethyl pyrocarbonate-treated water (DEPC-H_2_O) at 20 µM, and stored at −20 °C. Transfections were performed using Lipofectamine™ 3000 according to the manufacturer’s instructions. After 24 h, HeLa or A549 cells were treated with LLOMe or silica particles, as described in the Lysosomal damage model section. Cells were then stained with ER-Tracker™ Green dye or fixed with PFA for immunofluorescence analysis. In some cases, cells were rinsed and maintained in SFM for 4 h. The culture medium was collected for protein secretion analysis, and the cells were lysed with lysis buffer (100 mM Tris-HCl, pH 7.5, 300 mM NaCl, and 1% Triton X-100) for assessing knockdown efficiency.

Detailed information can be found in Supplementary Data 4.

### Biolayer interferometry (BLI) assay

The interactions between Gal-3 (or its variants) and ALG-2 (or its variants) were determined using an Octet RED96 instrument (ForteBio, CA, USA) and Ni-NTA sensors as described previously^38^. All proteins were dissolved or diluted in PBST. In this study, Gal-3 variants were loaded onto sensors either directly or indirectly.

The main steps in the direct method: ① loading Gal-3 variants: sensors were incubated in wells containing 100 μg/mL His-tagged Gal-3 (or its variant). ② Association: sensors were incubated in wells containing ALG-2 (0–2000 nM). ③ Dissociation: sensors were transferred back to PBST (without ALG-2).

The main steps in the indirect method: ①-i immobilizing LAMP1: sensors were incubated in wells containing 10 μg/mL His-tagged LAMP1. ①-ii loading Gal-3 variants: sensors were incubated in wells containing untagged Gal-3 (2 μM), Gal-3 CRD (2 μM), or Gal-3Δ68 (10 μM). ② Association: sensors were transferred to wells containing the same Gal-3 variant as in step ①-ii, plus ALG-2 (0–2000 nM) or ALG-2 mutants/truncates (2000 nM). ③ Dissociation: Sensors were transferred to wells containing the same Gal-3 variant as in step ①-ii (without ALG-2).

The running programs: loading 120 s, baseline 60 s, association 120 s, and dissociation 120 s. The entire process was recorded as a sensorgram, with the *x*-axis representing the time course and the *y*-axis representing the binding response of ALG-2 to the sensor. For clarity, only partial sensorgrams showing the association and dissociation phases were shown. Data were analyzed using ForteBio Data Analysis Software 7.0, which provided *K*_D_ (equilibrium dissociation constant) values based on a 1:1 binding model.

### Western blotting

Cells and/or culture medium from lysosomal damage models were boiled in sample loading buffer and subjected to 12% SDS-PAGE and transferred to polyvinylidene difluoride (PVDF) membranes, which were blocked with blocking buffer (5% milk dissolved in PBST) for 1 h at 25 °C. The membranes were then incubated with primary antibodies overnight at appropriate dilutions. After incubation with the secondary antibody for 1 h at 25 °C, membranes were processed for scanning using the automatic chemiluminescence imaging analysis system (Tanon 5200, Shanghai, China).

### LDH assay

The release of LDH following lysosomal damage was measured using the LDH Assay Kit (Cat# HY-K1090, MedChemExpress). Briefly, the assay was performed in a 96-well plate with the following groups: experimental group, high control group, high control blank, low control group, and background blank. 50 μL supernatant was combined with 50 μL of the working solution and mixed gently. The mixtures were then incubated at room temperature in the dark for 30 min. After incubation, 50 μL of stop solution was added, and the absorbance was measured at 490 nm. The percentage of LDH release was calculated using the following equation:

LDH release (%) = [(*X* − *Z*)/(*Y* − *Z*)] × 100%

*X* = OD (experimental group) − OD (background blank)

*Y* = OD (high control group) − OD (high control blank)

*Z* = OD (low control group) − OD (background blank)

### MTT assay

Following lysosomal damage, cell viability was assessed using the MTT [3-(4,5)-dimethylthiahiazo (-z-y1)-3,5-di-phenytetrazoliumromide] assay as previously described^82^.

### Statistics and reproducibility

Statistical analyses were performed using GraphPad Prism v.8.4 software. All data are presented as the mean ± SEM. Differences between the two groups were assessed with a two-tailed Student’s *t* test. Differences are indicated as follows: ns for not significant, *: *p* < 0.05, **: *p* < 0.01, ***: *p* < 0.001, and ****: *p* < 0.0001. All experiments were independently repeated at least three times to ensure reproducibility. The experiments in Figs. 1A–C, M, O, 3A, C, D, E, L, 4A, B, E, H–J, 5F, 6C, 7A, D, F, I, and Supplementary Figs. 4A, 10A, C, 11B were independently repeated at least three times with similar results.

### Reporting summary

Further information on research design is available in the Nature Portfolio Reporting Summary linked to this article.

## Supplementary information

Supplementary Information

Description of Additional Supplementary Files

Supplementary Data 1

Supplementary Data 2

Supplementary Data 3

Supplementary Data 4

Reporting Summary

Transparent Peer Review File

## Source data

Source Data

## Data Availability

The mass spectrometry proteomics data of the 100,000 × *g* pellet fraction and pulldown proteins generated in this study are deposited to the ProteomeXchange database via the iProX^83,84^ partner repository with the dataset identifiers PXD080292 and PXD080293. Source data are provided with this paper.
