# Supplementary material for: Glycoprotein-induced phase separation drives unconventional secretion of galectin-3
Source: Nat Commun. 2026 Aug 6;17:9484. doi: 10.1038/s41467-026-76321-w (PMC13542199; doi:10.1038/s41467-026-76321-w)
Supplement: Supplementary file 1 [file 41467_2026_76321_MOESM1_ESM.pdf]

Supplementary Information

Glycoprotein-induced phase separation drives unconventional secretion of galectin-3

Zihan Zhao[1,#], Zhen He[1,#], Hongming Gu[1], Andong Zhou[1], Yibing Wang[1], Zhaoyi Liang[1], Jie Geng[1], Xuejiao Xu[1], Menghui Wang[1], Chunyao Li[1], Yuying Fan[1], Hairong Cheng[1], Lin Sun[1], Kevin H. Mayo[2], Avraham Raz[3,*], Guihua Tai[1,*], Yifa Zhou[1,*]

[1]Engineering Research Center of Glycoconjugates Ministry of Education, Jilin Provincial Key Laboratory of Chemistry and Biology of Changbai Mountain Natural Drugs, School of Life Sciences, Northeast Normal University, Changchun 130024, China.
[2]Department of Biochemistry, Molecular Biology and Biophysics, University of Minnesota, Minneapolis, MN 55455, USA.
[3]Department of Oncology, Karmanos Cancer Institute, Wayne State University, School of Medicine Detroit, MI 48201, USA.
[#]These authors contributed equally: Zihan Zhao, Zhen He

*Correspondence: araz@med.wayne.edu, taigh477@nenu.edu.cn, zhouyf383@nenu.edu.cn

This document contains

Supplementary Figures 1–12

Supplementary Figures

Supplementary Figure 1

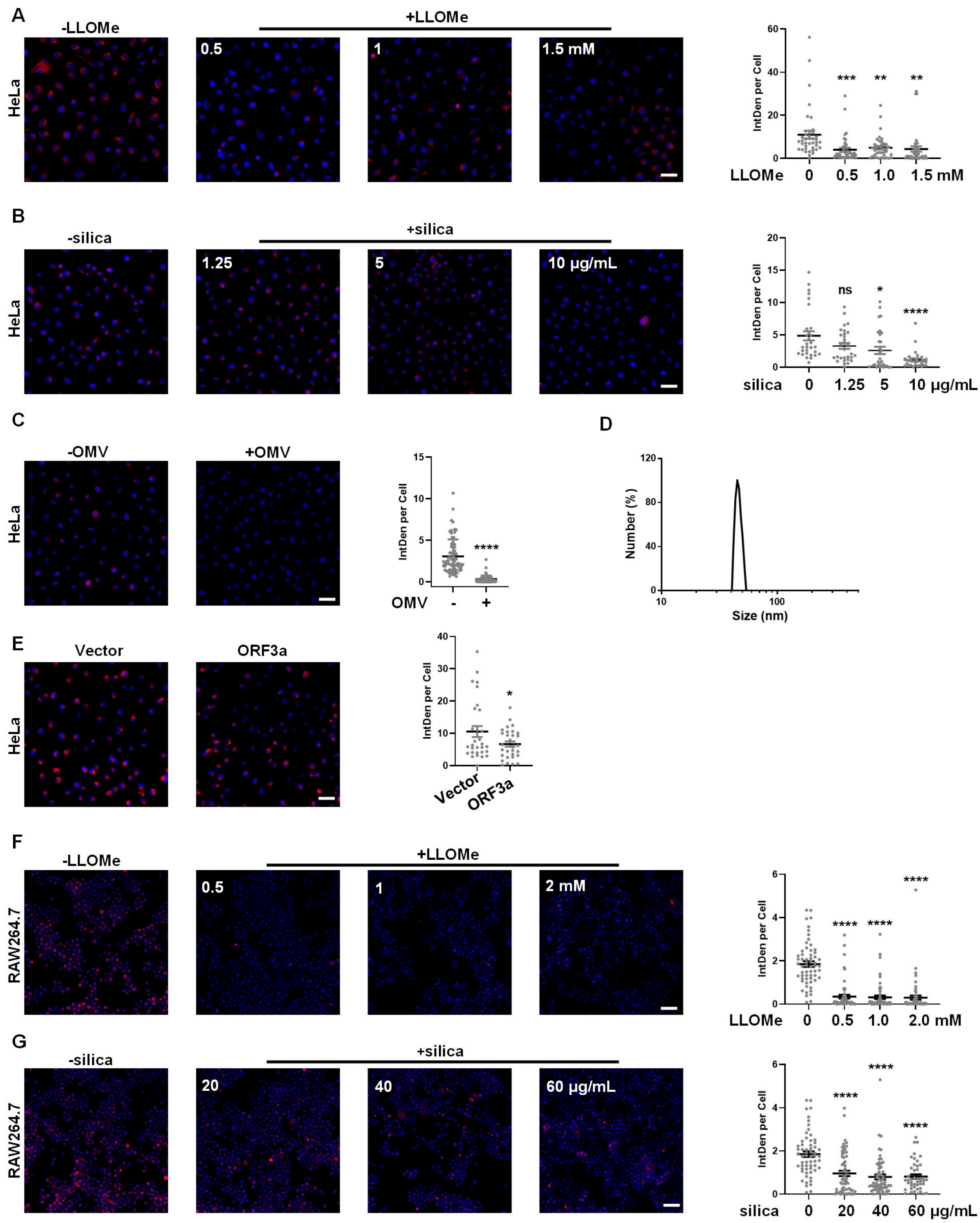

Supplementary Figure 1. Construction of lysosomal damage models.

(**A**) L-Leucyl-L-leucine methyl ester (LLOMe)-induced lysosomal damage in HeLa cells. Cells were treated with 0.5, 1, or 1.5 mM LLOMe for 30 min. Lysosomal integrity was assessed using LysoTracker. $n = 40$ fields from three independent experiments. Representative images are shown in the left panel; quantification is shown in the right panel.

(**B**) Silica-induced lysosomal damage in HeLa cells. Cells were treated with 1.25, 5, or 10 μg/mL silica for 30 min. Lysosomal status was assessed using LysoTracker. $n = 30$ fields from three independent experiments. Representative images are shown in the left panel; quantification is shown in the right panel.

(**C**) Outer membrane vesicle (OMV)-induced lysosomal damage in HeLa cells. Cells were treated with 120 μg OMVs for 2 h. Lysosomal integrity was assessed using LysoTracker. $n = 70$ fields from three independent experiments. Representative images are shown in the left panel; quantification is shown in the right panel.

(**D**) Particle size distribution of OMVs used in (**C**), measured by dynamic light scattering (DLS).

(**E**) SARS-CoV-2 ORF3a-induced lysosomal damage in HeLa cells. Cells were transfected with the pCMV3-C-ORF3a-FLAG plasmid for 24 h. Lysosomal integrity was assessed using LysoTracker. $n = 30$ fields from three independent experiments. Representative images are shown in the left panel; quantification is shown in the right panel.

(**F**) LLOMe-induced lysosomal damage in RAW264.7 cells. Cells were treated with 0.5, 1, or 2 mM LLOMe for 30 min. Lysosomal integrity was assessed using LysoTracker. $n = 60$ fields from three independent experiments. Representative images are shown in the left panel; quantification is shown in the right panel.

(**G**) Silica-induced lysosomal damage in RAW264.7 cells. Cells were treated with 20, 40, or 60 μg/mL silica for 30 min. Lysosomal integrity was assessed using LysoTracker. $n = 45$ fields from three independent experiments. Representative images are shown in the left panel; quantification is shown in the right panel.

Data are shown as the mean ± SEM. Statistical significance was determined using two-tailed Student's *t*-test. $^{*}p < 0.05$, $^{**}p < 0.01$, $^{***}p < 0.001$, $^{****}p < 0.0001$; ns, not significant. Scale bars, 10 μm.

Supplementary Figure 2

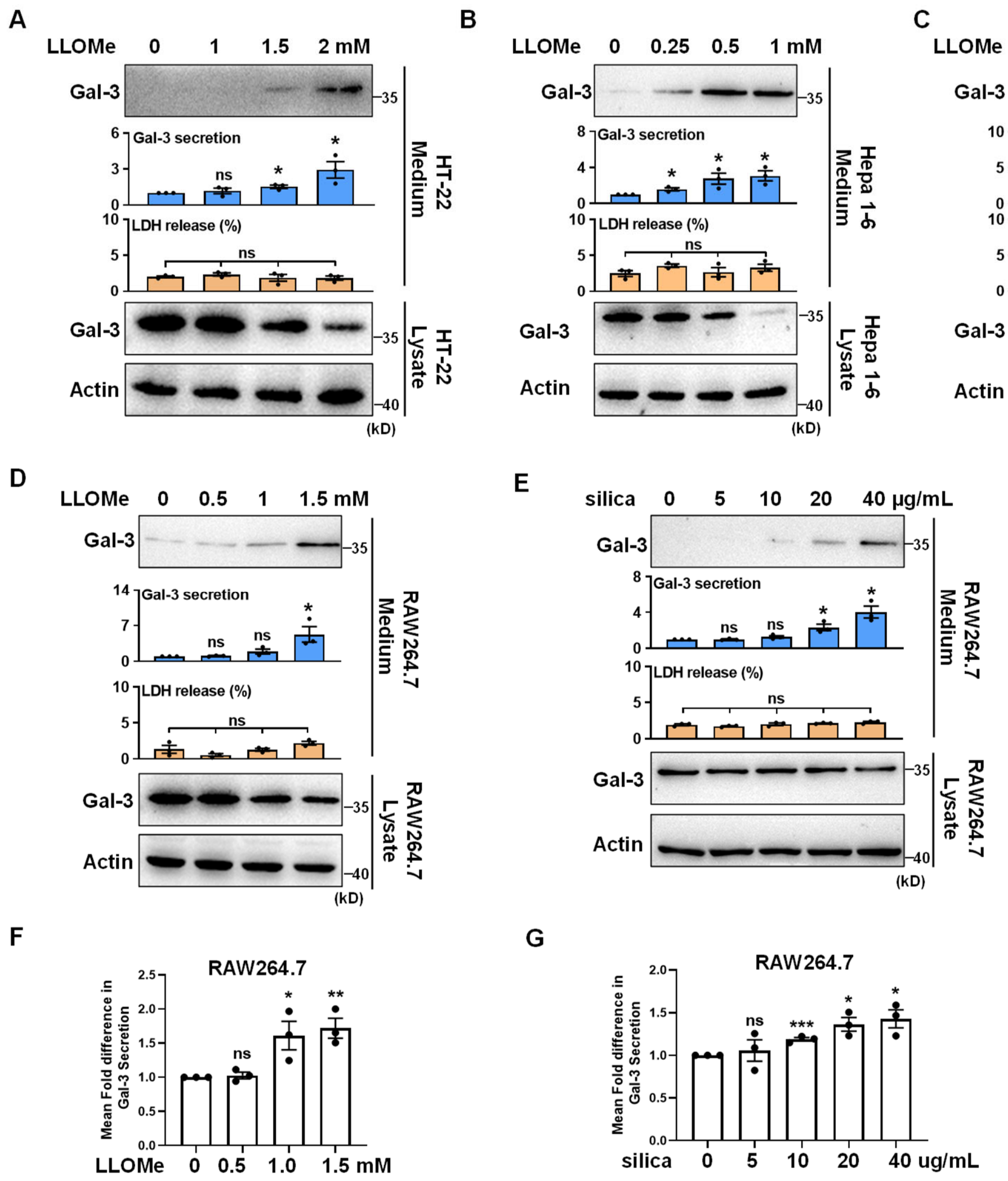

Supplementary Figure 2. Galectin-3 (Gal-3) secretion in additional models of lysosomal damage.

(**A**–**D**) Western blotting of Gal-3 secretion in various cell lines treated with LLOMe: HT-22 (**A**), Hepa 1-6 (**B**), A549 (**C**), and RAW264.7 (**D**). Cells were treated with different concentrations of LLOMe for 30 min. Following treatment, cells were rinsed and incubated in serum-free medium (SFM) for 4 h. The culture medium and cell lysates were collected and analyzed. Bars indicate Gal-3 secretion and LDH release. *n* = 3 independent experiments.

(E) Western blotting of Gal-3 secretion in silica-treated RAW264.7 cells. Cells were treated with varying concentrations of silica for 30 min. After treatment, cells were rinsed and incubated in SFM for 4 h. The culture medium and cell lysates were analyzed. Bars indicate Gal-3 secretion and LDH release. *n* = 3 independent experiments.

(**F**) Enzyme-linked immunosorbent assay (ELISA) analysis of Gal-3 secretion in LLOMe-treated RAW264.7 cells. Treatment was performed as described in (**D**). *n* = 3 independent experiments.

(**G**) ELISA analysis of Gal-3 secretion in silica-treated RAW264.7 cells. Treatment was performed as described in (**E**). *n* = 3 independent experiments.

Data are shown as the mean ± SEM. Statistical significance was determined using two-tailed Student's *t*-test. *$p < 0.05$, **$p < 0.01$, ***$p < 0.001$; ns, not significant.

Supplementary Figure 3

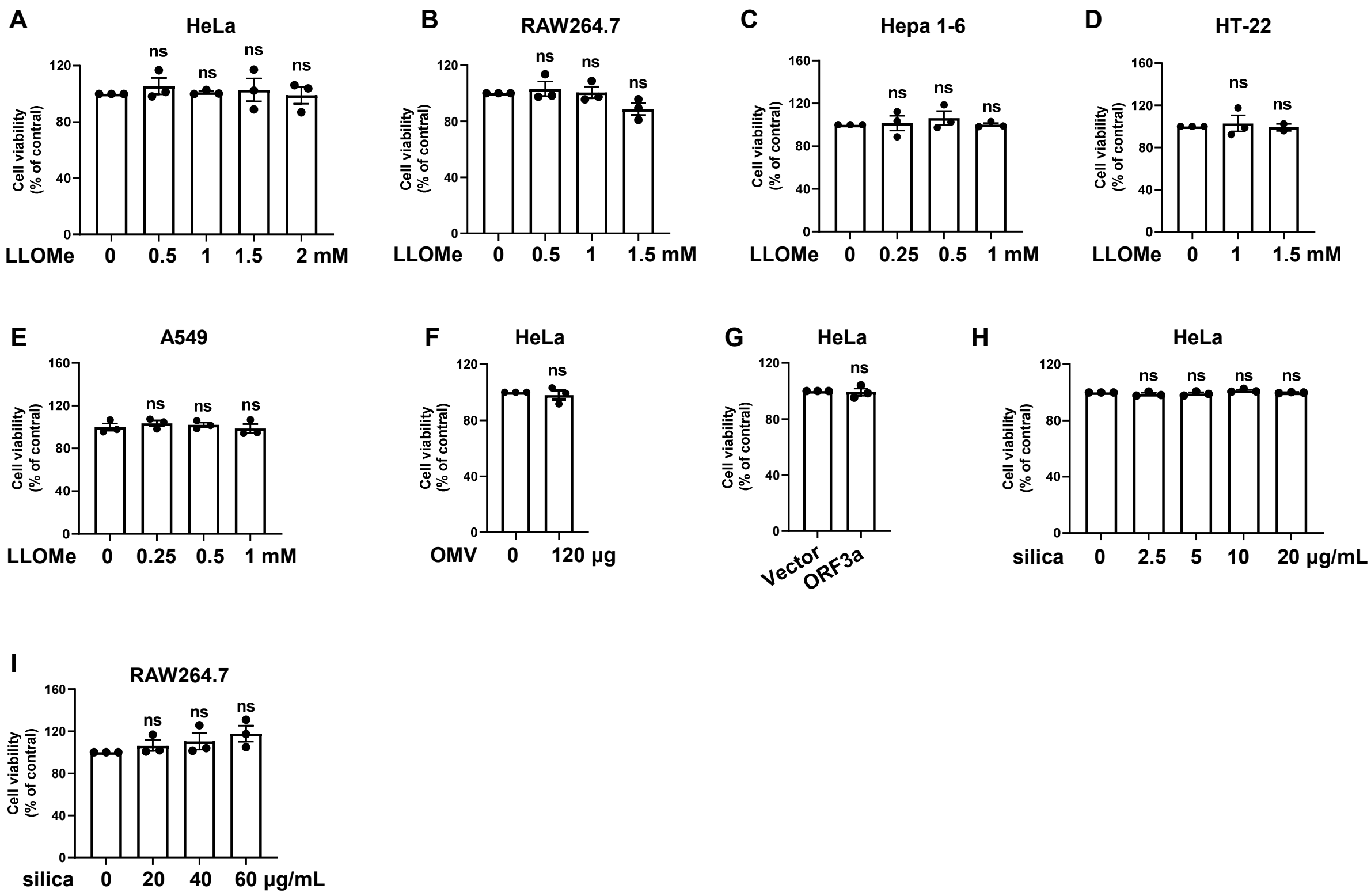

Supplementary Figure 3. Cell viability in lysosomal damage models.

Cell viability was assessed in the following models using the MTT [3-(4,5-dimethylthiazol-2-yl)-2,5-diphenyltetrazolium bromide] assay: LLOMe-treated HeLa (**A**), RAW264.7 (**B**), Hepa 1-6 (**C**), HT-22 (**D**), and A549 (**E**) cells; OMV-treated HeLa cells (**F**); ORF3a-overexpressing HeLa cells (**G**); and silica-treated HeLa (**H**) and RAW264.7 (**I**) cells. Treatments were performed as described in Supplementary Figure 1.

n = 3 independent experiments. Data are shown as the mean ± SEM. Statistical analysis was performed using two-tailed Student's *t*-test. ns, not significant.

Supplementary Figure 4

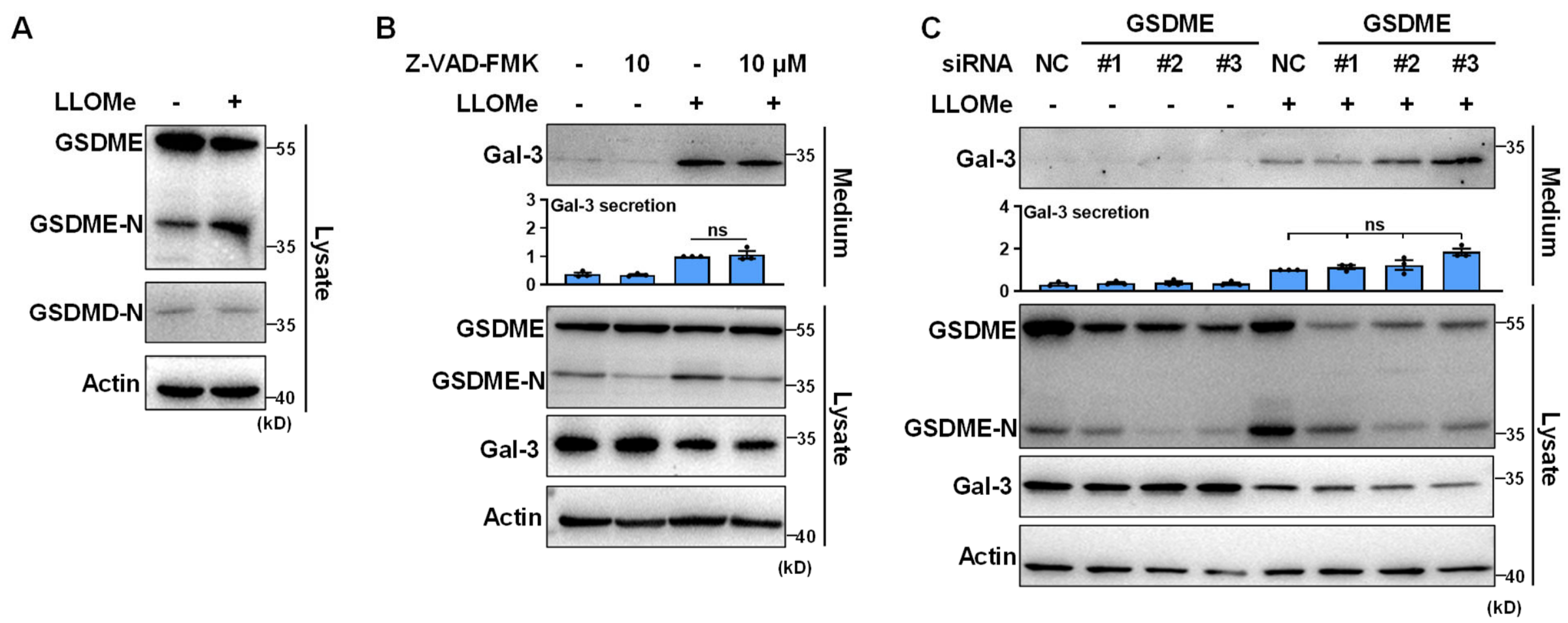

Supplementary Figure 4. Gal-3 secretion is independent of pyroptosis.

HeLa cells, with or without pretreatment as indicated in each panel, were treated with 1 mM LLOMe for 30 min, followed by a 4 h incubation in SFM after LLOMe washout. Gal-3 in the culture medium and other proteins in cell lysates were analyzed by western blotting.

(**A**) Expression of pyroptosis-related proteins in the lysosomal damage model.

(**B**) Effect of pretreatment with the pan-caspase inhibitor Z-VAD-FMK on Gal-3 secretion.

(**C**) Effect of small interfering RNA (siRNA)-mediated knockdown of gasdermin E (GSDME) on Gal-3 secretion.

Bars indicate Gal-3 secretion. Data are shown as the mean ± SEM. Statistical significance was determined using two-tailed Student's *t*-test. ns, not significant. n = 3 independent experiments.

Supplementary Figure 5

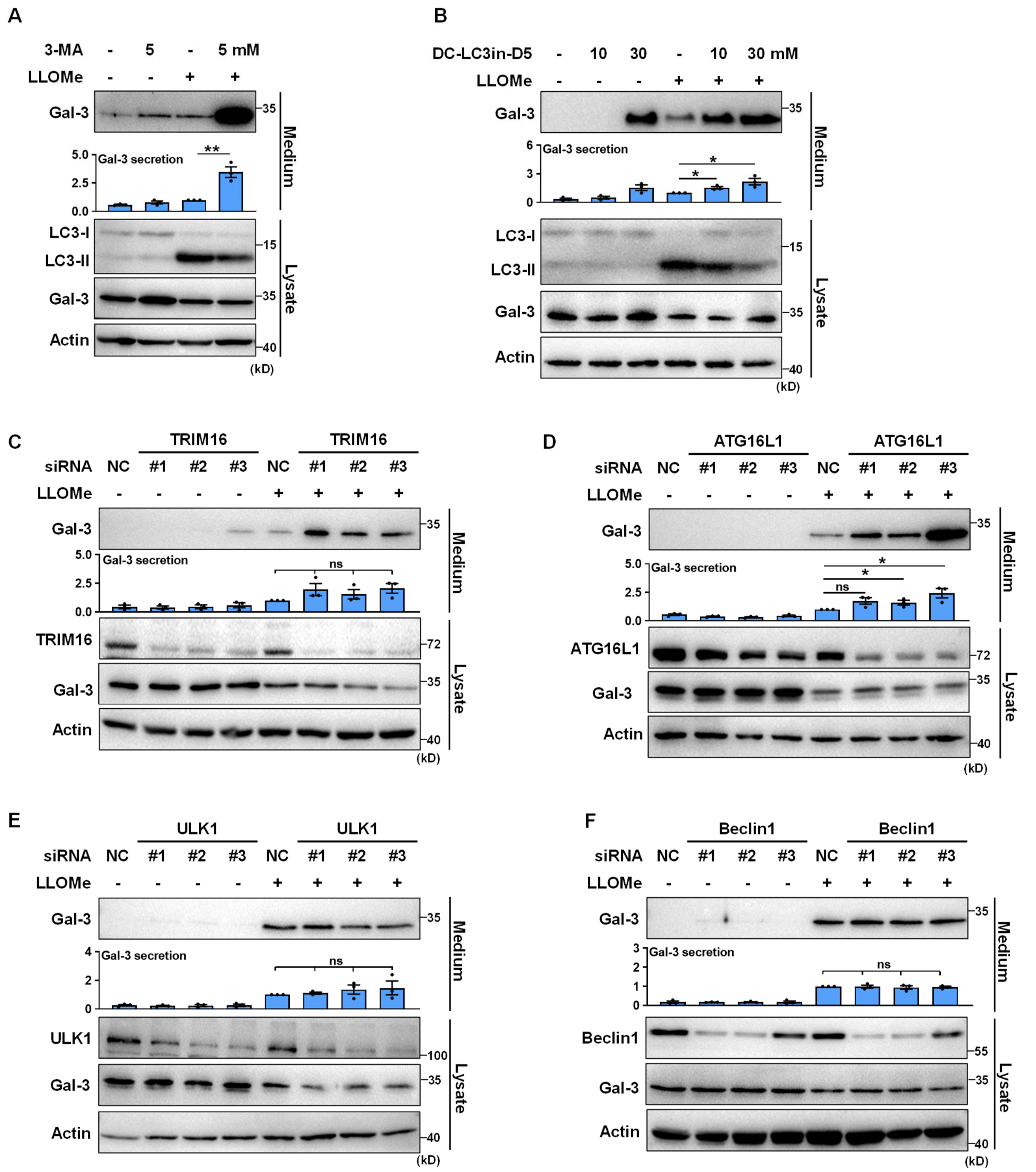

Supplementary Figure 5. Gal-3 secretion is independent of secretory autophagy.

HeLa cells, with or without the indicated pretreatments, were treated with 1 mM LLOMe for 30 min, followed by a 4 h incubation in SFM after LLOMe washout. Gal-3 levels in the culture medium and protein expression in cell lysates were analyzed by western blotting.

(**A** and **B**) Effect of autophagy inhibitors on Gal-3 secretion: 3-methyladenine (3-MA) (**A**) and DC-LC3in-D5 (**B**).

(**C**–**F**) Effect of siRNA-mediated knockdown of autophagy-related proteins: TRIM16 (**C**), ATG16L1 (**D**), ULK1 (**E**), and Beclin1 (**F**).

Bars indicate Gal-3 secretion. Data are shown as the mean ± SEM. Statistical significance was determined using two-tailed Student's *t*-test. *$p < 0.05$, **$p < 0.01$; ns, not significant. $n = 3$ independent experiments.

Supplementary Figure 6

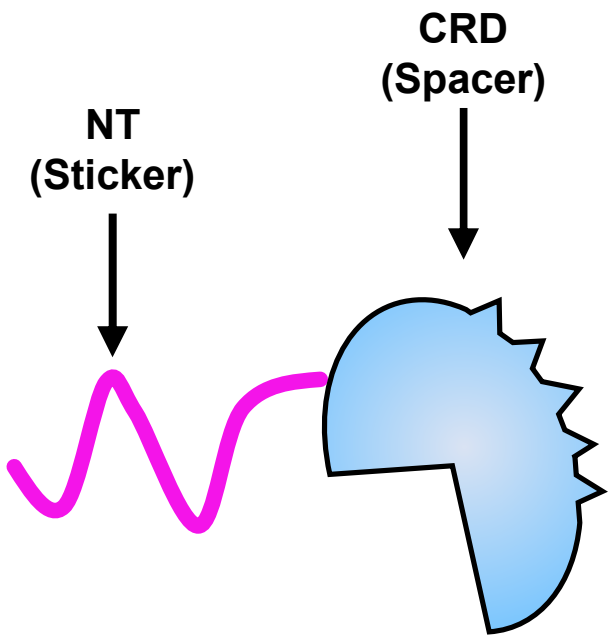

Supplementary Figure 6. Sticker-spacer model of Gal-3.

Supplementary Figure 7

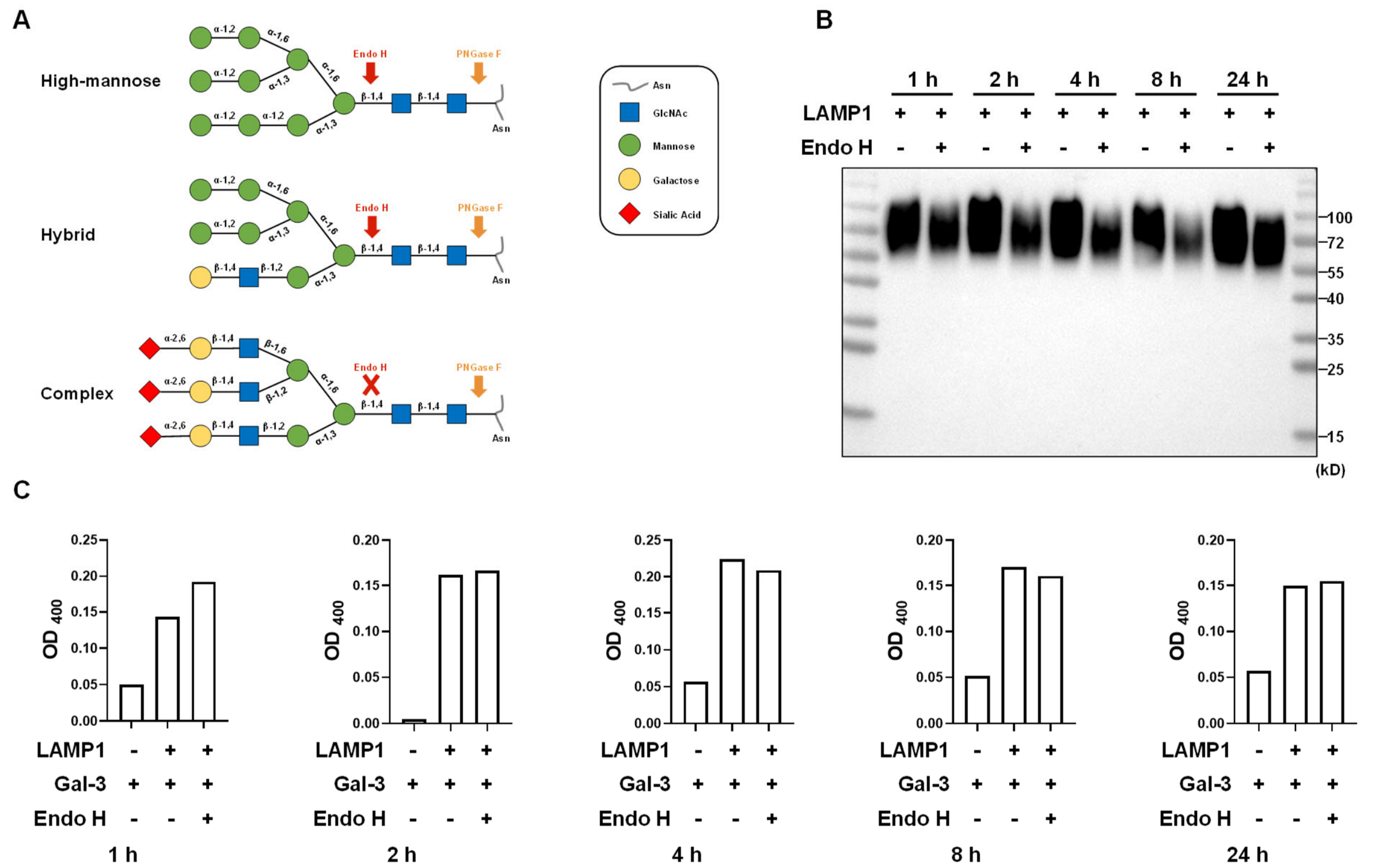

Supplementary Figure 7. Complex-type N-glycans on glycoproteins drive Gal-3 phase separation.

(**A**) Schematic illustration of cleavage of N-glycans by Endo H and PNGase F.

(**B**) Treatment of LAMP1 with Endo H. 2 μM pre-denatured LAMP1 was treated with or without 1000 U Endo H for 1 h, 2 h, 4 h, 8 h, and 24 h. Enzymatic products were analyzed by western blotting.

(**C**) Turbidity analysis of LLPS induced by 5 μM Gal-3 and 25 nM LAMP1 or Endo H-treated LAMP1.

Supplementary Figure 8

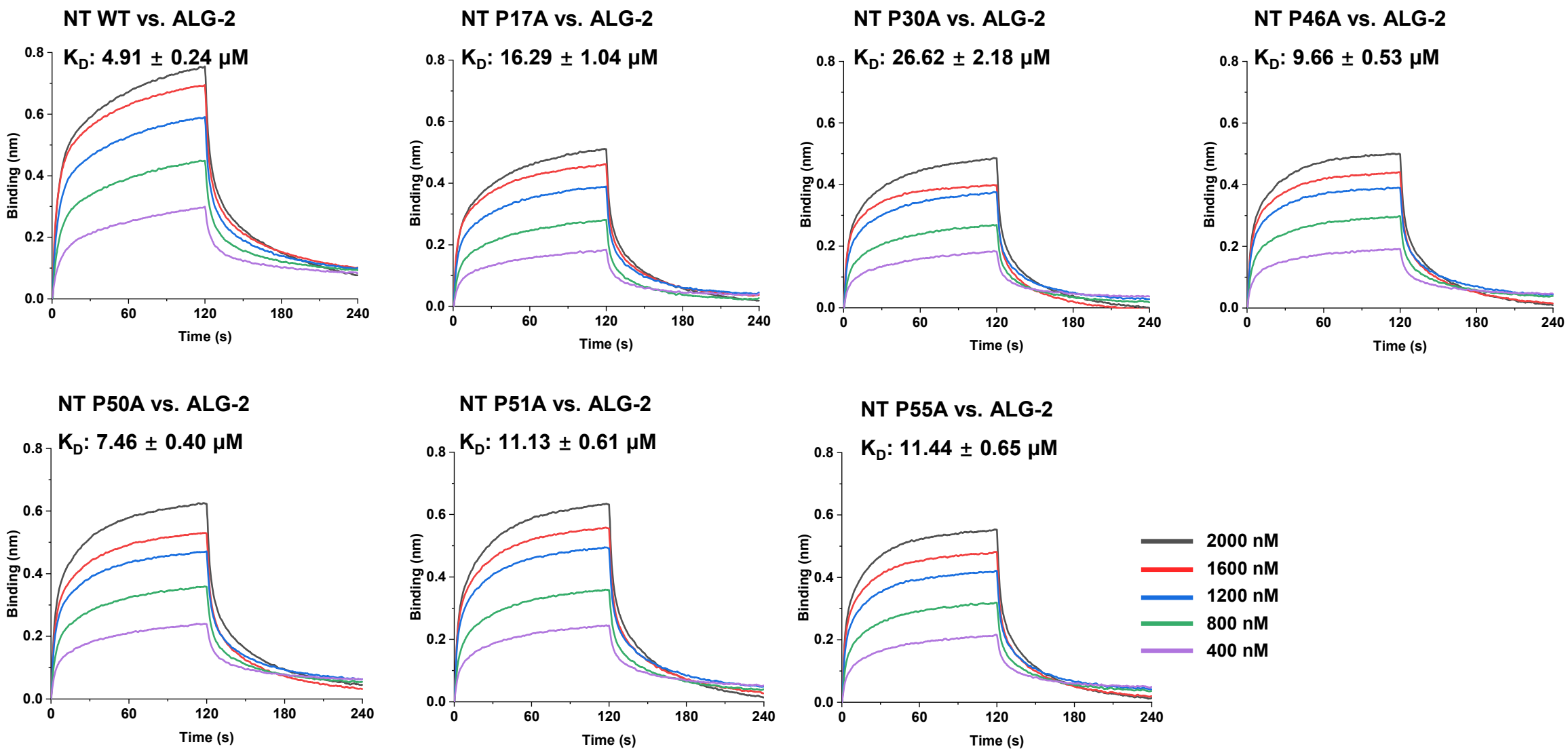

Supplementary Figure 8. BLI analysis of interactions between ALG-2 and proline-mutant NT (P17A, P30A, P46A, P50A, P51A, P55A). Equivalent amounts of 100 μg/mL WT or mutant NT (P17A, P30A, P46A, P50A, P51A, P55A) were immobilized on Ni-NTA sensors via His-tags and exposed to increasing concentrations of ALG-2 (0, 400, 800, 1200, 1600, 2000 nM).

Supplementary Figure 9

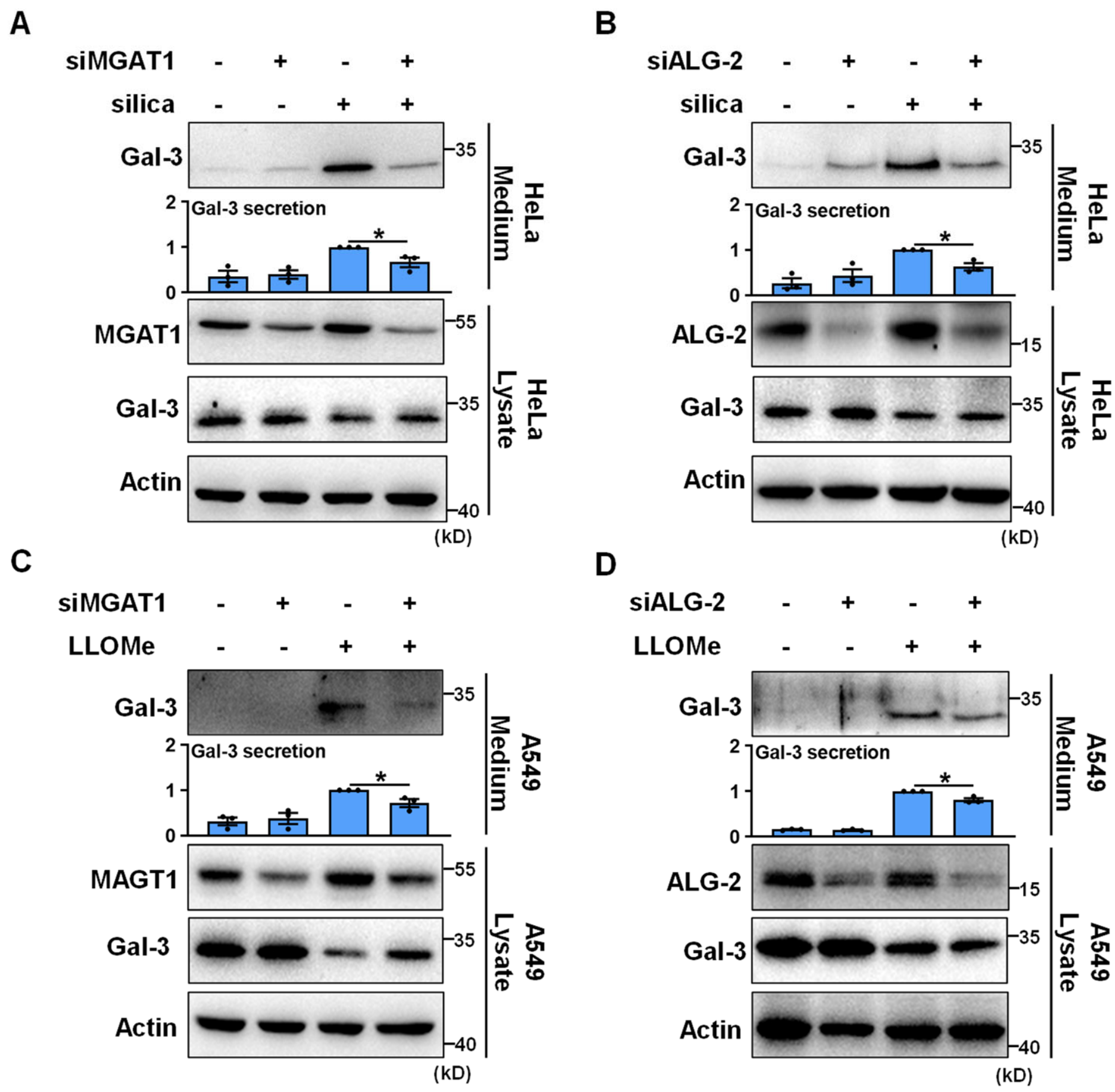

Supplementary Figure 9. MGAT1 and ALG-2 are involved in the secretion of Gal-3 in other lysosomal damage models.

(**A** and **B**) In the silica-induced lysosomal damage model, mannoside acetylglucosaminyltransferase 1 (MGAT1) (**A**) and ALG-2 (**B**) are all involved in Gal-3 secretion. MGAT1-knockdown (**A**) and ALG-2-knockdown (**B**) HeLa cells were treated with 2.5 μg/mL silica for 30 min, followed by 4 h of incubation in SFM after silica washout. Gal-3 in the medium and proteins in the cell lysates were examined by western blotting.

(**C** and **D**) MGAT1 (**C**) and ALG-2 (**D**) are also required for Gal-3 secretion upon lysosomal damage in A549 cells. MGAT1-knockdown (**C**) and ALG-2-knockdown (**D**) A549 cells were treated with 1 mM LLOMe for 30 min, followed by 4 h of incubation in SFM after LLOMe washout. Western blotting was used to analyze Gal-3 secretion into the medium and protein expression in cell lysates.

Bars indicate Gal-3 secretion. Data are shown as the mean ± SEM. *$p < 0.05$, two-tailed Student's *t*-test. $n = 3$ independent experiments.

Supplementary Figure 10

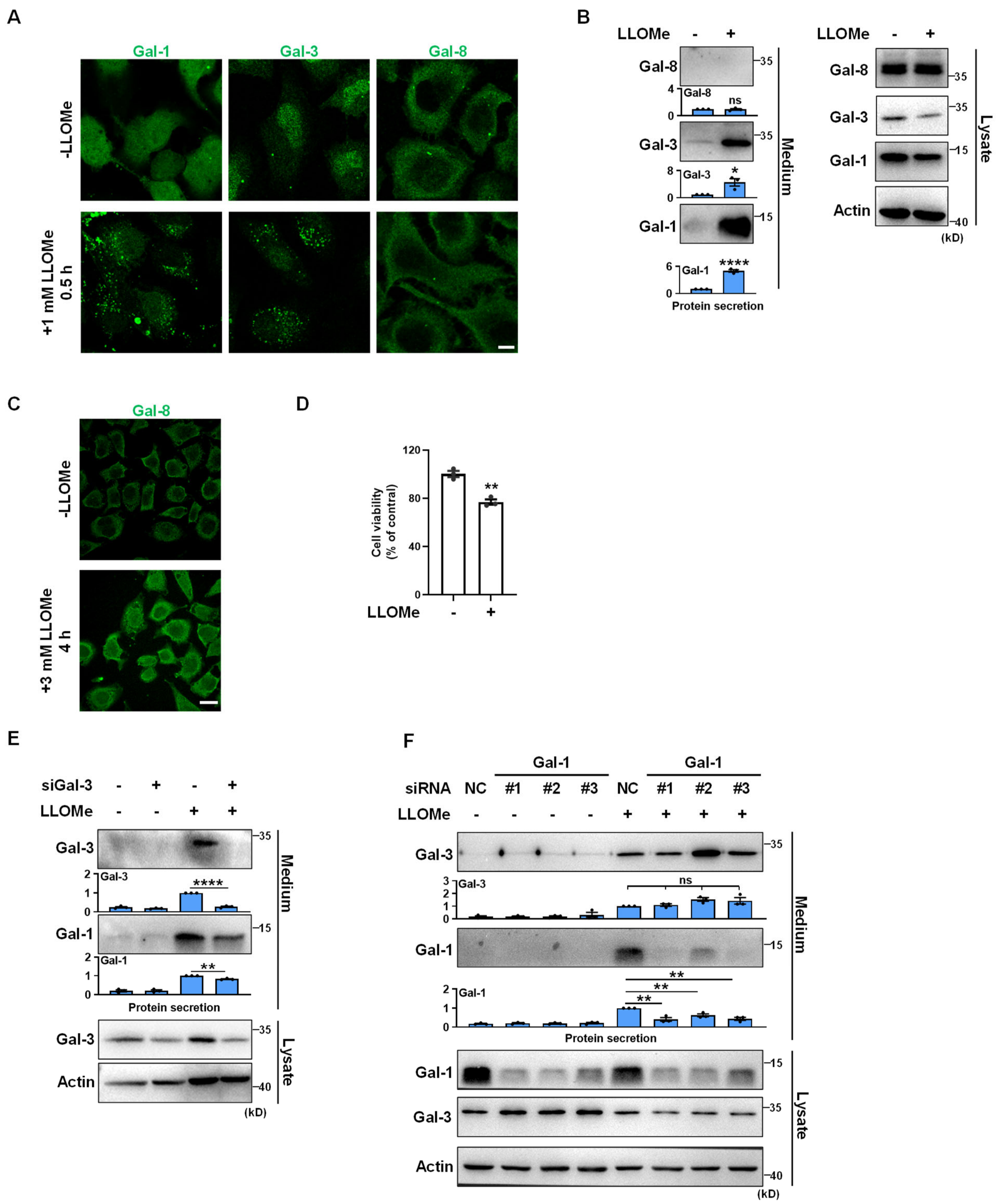

Supplementary Figure 10. Comparison of Galectin-1 (Gal-1), Gal-3, and Galectin-8 (Gal-8) secretion.

(**A**) Puncta formation of Gal-1, Gal-3, and Gal-8 in LLOMe-treated HeLa cells. Cells were treated with 1 mM LLOMe for 30 min and immunostained with antibodies.

(**B**) Secretion of Gal-1, Gal-3, and Gal-8 in LLOMe-treated HeLa cells. Cells were treated with 1 mM LLOMe for 30 min, followed by 4 h of incubation in SFM after LLOMe washout. Galectins in the medium and cell lysates were analyzed by western blotting. n = 3 independent experiments.

(**C**) Subcellular localization of Gal-8 in HeLa cells treated with a higher dose and longer duration of LLOMe. Cells were treated with 3 mM LLOMe for 4 h and immunostained with Gal-8 antibody.

(**D**) Cell viability under the treatment conditions in (**C**), assessed by the MTT (3-(4,5-dimethylthiazol-2-yl)-2,5-diphenyltetrazolium bromide) assay. n = 3 independent experiments.

(**E**) Gal-3 knockdown inhibits Gal-1 secretion. Gal-3-knockdown HeLa cells were treated with 1 mM LLOMe for 30 min, followed by 4 h of incubation in SFM after LLOMe washout. Gal-1 secretion and protein levels were analyzed by western blotting. n = 3 independent experiments.

(**F**) Gal-1 knockdown does not inhibit Gal-3 secretion. Gal-1-knockdown HeLa cells were treated with 1 mM LLOMe for 30 min, followed by 4 h of incubation in SFM after LLOMe washout. Gal-3 secretion and expression were examined by western blotting. n = 3 independent experiments.

Bars indicate galectin secretion. Data are shown as the means ± SEM. *$p < 0.05$, **$p < 0.01$, ****$p < 0.0001$, two-tailed Student’s *t*-test. Scale bars, 10 μm. ns, not significant.

Supplementary Figure 11

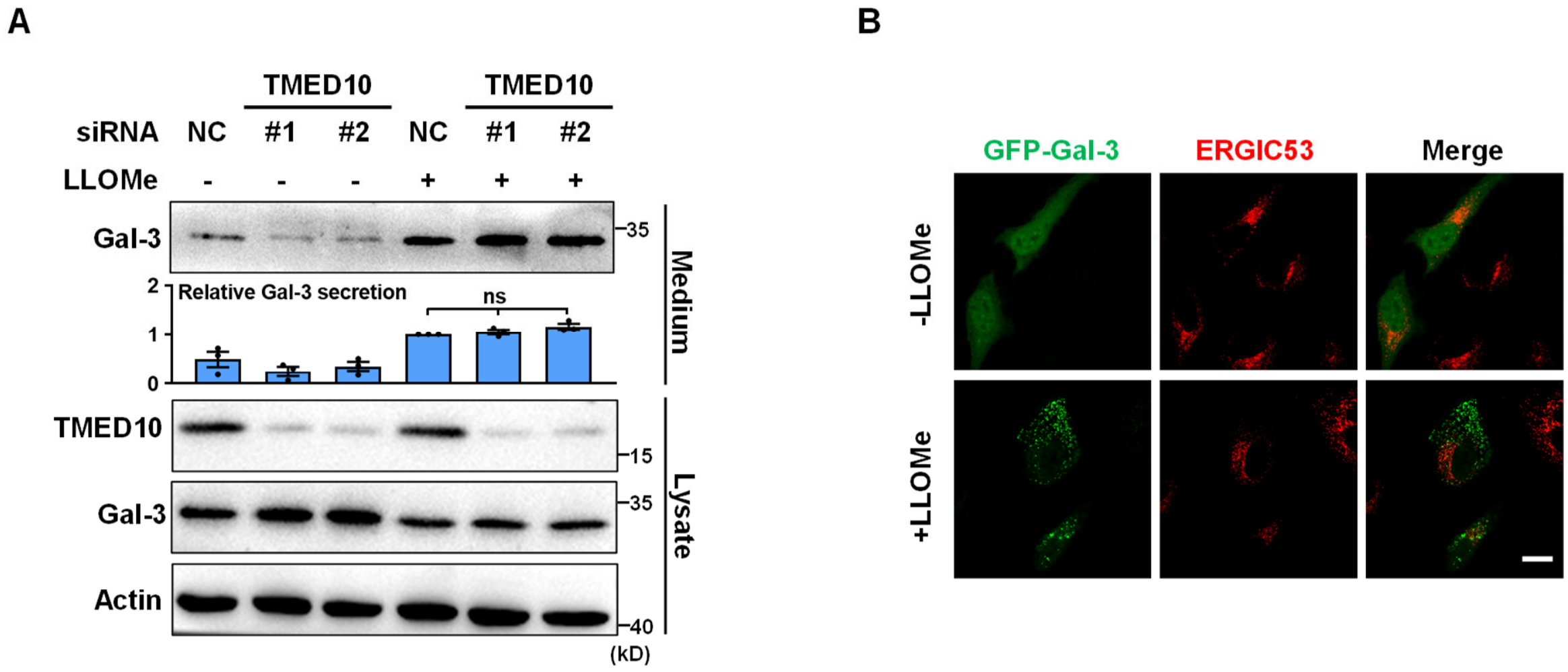

Supplementary Figure 11. Lysosomal damage-induced Gal-3 secretion is independent of TMED10 and ERGIC.

(**A**) Gal-3 secretion in TMED10 knockdown cells. HeLa cells were treated with 1 mM LLOMe for 30 min, followed by a 4 h incubation in SFM after LLOMe washout. The medium and cell lysates were analyzed by western blotting. n = 3 independent experiments.

(**B**) Representative images showing the absence of co-localization between Gal-3 and ERGIC53. HeLa cells transfected with the GFP-Gal-3 plasmid were treated with 1 mM LLOMe for 30 min and immunostained for ERGIC53.

Bars indicate Gal-3 secretion. Data are presented as mean ± SEM. Two-tailed Student's *t*-test. ns, not significant. Scale bars, 10 μm.

Supplementary Figure 12

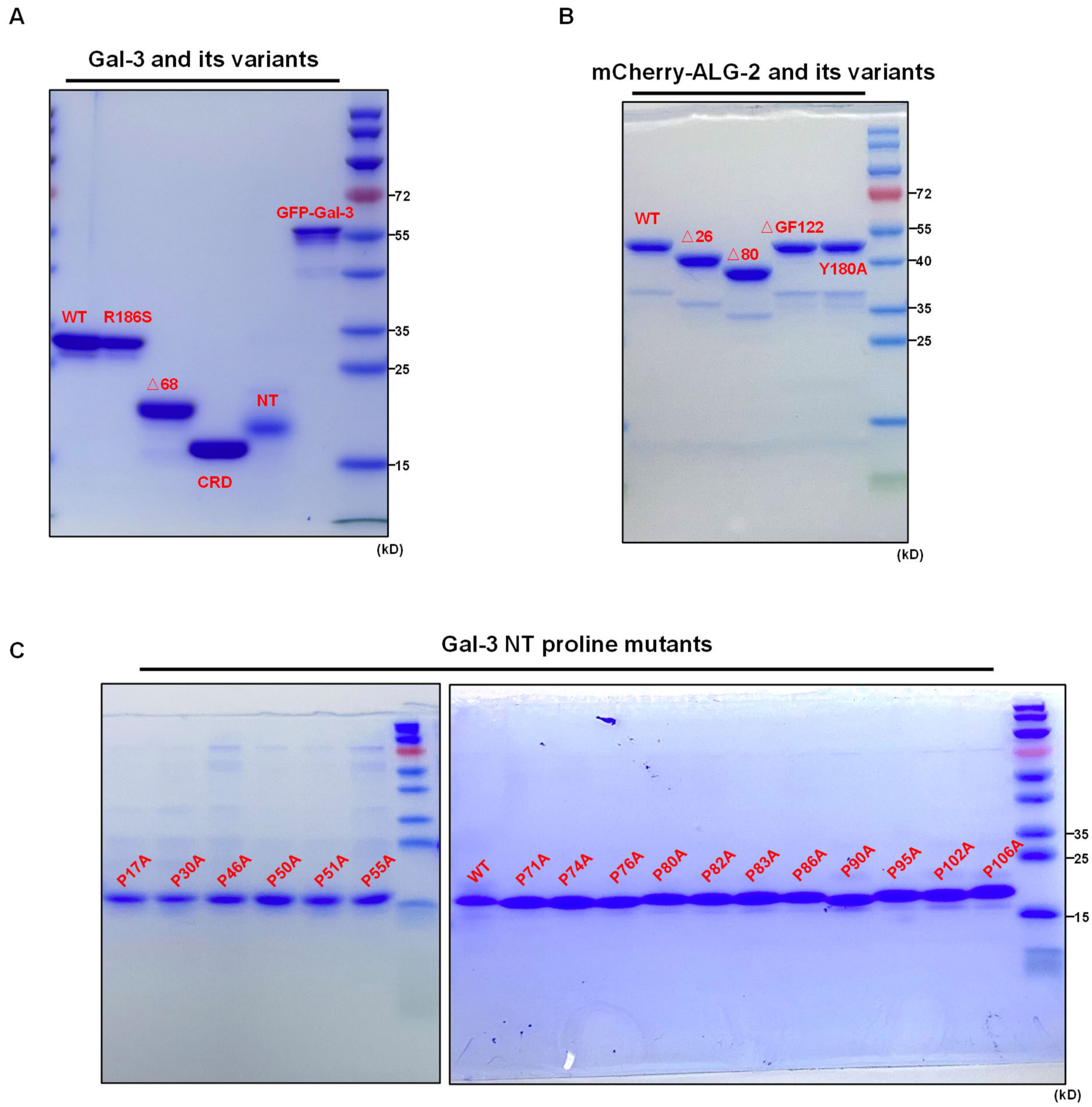

Supplementary Figure 12. SDS-PAGE analysis of purified recombinant proteins for Gal-3 variants (A), mCherry-ALG-2 mutants (B), and Gal-3 NT proline mutants (C).
