# Supplementary material for: Glycoprotein-induced phase separation drives unconventional secretion of galectin-3
Source: Nat Commun. 2026 Aug 6;17:9484. doi: 10.1038/s41467-026-76321-w (PMC13542199; doi:10.1038/s41467-026-76321-w)
Supplement: Supplementary file 2 [file 41467_2026_76321_MOESM2_ESM.pdf]

Description of Additional Supplementary Files

File Name: Supplementary Data 1

Description: Quantitative 100,000 g pellet-MS.

File Name: Supplementary Data 2

Description: Quantitative pulldown-MS.

File Name: Supplementary Data 3

Description: Plasmid information.

File Name: Supplementary Data 4

Description: Oligonucleotide sequences.
