# Supplementary material for: Glycoprotein-induced phase separation drives unconventional secretion of galectin-3
Source: Nat Commun. 2026 Aug 6;17:9484. doi: 10.1038/s41467-026-76321-w (PMC13542199; doi:10.1038/s41467-026-76321-w)
Supplement: Supplementary file 3 [file 41467_2026_76321_MOESM7_ESM.pdf]

nature portfolio

Corresponding author(s): Avraham Raz, Guihua Tai, Yifa Zhou

Last updated by author(s): Jun 25, 2026

Reporting Summary

Nature Portfolio wishes to improve the reproducibility of the work that we publish. This form provides structure for consistency and transparency in reporting. For further information on Nature Portfolio policies, see our Editorial Policies and the Editorial Policy Checklist.

Statistics

For all statistical analyses, confirm that the following items are present in the figure legend, table legend, main text, or Methods section.

n/a	Confirmed	
☐	☒	The exact sample size (n) for each experimental group/condition, given as a discrete number and unit of measurement
☐	☒	A statement on whether measurements were taken from distinct samples or whether the same sample was measured repeatedly
☐	☒	The statistical test(s) used AND whether they are one- or two-sided<br>*Only common tests should be described solely by name; describe more complex techniques in the Methods section.*
☐	☒	A description of all covariates tested
☒	☐	A description of any assumptions or corrections, such as tests of normality and adjustment for multiple comparisons
☐	☒	A full description of the statistical parameters including central tendency (e.g. means) or other basic estimates (e.g. regression coefficient) AND variation (e.g. standard deviation) or associated estimates of uncertainty (e.g. confidence intervals)
☐	☒	For null hypothesis testing, the test statistic (e.g. F, t, r) with confidence intervals, effect sizes, degrees of freedom and P value noted<br>*Give P values as exact values whenever suitable.*
☒	☐	For Bayesian analysis, information on the choice of priors and Markov chain Monte Carlo settings
☐	☒	For hierarchical and complex designs, identification of the appropriate level for tests and full reporting of outcomes
☒	☐	Estimates of effect sizes (e.g. Cohen's d, Pearson's r), indicating how they were calculated

Our web collection on statistics for biologists contains articles on many of the points above.

Software and code

Policy information about availability of computer code

Data collection	The data in this study were collected using the following instruments: Carl Zeiss LSM880 confocal laser scanning microscope, EASY-nLC 1200 liquid chromatographic system, Q-Exactive mass spectrometer, Brookhaven NanoBrook Omni instrument, PerkinElmer Operetta CLS high-content imaging system, ForteBio Octet RED96 instrument.
Data analysis	Data analysis was performed using the following software: GraphPad Prism 6 (ver. 8.4.3), Image J (ver. 1.54P), ForteBio Data Analysis Software (ver. 8.1.0.36), Carl Zeiss ZEN 2012 blue edition (ver. 1.1.2.0).

For manuscripts utilizing custom algorithms or software that are central to the research but not yet described in published literature, software must be made available to editors and reviewers. We strongly encourage code deposition in a community repository (e.g. GitHub). See the Nature Portfolio guidelines for submitting code & software for further information.

Data

Policy information about availability of data

All manuscripts must include a data availability statement. This statement should provide the following information, where applicable:

- Accession codes, unique identifiers, or web links for publicly available datasets
- A description of any restrictions on data availability
- For clinical datasets or third party data, please ensure that the statement adheres to our policy

The mass spectrometry proteomics data of the 100,000 × g pellet fraction and pulldown proteins are available in Figshare and ProteomeXchange as detailed in "Data availability."

Research involving human participants, their data, or biological material

Policy information about studies with human participants or human data. See also policy information about sex, gender (identity/presentation), and sexual orientation and race, ethnicity and racism.

Reporting on sex and gender	*Use the terms sex (biological attribute) and gender (shaped by social and cultural circumstances) carefully in order to avoid confusing both terms. Indicate if findings apply to only one sex or gender; describe whether sex and gender were considered in study design; whether sex and/or gender was determined based on self-reporting or assigned and methods used. Provide in the source data disaggregated sex and gender data, where this information has been collected, and if consent has been obtained for sharing of individual-level data; provide overall numbers in this Reporting Summary. Please state if this information has not been collected. Report sex- and gender-based analyses where performed, justify reasons for lack of sex- and gender-based analysis.*
Reporting on race, ethnicity, or other socially relevant groupings	*Please specify the socially constructed or socially relevant categorization variable(s) used in your manuscript and explain why they were used. Please note that such variables should not be used as proxies for other socially constructed/relevant variables (for example, race or ethnicity should not be used as a proxy for socioeconomic status). Provide clear definitions of the relevant terms used, how they were provided (by the participants/respondents, the researchers, or third parties), and the method(s) used to classify people into the different categories (e.g. self-report, census or administrative data, social media data, etc.) Please provide details about how you controlled for confounding variables in your analyses.*
Population characteristics	*Describe the covariate-relevant population characteristics of the human research participants (e.g. age, genotypic information, past and current diagnosis and treatment categories). If you filled out the behavioural & social sciences study design questions and have nothing to add here, write "See above."*
Recruitment	*Describe how participants were recruited. Outline any potential self-selection bias or other biases that may be present and how these are likely to impact results.*
Ethics oversight	*Identify the organization(s) that approved the study protocol.*

Note that full information on the approval of the study protocol must also be provided in the manuscript.

Field-specific reporting

Please select the one below that is the best fit for your research. If you are not sure, read the appropriate sections before making your selection.

☒ Life sciences ☐ Behavioural & social sciences ☐ Ecological, evolutionary & environmental sciences

For a reference copy of the document with all sections, see nature.com/documents/nr-reporting-summary-flat.pdf

Life sciences study design

All studies must disclose on these points even when the disclosure is negative.

Sample size	Each experiment was repeated independently at least two to four times.
Data exclusions	No data were excluded from the analyses.
Replication	All experiments performed were reproducible.
Randomization	Randomization was not applicable or performed during this study.
Blinding	This was not applicable to the study, all conditions were known.

Reporting for specific materials, systems and methods

We require information from authors about some types of materials, experimental systems and methods used in many studies. Here, indicate whether each material, system or method listed is relevant to your study. If you are not sure if a list item applies to your research, read the appropriate section before selecting a response.

Materials & experimental systems

n/a	Involved in the study
☐	☒ Antibodies
☐	☒ Eukaryotic cell lines
☒	☐ Palaeontology and archaeology
☒	☐ Animals and other organisms
☒	☐ Clinical data
☒	☐ Dual use research of concern
☒	☐ Plants

Methods

n/a	Involved in the study
☒	☐ ChIP-seq
☒	☐ Flow cytometry
☒	☐ MRI-based neuroimaging

Antibodies

Antibodies used

The primary antibodies used in this study were as follows:
mouse anti-Actin (clone C4) (Cat# 612657, BD Transduction Laboratories, 1:1000 for Western blotting),
mouse anti-ALG-2 (clone 2B4) (Cat# H00010016-M01, Abnova, 1:100 for immunofluorescence),
rabbit anti-ALG-2 (clone EP3259) (Cat# ab109181, Abcam, 1:1000 for Western blotting),
rabbit anti-ATG16L1 (clone D6D5) (Cat# 8089, Cell Signaling Technology, 1:1000 for Western blotting),
rabbit anti-Beclin1 (clone D40C5) (Cat# 3495, Cell Signaling Technology, 1:1000 for Western blotting),
rabbit anti-Caspase-3 (Cat# 9662, Cell Signaling Technology, 1:1000 for Western blotting),
rabbit anti-CD44 (clone E7K2Y) (Cat# 37259, Cell Signaling Technology, 1:1000 for Western blotting),
rabbit anti-CD46 (clone EPR4014) (Cat# ab108307, Abcam, 1:1000 for Western blotting),
rabbit anti-CD71 (clone EPR20584) (Cat# ab214039, Abcam, 1:1000 for Western blotting, 1:100 for immunofluorescence),
rabbit anti-CD146 (clone EPR3208) (Cat# ab75769, Abcam, 1:1000 for Western blotting),
rabbit anti-cleaved N-terminal GSDMD (clone EPR20829-408) (Cat# ab215203, Abcam, 1:1000 for Western blotting),
rabbit anti-ERGIC53 (Cat# 13364-1-AP, Proteintech, 1:100 for immunofluorescence),
rabbit anti-TMED10 (clone EPR9036(B)) (Cat# ab134948, Abcam, 1:1000 for Western blotting),
mouse anti-Flag (Cat# RM1002, Beijing Ray Antibody Biotech, 1:1000 for Western blotting),
rabbit anti-Galectin-1 (clone D608T) (Cat# 12936, Cell Signaling Technology, 1:1000 for Western blotting, 1:100 for immunofluorescence),
rabbit anti-Galectin-3 (clone D4I2R) (Cat# 87985, Cell Signaling Technology, 1:1000 for Western blotting, 1:100 for immunofluorescence),
mouse anti-Galectin-8 (clone VA-11.25) (Cat# G5671, Sigma-Aldrich, 1:1000 for Western blotting, 1:100 for immunofluorescence),
rabbit anti-GFP (clone D5.1) (Cat# 2956, Cell Signaling Technology, 1:1000 for Western blotting),
rabbit anti-GSDME (clone EPR19859) (Cat# ab215191, Abcam, 1:1000 for Western blotting),
mouse anti-His (Cat# RM1001, Beijing Ray Antibody Biotech, 1:1000 for Western blotting),
rabbit anti-LAMP1 (clone D2D11) (Cat# 9091, Cell Signaling Technology, 1:1000 for Western blotting, 1:100 for immunofluorescence),
rabbit anti-LAMP2 (Cat# MAB6228, R&D Systems, 1:100 for immunofluorescence),
rabbit anti-LAMP2 (clone EPR19512) (Cat# ab199946, Abcam, 1:1000 for Western blotting),
mouse anti-LC3B (clone E5Q2K) (Cat# 83506, Cell Signaling Technology, 1:1000 for Western blotting),
rabbit anti-MGAT1 (clone EPR14247) (Cat# ab180578, Abcam, 1:1000 for Western blotting),
rabbit anti-Rab7 (clone D95F2) (Cat# 9367, Cell Signaling Technology, 1:1000 for Western blotting, 1:100 for immunofluorescence),
rabbit anti-SNAP23 (Cat# ab3340, Abcam, 1:1000 for Western blotting),
rabbit anti-SNAP29 (clone EPR9199) (Cat# ab138500, Abcam, 1:1000 for Western blotting),
rabbit anti-Syntaxin 3 (Cat# ab101744, Abcam, 1:1000 for Western blotting),
rabbit anti-Syntaxin 4 (clone EPR15473) (Cat# ab184545, Abcam, 1:1000 for Western blotting),
rabbit anti-TRIM16 (Cat# ab72129, Abcam, 1:1000 for Western blotting),
mouse anti-ULK1 (clone D8H5) (Cat# 8054, Cell Signaling Technology, 1:1000 for Western blotting),
mouse anti-VAP-A (clone 4C12) (Cat# sc-293278, Santa Cruz Biotechnology, 1:100 for immunofluorescence).

The fluorescent secondary antibodies used in this study were as follows:
Cy3 Goat Anti-Mouse IgG (H+L) (Cat# AS008, Abclonal, 1:100 for immunofluorescence),
Cy3 Goat Anti-Rabbit IgG (H+L) (Cat# AS007, Abclonal, 1:100 for immunofluorescence),
FITC Goat Anti-Mouse IgG (H+L) (Cat# AS001, Abclonal, 1:100 for immunofluorescence),
FITC Goat Anti-Rabbit IgG (H+L) (Cat# AS011, Abclonal, 1:100 for immunofluorescence).

Validation

Antibodies used in this study are commercially available. The validations could be found in manufacturer's website.
The primary antibodies used in this study were as follows:
mouse anti-Actin; https://www.bdbiosciences.com/zh-cn/products/reagents/western-blotting-and-molecular-reagents/western-blot-reagents/purified-mouse-anti-actin-ab-5.612657?tab=product_details,
mouse anti-ALG-2; https://www.abnova.com/zh-cn/product/detail/h00010016-m01,
rabbit anti-ALG-2; https://www.abcam.cn/products/primary-antibodies/pdcd6-alg-2-antibody-ep3259-ab109181

rabbit anti-ATG16L1; https://www.cellsignal.cn/products/primary-antibodies/atg16l1-d6d5-rabbit-monoclonal-antibody/8089
rabbit anti-Beclin1; https://www.cellsignal.cn/products/primary-antibodies/beclin-1-d40c5-rabbit-monoclonal-antibody/3495
rabbit anti-Caspase-3; https://www.cellsignal.cn/products/primary-antibodies/caspase-3-antibody/9662
rabbit anti-CD44; https://www.cellsignal.cn/products/primary-antibodies/cd44-e7k2y-rabbit-monoclonal-antibody/37259
rabbit anti-CD46; https://www.abcam.cn/products/primary-antibodies/cd46-antibody-epr4014-ab108307
rabbit anti-CD71; https://www.abcam.cn/products/primary-antibodies/transferrin-receptor-antibody-epr20584-ab214039
rabbit anti-CD146; https://www.abcam.cn/products/primary-antibodies/cd146-antibody-epr3208-ab75769
rabbit anti-cleaved N-terminal GSDMD; https://www.abcam.cn/products/primary-antibodies/cleaved-n-terminal-gsdmd-antibody-epr20829-408-ab215203
rabbit anti-ERGIC53; https://www.ptgcn.com/products/LMAN1-Antibody-13364-1-AP.htm
rabbit anti-TMED10; https://www.abcam.cn/products/primary-antibodies/tmp21-antibody-epr9036b-ab134948
mouse anti-Flag; http://www.rayantibody.com/index.php?a=search&c=product&word=RM1002
rabbit anti-Galectin-1; https://www.cellsignal.cn/products/primary-antibodies/galectin-1-lgals1-d608t-rabbit-monoclonal-antibody/12936
rabbit anti-Galectin-3; https://www.cellsignal.cn/products/primary-antibodies/galectin-3-lgals3-d4i2r-rabbit-monoclonal-antibody/87985
mouse anti-Galectin-8; https://www.sigmaaldrich.cn/CN/zh/product/sigma/g5671
rabbit anti-GFP; https://www.cellsignal.cn/products/primary-antibodies/gfp-d5-1-rabbit-monoclonal-antibody/2956
rabbit anti-GSDME; https://www.abcam.cn/products/primary-antibodies/dfna5-gsdme-antibody-epr19859-n-terminal-ab215191
mouse anti-His; http://www.rayantibody.com/uploads/2014/07/101634433600.pdf
rabbit anti-LAMP1; https://www.cellsignal.cn/products/primary-antibodies/lamp1-d2d11-rabbit-monoclonal-antibody/9091
rabbit anti-LAMP2; https://www.rndsystems.com/products/human-lamp2-cd107b-antibody-743320_mab6228
rabbit anti-LAMP2; https://www.abcam.cn/products/primary-antibodies/lamp2-antibody-epr19512-lysosome-marker-ab199946
mouse anti-LC3B; https://www.cellsignal.cn/products/primary-antibodies/lc3b-e5q2k-mouse-monoclonal-antibody/83506
rabbit anti-MGAT1; https://www.abcam.cn/products/primary-antibodies/mgat1-antibody-epr14247-ab180578
rabbit anti-Rab7; https://www.cellsignal.cn/products/primary-antibodies/rab7-d95f2-rabbit-monoclonal-antibody/9367
rabbit anti-SNAP23; https://www.abcam.cn/products/primary-antibodies/snap23-antibody-ab3340
rabbit anti-SNAP29; https://www.abcam.cn/products/primary-antibodies/snap29-antibody-epr9199-ab138500
rabbit anti-Syntaxin 3; https://doc.abcam.com/legacy-unpublished/datasheets/com/datasheet_101744.pdf
rabbit anti-Syntaxin 4; https://www.abcam.cn/products/primary-antibodies/syntaxin-4-antibody-epr15473-ab184545
rabbit anti-TRIM16; https://www.abcam.cn/products/primary-antibodies/trim16-antibody-ab72129
mouse anti-ULK1; https://www.cellsignal.cn/products/primary-antibodies/ulk1-d8h5-rabbit-monoclonal-antibody/8054
mouse anti-VAP-A; https://www.scbt.com/p/vap-a-antibody-4c12

The fluorescent secondary antibodies used in this study were as follows:
Cy3 Goat Anti-Mouse IgG (H+L); https://abclonal.com.cn/catalog/AS008
Cy3 Goat Anti-Rabbit IgG (H+L); https://abclonal.com.cn/catalog/AS007
FITC Goat Anti-Mouse IgG (H+L); https://abclonal.com.cn/catalog/AS001
FITC Goat Anti-Rabbit IgG (H+L); https://abclonal.com.cn/catalog/AS011

Eukaryotic cell lines

Policy information about cell lines and Sex and Gender in Research

Cell line source(s): HeLa (Cat# CCL-2) and RAW264.7 (Cat# TIB-71) cell lines were obtained from American Type Culture Collection (ATCC). A549 (Cat# SCSP-503), HT-22 (Cat# GNM47), U2OS (Cat# SCSP-5030), and Hepa 1-6 (Cat# SCSP-512) cell lines were obtained from the Cell Bank/Stem Cell Bank of the Chinese Academy of Sciences.

Authentication: The cell lines were not authenticated because it was from ATCC and Cell Bank/Stem Cell Bank of the Chinese Academy of Sciences.

Mycoplasma contamination: Cell lines tested negative for mycoplasma.

Commonly misidentified lines (See ICLAC register): No commonly misidentified lines were used.

Plants

Seed stocks: *Report on the source of all seed stocks or other plant material used. If applicable, state the seed stock centre and catalogue number. If plant specimens were collected from the field, describe the collection location, date and sampling procedures.*

Novel plant genotypes: *Describe the methods by which all novel plant genotypes were produced. This includes those generated by transgenic approaches, gene editing, chemical/radiation-based mutagenesis and hybridization. For transgenic lines, describe the transformation method, the number of independent lines analyzed and the generation upon which experiments were performed. For gene-edited lines, describe the editor used, the endogenous sequence targeted for editing, the targeting guide RNA sequence (if applicable) and how the editor was applied.*

Authentication: *Describe any authentication procedures for each seed stock used or novel genotype generated. Describe any experiments used to assess the effect of a mutation and, where applicable, how potential secondary effects (e.g. second site T-DNA insertions, mosiacism, off-target gene editing) were examined.*
