# Supplementary material for: Glycoprotein-induced phase separation drives unconventional secretion of galectin-3
Source: Nat Commun. 2026 Aug 6;17:9484. doi: 10.1038/s41467-026-76321-w (PMC13542199; doi:10.1038/s41467-026-76321-w)
Supplement: Supplementary file 4 [file 41467_2026_76321_MOESM8_ESM.pdf]

nature portfolio

Peer Review File

Glycoprotein-induced phase separation drives unconventional secretion of galectin-3

Corresponding Author: Professor Guihua Tai

Parts of this Peer Review File have been redacted as indicated to remove third-party material.

This file contains all reviewer reports in order by version, followed by all author rebuttals in order by version.

Version 1:

Reviewer comments:

Reviewer #1

(Remarks to the Author)
The manuscript by Tai and colleagues investigates an unconventional protein secretion (UcPS) pathway for Galectin-3 (Gal-3), a cytosolic glycan-binding lectin. Gal-3 has previously been described as a UcPS cargo, mainly through ESCRT-dependent intraluminal vesicle formation. Here, the authors report that Gal-3 can also be secreted under lysosomal damage, via a mechanism that appears distinct from earlier reports. They propose that Gal-3 interacts broadly with glycoproteins, undergoes liquid–liquid phase separation (LLPS), and recruits additional factors such as ALG-2 and DNAJC5 in a glycoprotein-dependent manner. This LLPS-based assembly is presented as a novel secretory mechanism that may enable cells to adapt to lysosomal damage.
While the direction is intriguing and the potential role of LLPS in UcPS is of interest, the study in its current form has major limitations. The evidence presented does not adequately support the proposed model, and the manuscript feels preliminary for publication in Nature Communications.

Major concerns
1. Lack of lysate controls
Most secretion assays do not include cell lysate blots to demonstrate Gal-3 levels in cells. Without this control, changes in secretion cannot be distinguished from changes in protein expression or stability. Similarly, cytosolic leakage controls are missing, making it difficult to exclude secretion artifacts from membrane damage.
2. Clarity and logic
The manuscript is overly concise and often lacks sufficient rationale for experimental design. The presentation of results is sometimes contradictory or illogical. Moreover, it is frequently ambiguous whether endogenous or overexpressed/tagged proteins are being analyzed, obscuring the significance of their findings
3. Experimental details
Many key conditions are insufficiently described, limiting reproducibility. For example, the choice of glycoproteins in Figure 2 is not well justified, and critical quantification is often missing.

Specific points
Figure 1
• Cell lysate and cytosolic leakage controls are essential.
• Quantification in panels H, I, and K does not match the gels.
• The distinction between endogenous and overexpressed proteins must be clarified throughout the text.
• Line 106: pyroptosis is dismissed without explanation.
Figure 2
• The rationale for focusing on glycoproteins is unclear, as pulldown identified both glycosylated and non-glycosylated proteins.
• Why prioritize CD71, LAMP1, and LAMP2? Were they the most abundant? Why does knockdown of each individually reduce Gal-3 secretion if they are equally abundance in cells?
• Some intriguing results (e.g., Gal-3 secretion after ™ treatment, or upon O-glycosylation inhibition) are shown but not discussed.
• Quantification of secretion assays is needed.

Figure 3
• Gal-3 puncta at damaged lysosomes cannot be taken as evidence of LLPS in cells.
• Despite evidence that glycoproteins promote Gal-3 LLPS in vitro, the role of glycans per se was not tested (e.g., with synthetic glycans or Endo H treatment).
• It is unclear why the Gal-3 CRD domain, insufficient for LLPS in vitro, still forms puncta in LLOMe-treated cells.
• Secretion assays again lack lysate controls.
Figure 4
• ALG-2 and DNAJC5 interactions are weakly supported by few peptides in pulldown; endogenous validation is required.
• The text alternates between describing Gal-3 puncta as aggregates and LLPS, which is inconsistent.
• Images in panel E are unconvincing; panel H lacks appropriate controls. Pharmacological inhibitors of LLPS are not specific enough—genetic or biochemical evidence is needed.
Figure 5
• The proposed link between LLPS and ALG-2 recruitment is undermined by in vitro data showing ALG-2 binding without LLPS. This contradiction needs resolution.
• The reported affinity of ALG-2 for Gal-3 (Kd ~250 μM) is very weak and conflicts with the N-domain binding data. Could synthetic glycans improve affinity?
• AlphaFold modeling could strengthen the analysis, followed by biochemical validation.
• Figure 5H is preliminary; proper Kd measurements for mutants are needed.
• All purified proteins should be shown on a gel.
Figure 6
• The claim that Gal-3 translocates to the ER contradicts earlier data (Figure 2) showing localization to damaged lysosomes. The discrepancy is not explained.
Figure 7
• The inclusion of DNAJC5 in Gal-3 LLPS is interesting but currently underdeveloped. This part suffers the same caveats as listed above. This section could be removed to focus on strengthening the main findings.

Summary
The proposed mechanism linking Gal-3 secretion, glycoprotein binding, and LLPS is conceptually exciting, but the current dataset has major weaknesses. The manuscript requires substantial experimental reinforcement, particularly with rigorous controls, clear logic, and stronger validation of key interactions, before it can be considered for publication in Nature Communications.

Reviewer #2

(Remarks to the Author)
In this manuscript Zhao et al put together a compelling story that Gal-3 is secreted by the UPS pathway during phase separation. Using a combination of cell biology, microscopy, and biophysics they demonstrate that Gal-3 interacts with N-glycan on lysosomal proteins of to mediate this secretion. This was further demonstrated to be through interactions with the NT domain of Gal-3 as Gal-3 NT KO variants did not demonstrate secretion or phase separation. Overall I think this manuscript represents a strong contribution to the field and would of interest to a broad readership.

Some suggestions on how this manuscript could be improved include

1.) chemical and genetic manipulation of glycosylation was used to implicate hybrid and complex N-glycans, however this analysis does not specify which glycoforms are driving these interactions and if the glycoproteins implicated (e.g. lamp1) is known to have these glycans.

2.) in vitro experiments could be bolstered by PNGaseF treatment of proteins to demonstrate that LLPS formation is N-glycan dependent in vitro.

3.) figure 5 BLI data is difficult to follow what is present on each surface, maybe use a pictoral representation to describe the complexes on each BLI tip surface

4.) contextualization of Gal-3 pahse separation in the stickers and linkers model will help undertand the molecular basis of these interactions.

5.) Figure 4A, please indicate which bands are which protein (or indicate each proteins molecular weight) on the silver stained pulled down gel.

Reviewer #3

(Remarks to the Author)
In this manuscript, the authors describe that Galectin-3 (Gal-3) undergoes an unconventional protein secretion pathway driven by glycoprotein-induced phase separation, leading to the formation of large non-exosomal aggregates independent of pyroptosis or secretory autophagy. These aggregates recruit ALG-2 and DNAJC5, which guide them from the ER–late endosome interface into late endosomes. Finally, Gal-3 is exported into the extracellular space via SNARE-dependent vesicular transport, acting as a damage-associated “clean-up” mechanism.

I find the idea of glycoprotein-induced phase separation convincing, as it makes conceptual sense. However, my main concern lies in the proposed topology. LAMP1, LAMP2, and CD71 are transmembrane proteins, and it is difficult to understand how such membrane-anchored proteins could be incorporated into condensates without simultaneously bringing along their associated lipid bilayer. Similarly, it is unclear how a protein condensate would enter late endosomes without some form of invagination process, which would necessarily enclose the condensate within a membrane. These are fundamental principles of membrane biology that generally cannot be violated.

A more plausible alternative model is that Gal-3 enters damaged lysosomes, where it forms condensates with glycoproteins on the luminal side of the lysosomal membrane. These damaged lysosomes could then directly, or indirectly via late endosomes, release the condensates into the extracellular space through fusion with the plasma membrane. I recommend that the authors consider such an alternative model, which may be more consistent with established principles of membrane biology.

Version 2:

Reviewer comments:

Reviewer #2

(Remarks to the Author)
I think the edits made have strengthened the manuscript and it is now suitable for publication in Nature communications.

Reviewer #3

(Remarks to the Author)
The authors have addressed my queries satisfactorily. Although I am not completely convinced by the response to point 2, this is a minor detail and should not hinder publication. I would suggest that the authors discuss this point in the manuscript if they wish to do so.

Reviewer #4

(Remarks to the Author)
This is an intriguing study demonstrating that the phase separation of Gal3, mediated by its binding to glycans exposed upon lysosomal damage, facilitates the UcPS (unconventional protein secretion) of Gal3. Overall, the authors have presented a substantial amount of data in the revised version. However, I have a few additional suggestions that could enhance the work:
1. TMED10 Involvement: Multiple studies have shown that TMED10 regulates the release of galectins, including Gal1, Gal3, and Gal9 (PMID: 38926505; PMID: 32272059; PMID: 40335523; PMID: 41364076). It would be beneficial to investigate the role of TMED10 in lysosomal damage-related systems and its potential involvement in Gal3 secretion.
2. ESCRT III Components: Given that ESCRTs are involved in lysosome repair, it would be valuable to test the involvement of ESCRT III components in Gal3 secretion to further validate the proposed model.
3. ERGIC Localization: The ERGIC localization of Gal3 should also be tested in the context of lysosomal damage, alongside the ER and lysosomal markers already presented in the paper.
4. ER-Lysosome Contact: Is the ER-lysosome contact essential for Gal3 secretion, or is it merely coincidental? RNAi of VAPA could help determine whether this contact is required for Gal3 secretion.
5. Molecular Weight Markers in WB: All molecular weight markers should be clearly labeled in Western blot gels for better clarity.
6. Figure 2 - Glycoprotein Depletion: In Figure 2, numerous glycoproteins are capable of binding Gal3. Why does depleting just one, such as LAMP1, affect Gal3 secretion? There should be other glycoproteins that can still bind Gal3 and facilitate its release.
7. Figure 1C - Gal3 Decrease: In Figure 1C, there is a noticeable decrease in Gal3 in the medium after 4 hours. Could you provide more insight into the reason for this reduction?
8. Line 40 - Terminology Update: The term "exosome-mediated release" should be updated to "extracellular vesicle-mediated release" since exosomes represent only a subclass of these vesicles.
9. Line 41 - Terminology Update: In line 41, "TMED10-gated" should be changed to "TMED10-mediated."
10. Line 41 - MAPS System: The MAPS system of UcPS (PMID: 27295555) should also be mentioned for completeness.
11. Abbreviation for UcPS: Please update the abbreviation "UPS" to "UcPS" as it refers to unconventional protein secretion.

Version 3:

Reviewer comments:

Reviewer #4

(Remarks to the Author)
The manuscript has been significantly improved, and the authors have addressed the majority of my previous concerns. However, one issue remains that should be addressed before the manuscript can be accepted for publication.

In the TMED10 RNAi experiments, galectin-3 secretion is not reduced under basal conditions prior to LLOME treatment. This raises concerns that the knockdown efficiency may be insufficient to effectively impair the unconventional protein secretion (UcPS) function of TMED10, as multiple previous studies have demonstrated that TMED10 is required for galectin secretion under steady-state conditions. Therefore, the authors should validate their conclusions using TMED10 knockout cells together with TMED10 re-expression rescue experiments.

Alternatively, if the authors prefer to continue using RNAi, they should improve the knockdown efficiency and demonstrate the expected positive control phenotype — namely, that TMED10 depletion reduces galectin-3 secretion under steady-state conditions — before drawing conclusions regarding the effects of LLOME treatment.

Version 4:

Reviewer comments:

Reviewer #4

(Remarks to the Author)
I endorse this excellent work for publication.

Open Access This Peer Review File is licensed under a Creative Commons Attribution 4.0 International License, which permits use, sharing, adaptation, distribution and reproduction in any medium or format, as long as you give appropriate credit to the original author(s) and the source, provide a link to the Creative Commons license, and indicate if changes were made.
In cases where reviewers are anonymous, credit should be given to 'Anonymous Referee' and the source.
The images or other third party material in this Peer Review File are included in the article’s Creative Commons license, unless indicated otherwise in a credit line to the material. If material is not included in the article’s Creative Commons license and your intended use is not permitted by statutory regulation or exceeds the permitted use, you will need to obtain permission directly from the copyright holder.
To view a copy of this license, visit https://creativecommons.org/licenses/by/4.0/

REVIEWER COMMENTS

Reviewer #1 (Remarks to the Author):

The manuscript by Tai and colleagues investigates an unconventional protein secretion (UcPS) pathway for Galectin-3 (Gal-3), a cytosolic glycan-binding lectin. Gal-3 has previously been described as a UcPS cargo, mainly through ESCRT-dependent intraluminal vesicle formation. Here, the authors report that Gal-3 can also be secreted under lysosomal damage, via a mechanism that appears distinct from earlier reports. They propose that Gal-3 interacts broadly with glycoproteins, undergoes liquid–liquid phase separation (LLPS), and recruits additional factors such as ALG-2 and DNAJC5 in a glycoprotein-dependent manner. This LLPS-based assembly is presented as a novel secretory mechanism that may enable cells to adapt to lysosomal damage.

While the direction is intriguing and the potential role of LLPS in UcPS is of interest, the study in its current form has major limitations. The evidence presented does not adequately support the proposed model, and the manuscript feels preliminary for publication in Nature Communications.

Major concerns

1. Lack of lysate controls

Most secretion assays do not include cell lysate blots to demonstrate Gal-3 levels in cells. Without this control, changes in secretion cannot be distinguished from changes in protein expression or stability. Similarly, cytosolic leakage controls are missing, making it difficult to exclude secretion artifacts from membrane damage.

Response: Thank you for your comments. To address these points directly, we have performed the following experiments.

First, to confirm that Gal-3 secretion is independent of its expression level, we assessed Gal-3 levels in whole-cell lysates in all of our secretion assays. These results (added in Figures 1, S2, S4, S5, 2, 3, 4, 8, S9, and S10 of the revised manuscript) demonstrate that the total cellular levels of Gal-3 do not increase, whereas extracellular Gal-3 is increased. Conversely, the cellular level of Gal-3 is markedly decreased in the case that extracellular Gal-3 is markedly increased. These results confirm that the accumulation of Gal-3 in the medium is a result of enhanced secretion.

Second, to exclude the possibility that Gal-3 secretion in the medium is an artifact of cytosolic leakage caused by non-specific membrane damage or cell death, we measured the release of LDH, a cytosolic leakage marker, in parallel with our Gal-3 secretion models. Our data show that, in our lysosomal damage-induced Gal-3 secretion models, the levels of LDH in the supernatant were not elevated. This result indicates that the integrity of the plasma membrane was maintained and that the release of Gal-3 is a specific, regulated process, and not passive leakage. We have added this result to Figures 1 and S2.

2. Clarity and logic

The manuscript is overly concise and often lacks sufficient rationale for experimental design. The presentation of results is sometimes contradictory or illogical. Moreover, it is frequently ambiguous whether endogenous or overexpressed/tagged proteins are being analyzed, obscuring the significance of their findings

Response: We have thoroughly revised the manuscript to provide a stronger justification for the experimental design and to ensure a more logical flow. The Results section has been carefully restructured to eliminate any perceived contradictions or illogical progressions. Furthermore, we have now explicitly stated in figure legends whether endogenous or overexpressed proteins were analyzed in each experiment to eliminate all ambiguity.

3. Experimental details

Many key conditions are insufficiently described, limiting reproducibility. For example, the choice of glycoproteins in Figure 2 is not well justified, and critical quantification is often missing.

Response: We have added more experimental detail. Regarding the choice of glycoproteins, please see our response to Specific Points in Figure 2. Furthermore, we have added quantitative data of secretion assays in Figures 1, S2, S4, S5, 2, 3, 4, 8, S9, and S10.

Specific points

Figure 1

• Cell lysate and cytosolic leakage controls are essential.

Response: In response to your comment, we have detected cellular Gal-3 levels and measured LDH release into the medium. These results are added in revised manuscript as shown in Figures 1, S2, S4, S5, 2, 3, 4, 8, S9, and S10.

• Quantification in panels H, I, and K does not match the gels.

Response: The quantifications in panels H, I, and K are derived from ELISA assays that measure secreted Gal-3 in the cell culture media. The gels shown in panels D–G are representative western blotting images, for which we have now provided the corresponding quantitative analysis in the figure.

• The distinction between endogenous and overexpressed proteins must be clarified throughout the text.

Response: We have clarified the distinction between endogenous and overexpressed proteins in the legends for Figures 1, S1, S3, 3, 4, 7, and S10.

• Line 106: pyroptosis is dismissed without explanation.

Response: We have added the explanation (that was in Supplementary Information in original version) in the revised manuscript (Lines 101-114).

Figure 2

• The rationale for focusing on glycoproteins is unclear, as pulldown identified both glycosylated and non-glycosylated proteins.

Response: The rationale for focusing on glycoproteins is as follows. To screen potential proteins related to Gal-3 secretion, we performed MS analysis and western blotting with the culture supernatant to identify proteins co-released with Gal-3. This has shown that, upon lysosomal damage, ~21% of the total protein co-released with Gal-3 is comprised of glycoproteins. This finding is intriguing because glycoproteins are physiological ligands of Gal-3. Moreover, some of the most upregulated glycoproteins (e.g., LAMP1, CD71 and LAMP2) have been shown to mediate recruitment of Gal-3 to damaged lysosomes and function in lysosomal repair[1, 2]. In normal cells, these lysosomal glycoproteins reside on the luminal side of lysosomes, but are exposed to the cytoplasm

(a reservoir of Gal-3) when lysosome is damaged. Their co-release suggests a possible role for lysosomal glycoproteins in Gal-3 secretion. Based on these findings, we decided to focus our investigation on glycosylated proteins. To clarify the rational, we have added discussions of this in the revised manuscript (Lines 150-156).

Regarding the pulldown assay, after demonstrating that glycoprotein-triggered Gal-3 condensation mediated Gal-3 secretion, we performed the pulldown assay with cell lysate to identify potentially functional cytoplasmic proteins involved in Gal-3 secretion. This assay revealed a novel ligand, namely ALG-2, in addition to some classical Gal-3 ligands such as glycoproteins.

1. Jia J, et al. Galectin-3 coordinates a cellular system for lysosomal repair and removal. Dev Cell **52**, 69-87.e68 (2020).

2. Aits S, et al. Sensitive detection of lysosomal membrane permeabilization by lysosomal galectin puncta assay. Autophagy **11**, 1408-1424 (2015).

• Why prioritize CD71, LAMP1, and LAMP2? Were they the most abundant? Why does knockdown of each individually reduce Gal-3 secretion if they are equally abundance in cells?

Response: CD71, LAMP1, and LAMP2 are indeed the most abundant lysosomal glycoproteins. Furthermore, they are (i) the most upregulated glycoproteins co-released with Gal-3, (ii) well-characterized in terms of glycosylation and have been reported to interact with Gal-3[1,2,3], and (iii) known to mediate recruitment of Gal-3 to damaged lysosomes and function in lysosomal repair[1,2,3]. These characteristics make them prime candidates for mediating Gal-3 recruitment and secretion following lysosomal damage, although we cannot eliminate the possibility that other lysosomal glycoproteins are also involved.

Regarding the result of knocking down each glycoprotein, our explanation is as follows. As demonstrated previously, as well as in this study, Gal-3 recognizes *N*-glycans that are present in multiple lysosomal glycoproteins. These glycoproteins act as a platform for Gal-3 binding. Depleting any one of the abundant glycoproteins leads to a local reduction in overall glycan density on damaged membranes. This compromised glycan platform is less efficient at recruiting Gal-3, thereby impairing phase separation that is essential for secretion. This assumption is consistent with our previous study that Gal-3 LLPS is dependent on both glycoprotein and Gal-3 concentration[4]. When the

concentration of Gal-3 in the surroundings is fixed, the ability of Gal-3 LLPS is proportional to the concentration of glycoproteins[4].

1. Zhang J, Zeng W, Han Y, Lee WR, Liou J, Jiang Y. Lysosomal LAMP proteins regulate lysosomal pH by direct inhibition of the TMEM175 channel. Mol Cell **83**, 2524-2539.e2527 (2023).

2. Jia J, et al. Galectin-3 Coordinates a Cellular System for Lysosomal Repair and Removal. Dev Cell **52**, 69-87.e68 (2020).

3. Aits S, et al. Sensitive detection of lysosomal membrane permeabilization by lysosomal galectin puncta assay. Autophagy **11**, 1408-1424 (2015).

4. Zhao Z, et al. Galectin-3 N-terminal tail prolines modulate cell activity and glycan-mediated oligomerization/phase separation. Proceedings of the National Academy of Sciences of the United States of America **118**, (2021).

• Some intriguing results (e.g., Gal-3 secretion after ™ treatment, or upon O-glycosylation inhibition) are shown but not discussed.

Response: We have added discussion on this point in our revised manuscript (Lines 567-578).

• Quantification of secretion assays is needed.

Response: We have added quantification results of the secretion assays in Figures 1, S2, S4, S5, 2, 3, 4, 8, S9, and S10.

Figure 3

• Gal-3 puncta at damaged lysosomes cannot be taken as evidence of LLPS in cells.

Response: We agree that the presence of puncta alone is not definitive proof of LLPS. In our study, we provided direct experimental evidence for the liquid-like properties of Gal-3 condensates by FRAP analysis (Figures 3M and 3N). These data show the rapid fluorescence recovery that is a hallmark of dynamic condensates. This result is further strengthened by our previous work showing that, when encountered some glycoproteins, Gal-3 undergoes LLPS in solution and condensation on plasma membranes and endomembranes[1].

1. Zhao Z, et al. Galectin-3 N-terminal tail prolines modulate cell activity and glycan-mediated oligomerization/phase separation. Proceedings of the National Academy of Sciences of the United States of America **118**, (2021).

• Despite evidence that glycoproteins promote Gal-3 LLPS in vitro, the role of glycans per se was not tested (e.g., with synthetic glycans or Endo H treatment).

Response: We have performed a PNGase F digestion to remove N-glycans on LAMP1 and then assessed/compared its ability to induce phase separation with the intact LAMP1. The figures below show that PNGase F-treated LAMP1 induces minimal phase separation, thus supporting our conclusion that glycans are essential for phase separation. We have added this result (Figures 3F and 3G) and the related method in the revised manuscript (Lines 835-840).

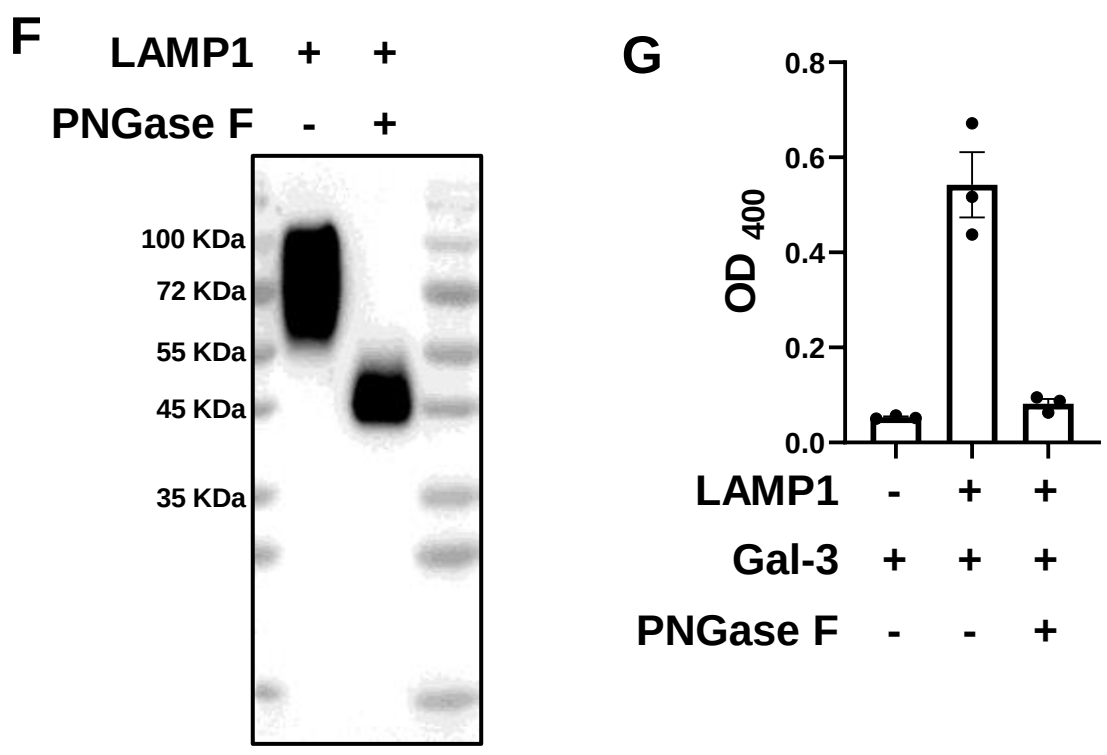

Figures 3F and 3G in the revised manuscript

• It is unclear why the Gal-3 CRD domain, insufficient for LLPS in vitro, still forms puncta in LLOMe-treated cells.

Response: Thank you for your question. Puncta at damaged lysosomes cannot be taken as evidence of LLPS in cells. Puncta derived from CRD do not have liquid-like or dynamic properties, clear proof of LLPS, in contrast to the puncta derived from full-length Gal-3. The reason that the CRD forms puncta is that the CRD is the lectin domain of the full-length Gal-3 responsible for glycan recognition and interaction, and thus, it can bind to the lysosomal glycoproteins like full-length Gal-3 in lysosome damaged cells. As a common explanation in the galectin field, the puncta of CRD represent pre-existing clusters of glycoproteins on damaged lysosome membranes or CRD mediated clustering of some glycoproteins (CRD-CRD interaction has been reported previously).

These glycoprotein clustering-derived puncta are relatively stable, unlike the dynamic puncta derived from Gal-3 condensation. Full-length Gal-3 also has a fraction of stable puncta, which is seen in our FRAP analysis that shows ~50% fluorescence was recovered after photobleaching (Figures 3M and 3N).

• Secretion assays again lack lysate controls.

Response: According to your suggestion, we have added lysate controls in the revised manuscript as shown in Figures 1, S2, S4, S5, 2, 3, 4, 8, S9, and S10.

Figure 4

• ALG-2 and DNAJC5 interactions are weakly supported by few peptides in pulldown; endogenous validation is required.

Response: To validate these interactions, we performed pull-down assays demonstrating that recombinant Gal-3 can precipitate endogenous ALG-2 and DNAJC5 from cell lysates. These results have been added to our revised manuscript in Figure 4B. Data of DNAJC5 are not shown here, because all data related to DNAJC5 have been removed from the revised manuscript.

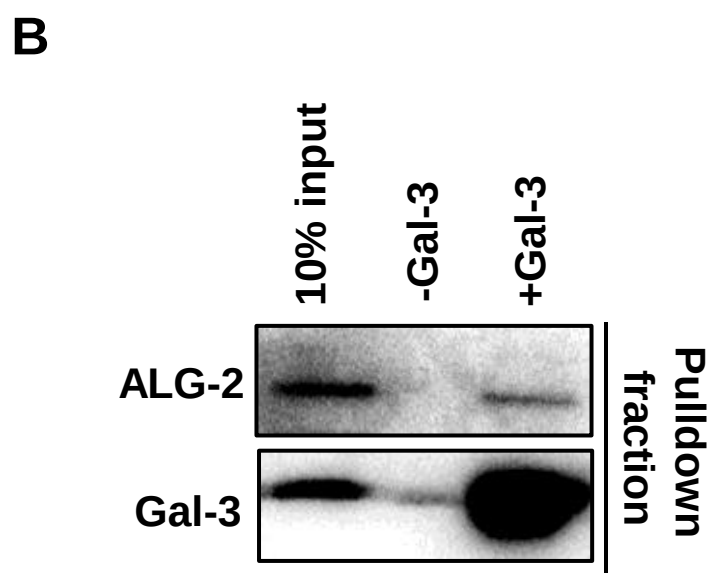

Figures 4B in the revised manuscript

• The text alternates between describing Gal-3 puncta as aggregates and LLPS, which is inconsistent.

Response: We have unified Gal-3 puncta and LLPS as Gal-3 condensates in the revised manuscript.

• Images in panel E are unconvincing; panel H lacks appropriate controls.

Pharmacological inhibitors of LLPS are not specific enough—genetic or biochemical evidence is needed.

Response: First, we have replaced images in panel E (Figure 4F in revised manuscript) with clearer, more representative ones. Second, we have added controls (LAMP1+Gal-3, LAMP1+ALG-2, and Gal-3+ALG-2) in panel H. Finally, we have introduced a key mutation (Gal-3 R186S) that does not undergo phase separation in the presence of LAMP1 and consequently loses the ability to recruit ALG-2. These results have been added to the revised manuscript in Figures 4F, 4I, and 4J.

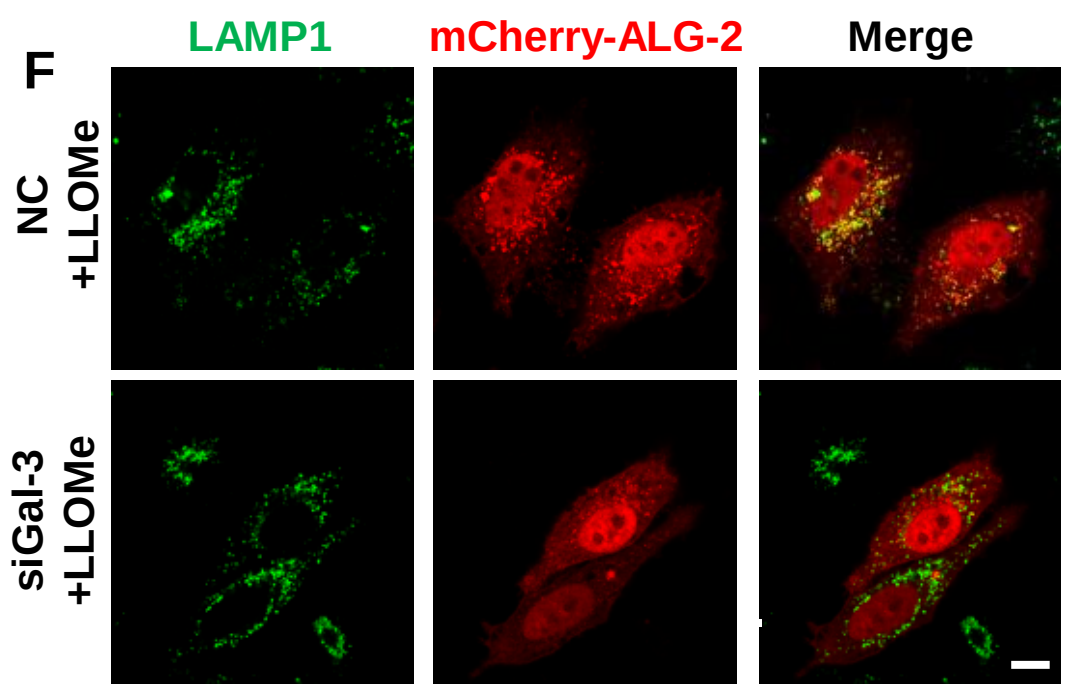

Figure 4F in the revised manuscript

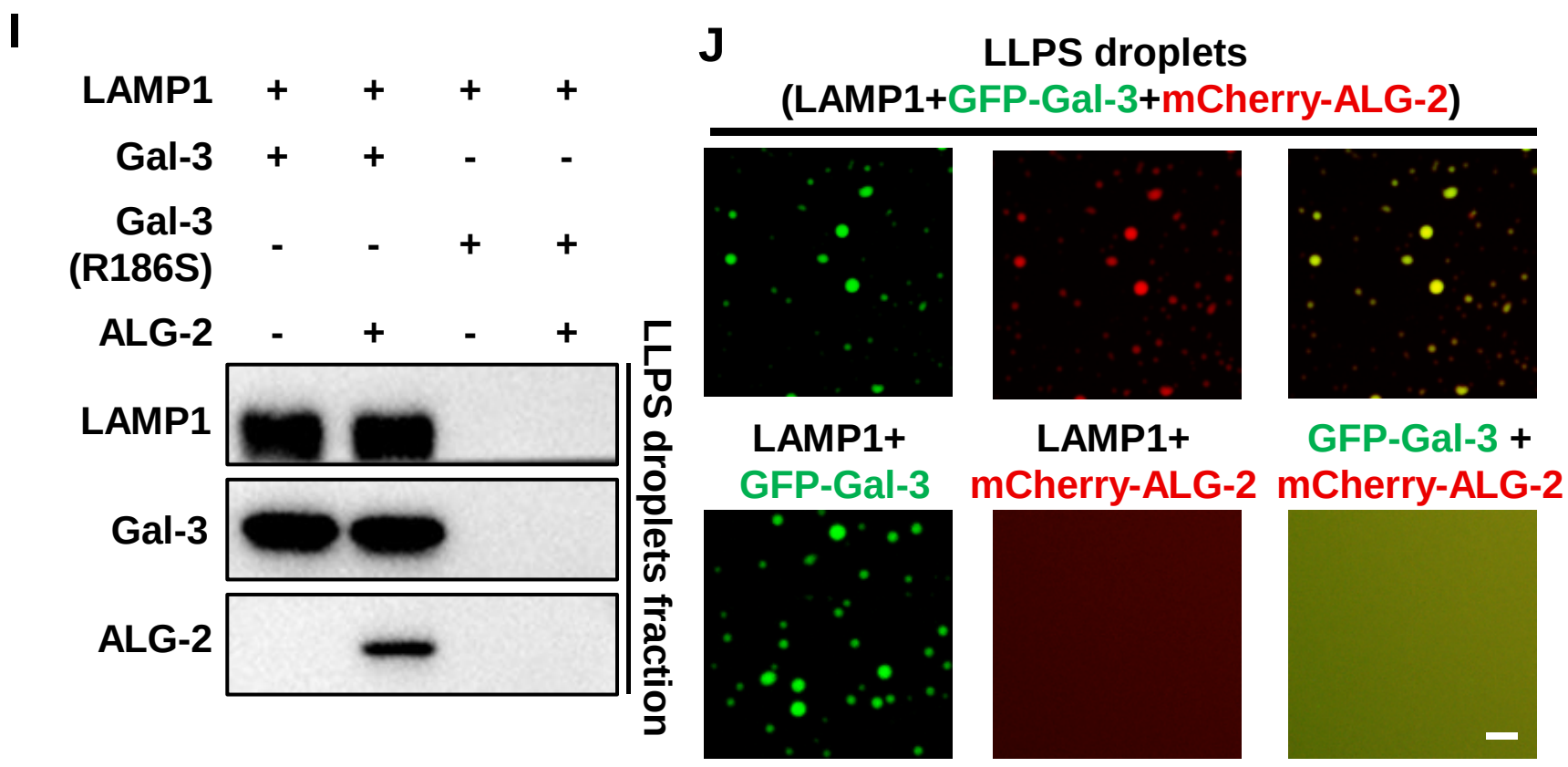

Figures 4I and 4J in the revised manuscript

Figure 5

• The proposed link between LLPS and ALG-2 recruitment is undermined by in vitro data showing ALG-2 binding without LLPS. This contradiction needs resolution.

Response: In our BLI assay, we loaded Gal-3 onto the Ni-NTA sensors either directly (via interaction of their His-tags with the Ni-NTA sensors) or indirectly (via interaction with the pre-immobilized LAMP1) (for clarity, we have added a schematic illustration

as shown in Figures 5B and 5C in the revised manuscript). We found that directly loaded full-length Gal-3 does not interact with ALG-2. However, when Gal-3 is indirectly loaded via LAMP1, it subsequently interacts with ALG-2. This indicates that LAMP1 binding is a prerequisite for the interaction between Gal-3 and ALG-2. Notably, glycoproteins immobilized on solid surface like-sensors and latex beads can induce Gal-3 phase separation as stated in our previous report[1]. In this regard, the data obtained from the indirect method reflect the interaction between Gal-3 condensates and ALG-2. This statement is further demonstrated with Ni-NTA beads and test tubes mimicking the process and rational of the indirect BLI assays. As shown in the following diagram, we loaded Gal-3 onto Ni-NTA beads via pre-immobilized LAMP1 and then placed the beads in test tubes containing ALG-2 with or without Gal-3, followed by detection of bead-associated Gal-3 and ALG-2. Our results show that the content of bead-associated Gal-3 is markedly reduced in the tube without Gal-3, an indication of the dynamic nature of bead-associated Gal-3. In this case, ALG-2 recruitment was also markedly impaired. Together, these results support our statement that ALG-2 association depends on Gal-3 condensates. These results have been added to our revised manuscript in Figure 5F.

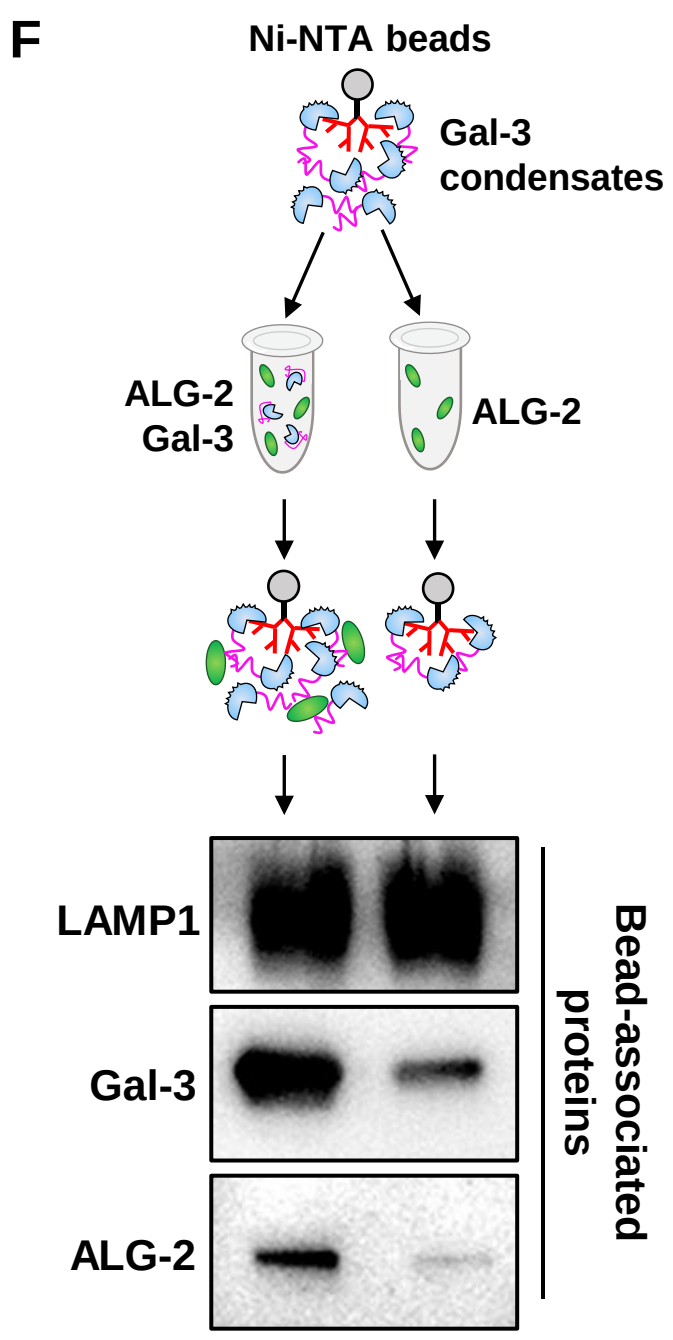

Figure 5F in the revised manuscript

1. Zhao Z, et al. Galectin-3 N-terminal tail prolines modulate cell activity and glycan-mediated oligomerization/phase separation. Proceedings of the National Academy of Sciences of the United States of America **118**, (2021).

• The reported affinity of ALG-2 for Gal-3 (Kd ~250 μM) is very weak and conflicts with the N-domain binding data. Could synthetic glycans improve affinity?

Response: We have addressed this issue from three perspectives.

(1) Inhibitory effect of CRD: ALG-2 binding sites reside on the NT domain of Gal-3. In full-length Gal-3, the NT transiently interacts with the F-face of the CRD[1], masking the ALG-2 binding sites. Upon binding to glycans, conformational changes in the CRD alter this CRD-NT equilibrium, favoring an "NT-free" state, which exposes the ALG-2 binding region and facilitates interaction between the NT and ALG-2 (Figure 5J). However, unlike the NT peptide that is totally free, the NT domain in full-length Gal-3 remains "constrained" to some extent by the CRD even though the equilibrium is shifted toward the "NT-free" state.

(2) Differences in coating method: In the BLI assay, NT was directly coated onto the sensor, whereas full-length Gal-3 was coated by pre-immobilized LAMP1 (directly coated Gal-3 did not bind ALG-2). This difference in coating strategies likely results in a higher surface density of NT peptide compared to the density of free NT region derived from LAMP1-associated Gal-3. A higher coating density of NT would have more binding sites for ALG-2, thus leading to apparent stronger affinity.

(3) Difference of NT orientation and structural arrangement: In the direct loading BLI approach, NTs were coated onto the Ni-NTA sensor via His-tags. This site-specific immobilization may promote a more favorable alignment or orientation of the NT domain, thus facilitating a more efficient interaction with ALG-2. In contrast, Gal-3 coated by pre-immobilized LAMP1 exists in the form of condensates. Transient interactions between NT and CRD within full length Gal-3 condensates may lead to a disordered molecular arrangement that is less conducive to ALG-2 binding.

Overall, this may explain why the affinity for full-length Gal-3 is weaker than that of the NT peptide.

Additionally, we agree with your speculation that synthetic glycans could improve affinity. We have experimental data showing synthetic glycans improve ALG-2 binding to full-length Gal-3. To avoid confusion resulting for readers, we have not included these data. In fact, the affinity of ALG-2 for full-length Gal-3 (K_D ~250 μM) was obtained in the presence of glycoprotein LAMP1 (Figure 5E). Without LAMP1, their interaction was undetectable (Figure 5D).

1. Ippel H, et al. Intra- and intermolecular interactions of human galectin-3: assessment by full-assignment-based NMR. Glycobiology **26**, 888-903 (2016).

• could AlphaFold modeling strengthen the analysis, followed by biochemical validation.

Response: Thank you for your suggestion. We have performed AlphaFold modeling for the interaction between ALG-2 and Gal-3. However, as shown in the following figures, we found that all predicted models exhibited low confidence and high variability. The interface precision scores (ipTM) were consistently low (ranging between approximately 0.28–0.51) and fluctuated significantly from run to run. This may be due to the following reasons: 1) Gal-3 contains intrinsically disordered regions in the NT domain; 2) Gal-3 has transient intramolecular interactions between the NT and CRD domains; 3) the interaction between glycan-activated Gal-3 and ALG-2 is relatively weak (K_D = 246.10 ± 63.80 μM). Only Gal-3 condensates/assemblies that contain multiple molecules and thus multivalency could recruit ALG-2 (please see the new Figure 5F).

Furthermore, in both LLPS droplet and BLI assays, Gal-3 did not interact with ALG-2 unless glycoproteins were present. Glycoprotein binding resulted in Gal-3 condensate formation and, simultaneously, the release of the NT domain that is necessary for ALG-2 binding. To avoid confusion for the readers, we decided not to present the AlphaFold modeling data in the manuscript.

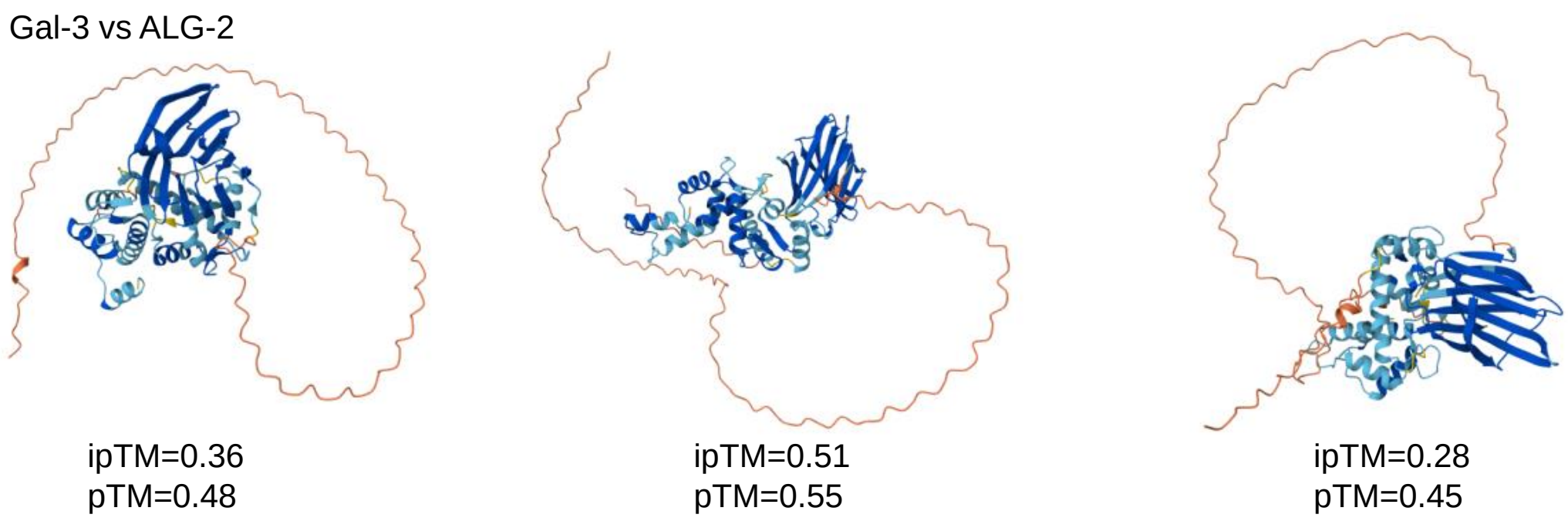

Figure AlphaFold modeling for the interaction between ALG-2 and Gal-3

• Figure 5H is preliminary; proper Kd measurements for mutants are needed.

Response: We determined the K_D for key NT mutants (P17A, P30A, P46A, P50A, P51A,

P55A) identified in our initial screening. These results show that the binding affinities of these mutants are weakened compared to WT. These results have been added to our revised manuscript in Figure S8.

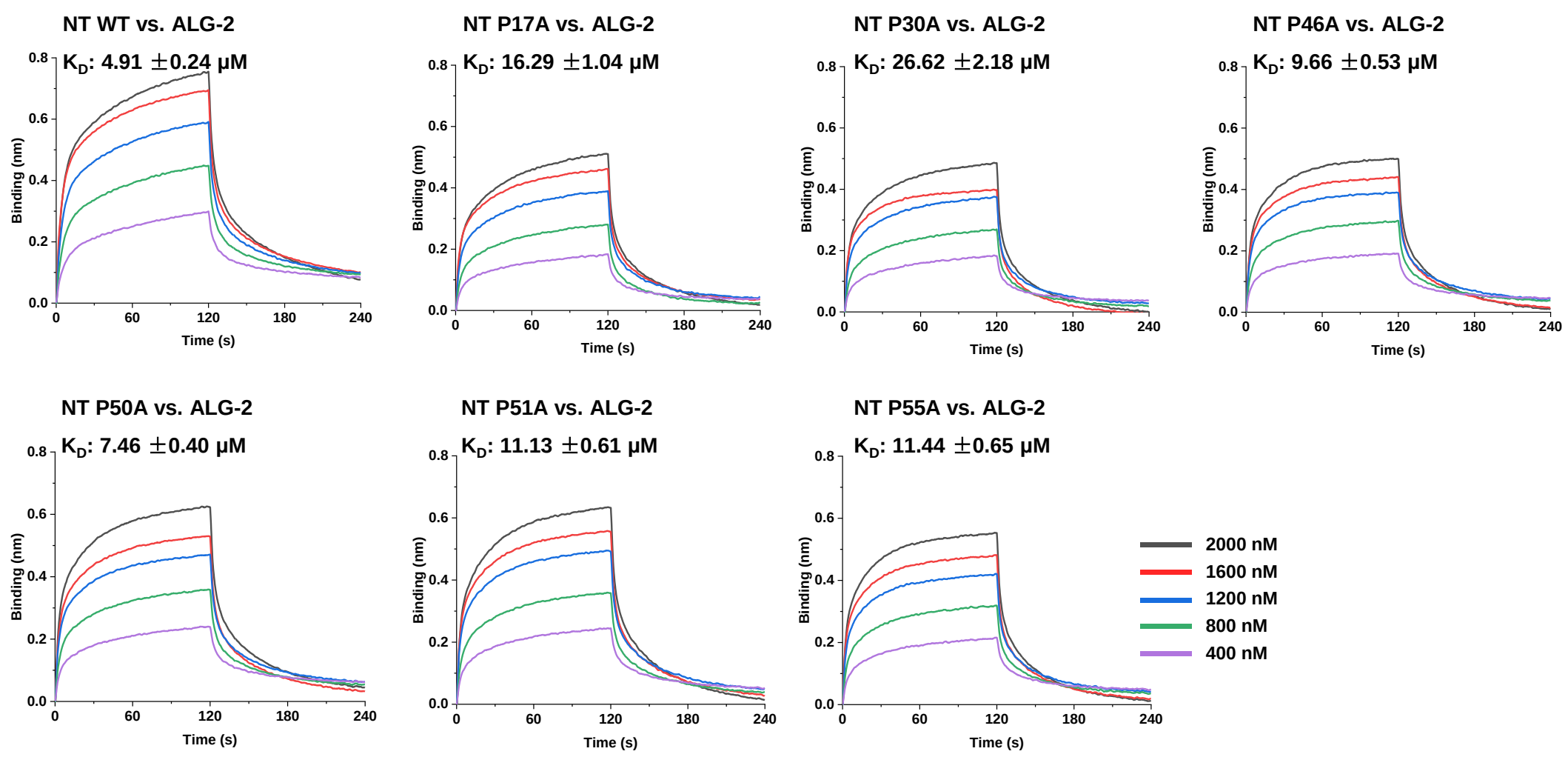

Figures S8 in the revised manuscript

• All purified proteins should be shown on a gel.

Response: We have performed SDS-PAGE analysis for all purified proteins, including the Gal-3 variants, Gal-3 NT proline mutants, and ALG-2 variants. These results have been added to our revised manuscript in Figure S11.

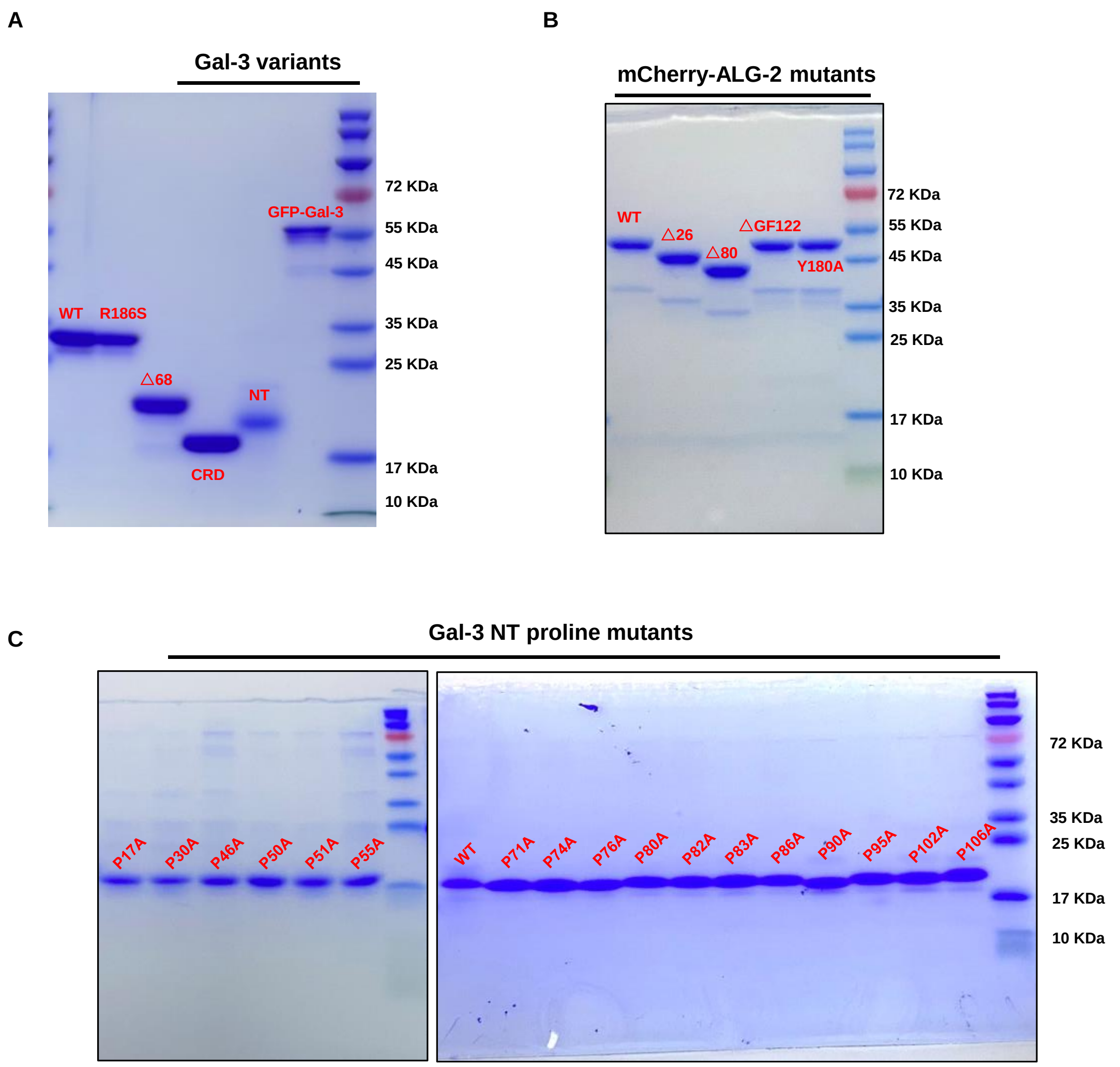

Figures S11 in the revised manuscript

• The claim that Gal-3 translocates to the ER contradicts earlier data (Figure 2) showing localization to damaged lysosomes. The discrepancy is not explained.

Response: Thank you for raising this question. Here is our explanation. Recent studies have indicated that damaged lysosomes rapidly initiate repair machinery and reposition themselves in close proximity to the ER for lipid exchange and repair[1]. Additionally, our results suggest that ALG-2 directs Gal-3 condensates to the ER (Figures 7B and 7C). Thus, it is not surprising that Gal-3 concurrently co-localizes with damaged lysosomes (where condensation is initiated) and ER (where lysosomal repair occurs). This analysis is reflected in our revised model (Figure 9).

1. Tan JX, Finkel T. A phosphoinositide signalling pathway mediates rapid lysosomal repair. Nature **609**, 815-821 (2022).

Figure 7

• The inclusion of DNAJC5 in Gal-3 LLPS is interesting but currently underdeveloped. This part suffers the same caveats as listed above. This section could be removed to focus on strengthening the main findings.

Response: Thank you for your suggestion. We have removed this part to focus on strengthening our main findings.

Summary

The proposed mechanism linking Gal-3 secretion, glycoprotein binding, and LLPS is conceptually exciting, but the current dataset has major weaknesses. The manuscript requires substantial experimental reinforcement, particularly with rigorous controls, clear logic, and stronger validation of key interactions, before it can be considered for publication in Nature Communications.

Response: We appreciate the positive assessment of our proposed mechanism's novelty and the constructive feedback from you. In our revised manuscript, we have undertaken a comprehensive experimental effort to address the concerns regarding rigorous controls and validation. We believe that our new data provide compelling support for our model.

Reviewer #2 (Remarks to the Author):

In this manuscript Zhao et al put together a compelling story that Gal-3 is secreted by the UPS pathway during phase separation. Using a combination of cell biology, microscopy, and biophysics they demonstrate that Gal-3 interacts with N-glycan on lysosomal proteins of to mediate this secretion. This was further demonstrated to be through interactions with the NT domain of Gal-3 as Gal-3 NT KO variants did not demonstrate secretion or phase separation. Overall I think this manuscript represents a strong contribution to the field and would of interest to a broad readership.

Some suggestions on how this manuscript could be improved include

1.) chemical and genetic manipulation of glycosylation was used to implicate hybrid and complex N-glycans, however this analysis does not specify which glycoforms are driving these interactions and if the glycoproteins implicated (e.g. lamp1) is known to have these glycans.

Response: Thank you for your comment. To address this, we performed enzymatic digestion experiments using enzymes PNGase F and Endo H. PNGase F removes all N-glycans, whereas Endo H specifically hydrolyzes high-mannose and hybrid-type N-glycans, but not complex N-glycans[1, 2].

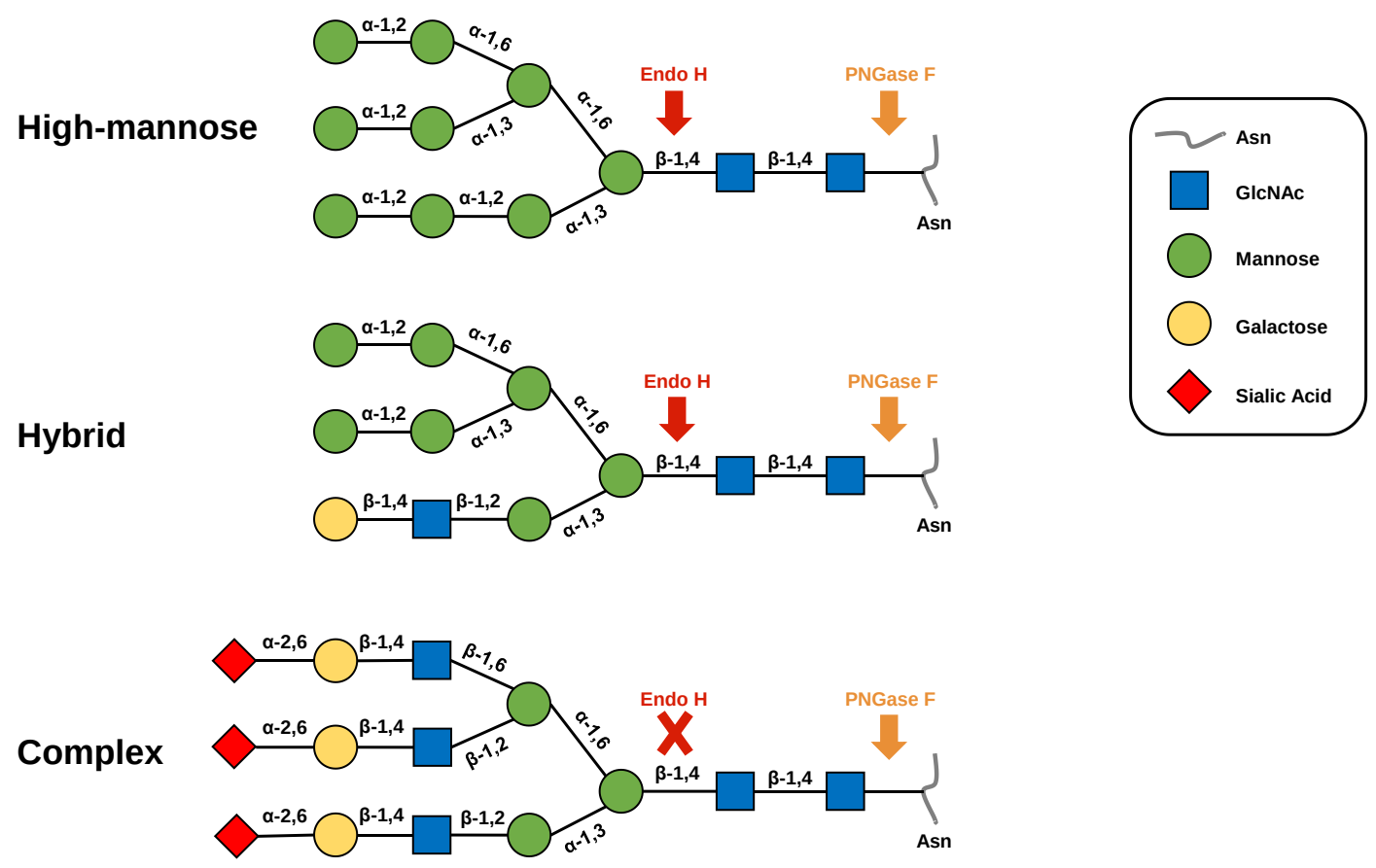

Figure S7A in the revised manuscript

Treatment of LAMP1 with PNGase F resulted in a substantial molecular weight shift and, crucially, a severe loss of its ability to induce Gal-3 phase separation. In contrast, Endo H treatment caused only a minimal molecular weight change and, importantly, did not impair LAMP1's function in driving Gal-3 phase separation. These results provide evidence that complex N-glycans in LAMP1 are essential for inducing Gal-3 phase separation. These results and relevant methods have been added to our revised manuscript in Figures 3 and S7.

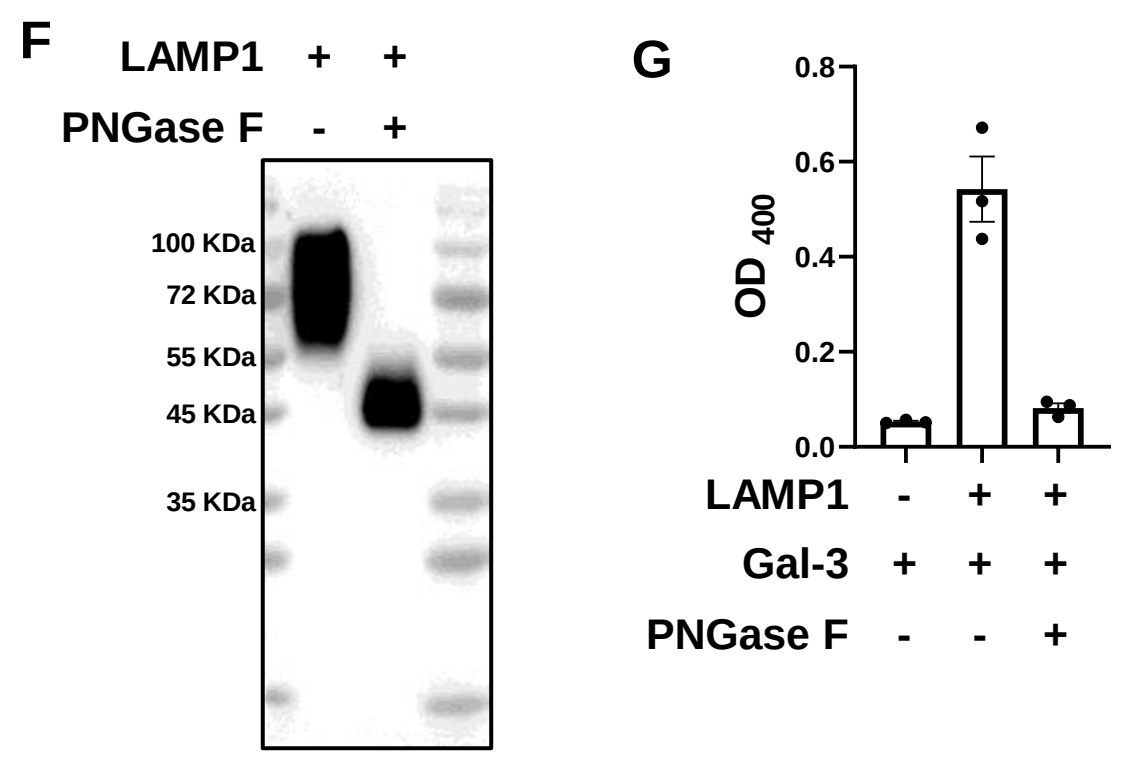

Figures 3F and G in the revised manuscript

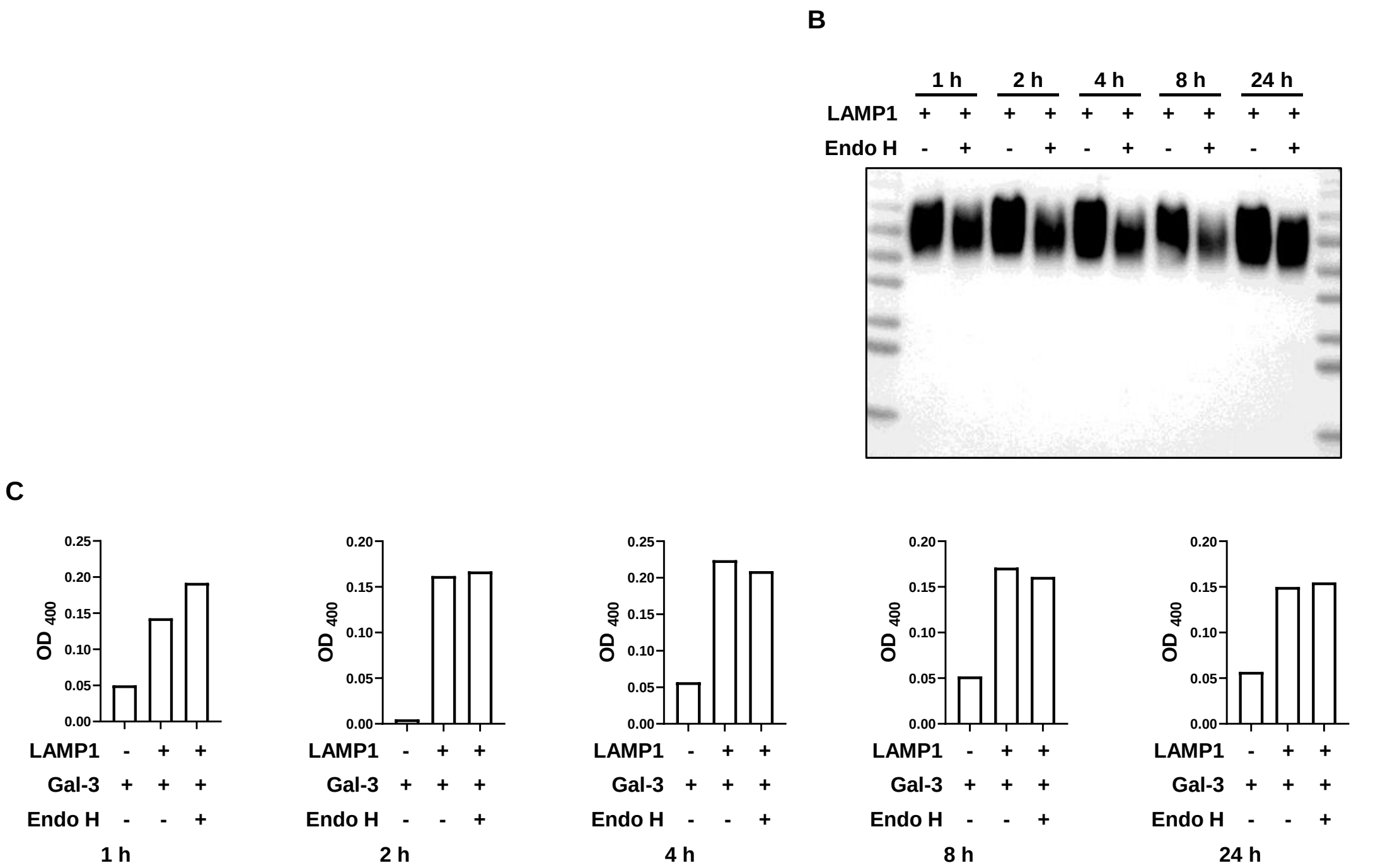

Figures S7B and S7C in the revised manuscript

1. Cao L, et al. Global site-specific analysis of glycoprotein N-glycan processing. Nature protocols **13**, 1196-1212 (2018).

2. Mukherjee MM, et al. Selective bioorthogonal probe for N-glycan hybrid structures. Nat Chem Biol **21**, 681-692 (2025).

2.) in vitro experiments could be bolstered by PNGaseF treatment of proteins to demonstrate that LLPS formation is N-glycan dependent in vitro.

Response: We have performed a PNGase F digestion to remove N-glycans on LAMP1 and then assessed its ability to induce phase separation. These results show that PNGase F-treated LAMP1 induces minimal phase separation, thus supporting our conclusion that *N*-glycan is essential for phase separation. We have added this result (Figures 3F and 3G) and the related method in the revised manuscript (Lines 835-840).

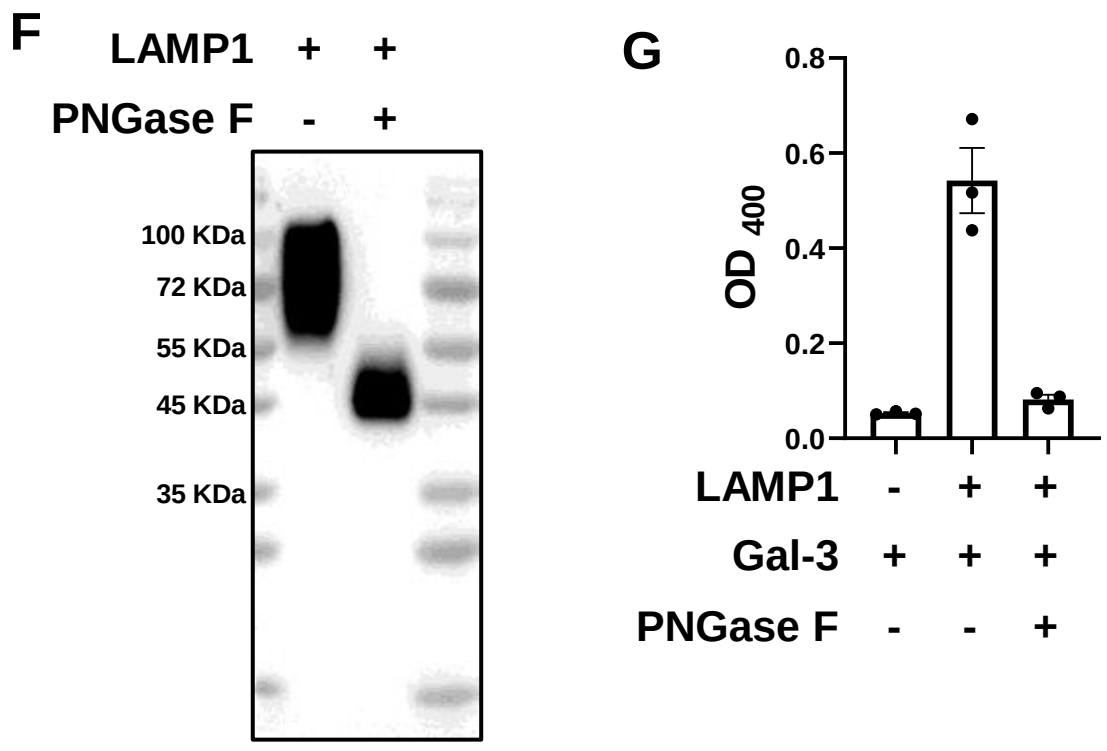

Figures 3F and 3G in the revised manuscript

3.) figure 5 BLI data is difficult to follow what is present on each surface, maybe use a pictoral representation to describe the complexes on each BLI tip surface

Response: Thank you for your suggestion. We have added schematic diagrams in Figure 5 in revised manuscript.

4.) contextualization of Gal-3 pahse separation in the stickers and linkers model will help undertand the molecular basis of these interactions.

Response: Thank you for your suggestion. In our revised manuscript, we have contextualized Gal-3 phase separation within the "stickers and linkers" model. As suggested, we describe the NT of Gal-3 as the key multivalent sticker that drives phase separation. The CRD, in this framework, functions as a critical spacer/linker, providing the structural flexibility and valency necessary for dynamic condensate formation[1]. We have added this model to Figure S6 as shown below.

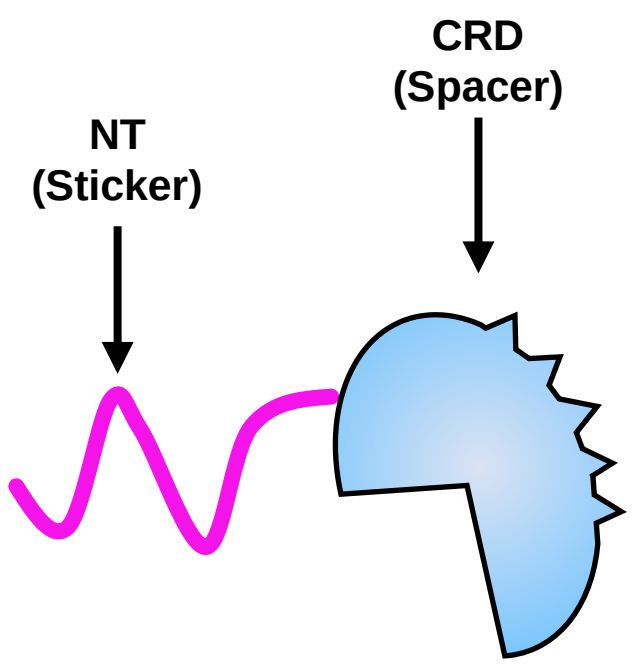

Figure S6 in the revised manuscript

1. Zhao Z, et al. Galectin-3 N-terminal tail prolines modulate cell activity and glycan-

mediated oligomerization/phase separation. Proceedings of the National Academy of Sciences of the United States of America **118**, (2021).

5.) Figure 4A, please indicate which bands are which protein (or indicate each proteins molecular weight) on the silver stained pulled down gel.

Response: We have labeled the proteins corresponding to the relevant bands.

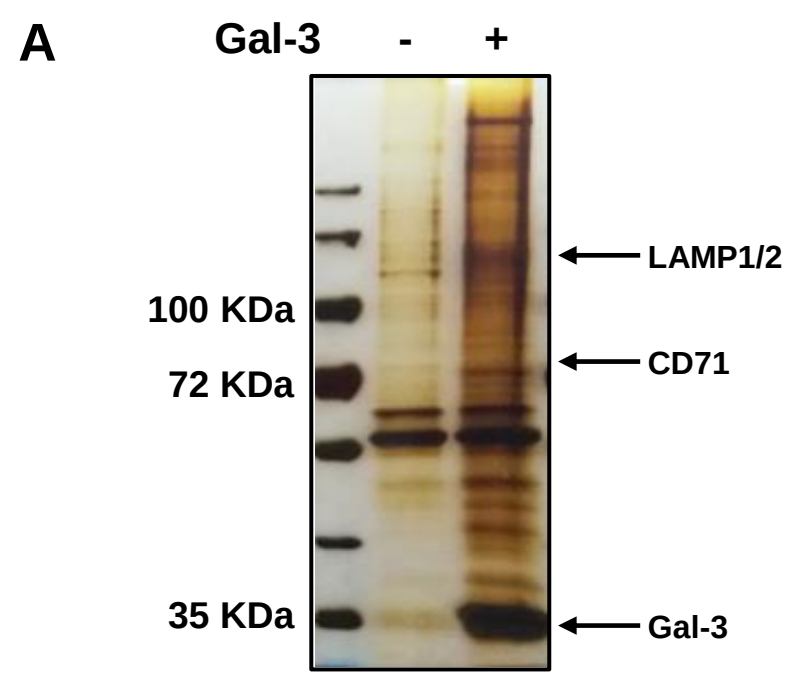

Figure 4A in the revised manuscript

Reviewer #3 (Remarks to the Author):

In this manuscript, the authors describe that Galectin-3 (Gal-3) undergoes an unconventional protein secretion pathway driven by glycoprotein-induced phase separation, leading to the formation of large non-exosomal aggregates independent of pyroptosis or secretory autophagy. These aggregates recruit ALG-2 and DNAJC5, which guide them from the ER–late endosome interface into late endosomes. Finally, Gal-3 is exported into the extracellular space via SNARE-dependent vesicular transport, acting as a damage-associated “clean-up” mechanism.

I find the idea of glycoprotein-induced phase separation convincing, as it makes conceptual sense. However, my main concern lies in the proposed topology. LAMP1, LAMP2, and CD71 are transmembrane proteins, and it is difficult to understand how such membrane-anchored proteins could be incorporated into condensates without simultaneously bringing along their associated lipid bilayer. Similarly, it is unclear how a protein condensate would enter late endosomes without some form of invagination process, which would necessarily enclose the condensate within a membrane. These are fundamental principles of membrane biology that generally cannot be violated.

A more plausible alternative model is that Gal-3 enters damaged lysosomes, where it forms condensates with glycoproteins on the luminal side of the lysosomal membrane. These damaged lysosomes could then directly, or indirectly via late endosomes, release the condensates into the extracellular space through fusion with the plasma membrane. I recommend that the authors consider such an alternative model, which may be more consistent with established principles of membrane biology.

Response: Thank you for your insightful comment. We have carefully considered the points you raised and have revised our model accordingly.

1) Regarding the membrane-anchored proteins

We agree with your comment that transmembrane glycoproteins such as LAMP1, LAMP2, and CD71, would not be expected to enter condensates without membrane fragments. Accordingly, we have revised our model of Gal-3 secretion as such: Gal-3 enters damaged lysosomes, where it forms condensates with glycoproteins on the luminal side of the lysosomal membrane (step (1) in the revised model). When the condensates are translocated into the LE, glycoproteins in the condensates remain attached to a piece of membrane (step (4) and (5) in the revised model). This is plausible given that a protein complex (ESCRT-III) has been found to "cut" the damaged membrane and facilitate cytoplasmic membrane repair[1,2] which could allow membrane-embedded glycoproteins to become associated with cytosolic condensates.

2) Regarding the organelle mediating Gal-3 vesicular transport

Although we cannot exclude the possibility of direct fusion between lysosome and plasma membrane, our current data suggest that Gal-3 condensates are secreted via LE-mediated vesicular transport, because the secretion depends on LE localization and Rab7a, a key function protein in LE-mediated vesicular transport (Figures 7F–7H and 8A).

3) Regarding the pathway for Gal-3 entry into LEs

a) As you correctly note, invagination is a well-known mechanism implicated in translocating cytosolic proteins or aggregates into membranous compartments. However, this mechanism seems unlikely, because invagination of Gal-3 condensates would result in membrane-enclosed Gal-3 vesicles within a membranous compartment (i.e., multivesicular bodies), leading to exosomes release. This would be contradictory to our results (Figure 1O).

b) Instead, transmembrane translocation seems more likely. It was reported recently that

certain unconventionally secreted cytosolic proteins can access membrane-bound compartments via translocation mechanisms involving transient pore formation by membrane-associated factors (i.e., TMED10 and DNAJC5)[3,4]. DNAJC5 has been implicated in mediating α-synuclein (an UPS protein) translocation from the cytoplasm to the luminal side of LE[4]. Interestingly, our preliminary data have shown that knocking down DNAJC5 (but not TMED10) significantly inhibits Gal-3 secretion, suggesting a potential role for DNAJC5 in facilitating Gal-3 entry into LEs. In any event, we have removed these data on DNAJC5 as recommended by Reviewer #1, as further development is required.

c) Lysosome–LE fusion occurs during endo-lysosomal trafficking and organelle maturation. This fusion process may represent an additional route through which Gal-3 condensates are delivered into membrane-enclosed compartments.

We have added these discussions in the revised manuscript (Lines 553-566).

Based on your suggestions and our data, we have revised our model as shown below. (1) Exposed glycoproteins within damaged lysosomes bind and recruit cytosolic Gal-3. Glycoprotein binding induces conformational changes in the F-face of the Gal-3 CRD, leading to Gal-3 NT release and condensation. (2) Gal-3 molecules within these condensates use their “free” NTs to recruit ALG-2. (3) ALG-2 directs the condensates to the interface between the ER and LEs. (4) The Gal-3 condensates are translocated into the LEs. (5) Finally, as “cargo,” Gal-3 condensates within LEs are delivered to the plasma membrane and released into the extracellular milieu by SNARE-dependent vesicular transport.

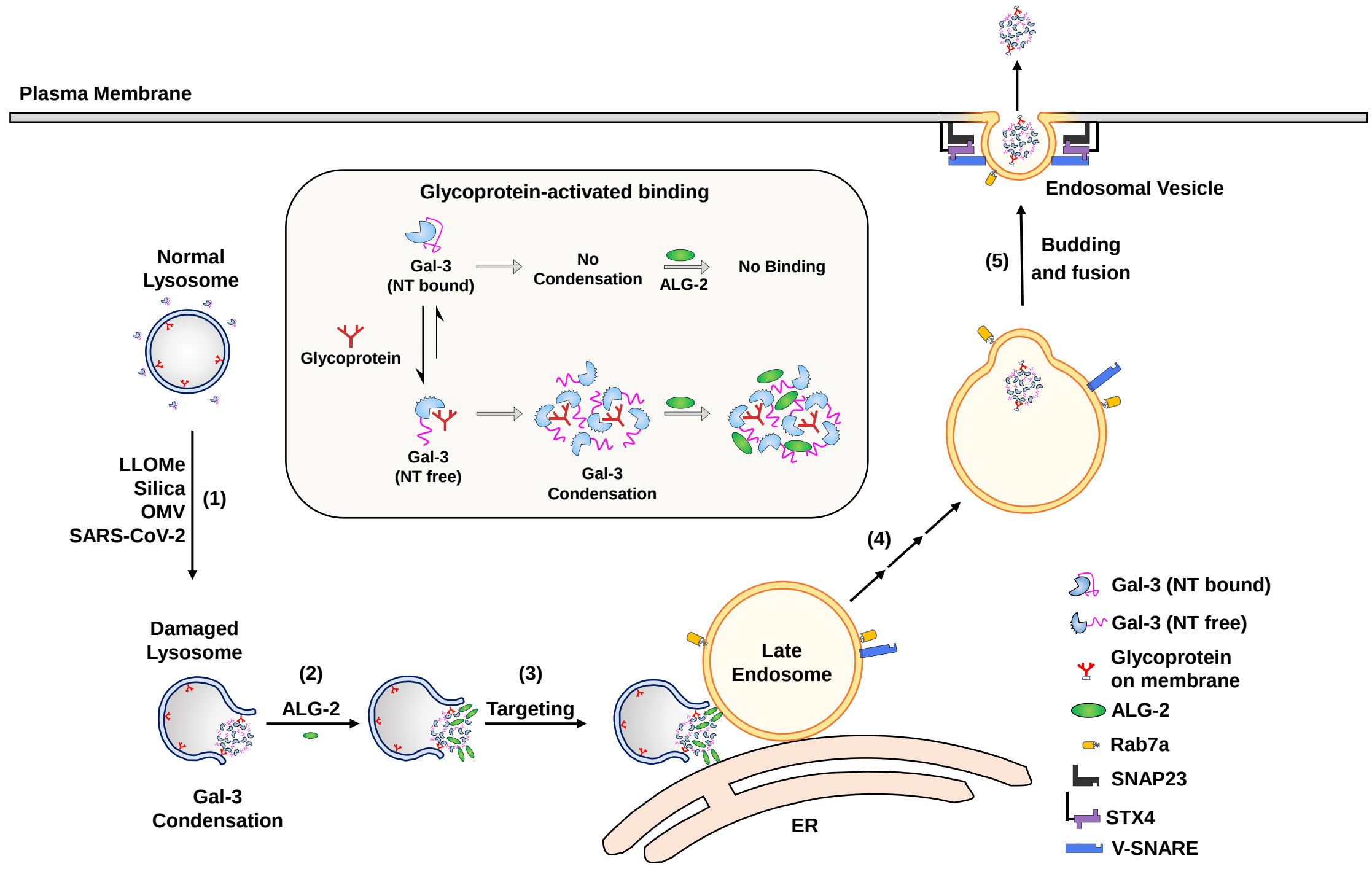

Figure 9 in the revised manuscript

1. Cada AK, et al. Friction-driven membrane scission by the human ESCRT-III proteins CHMP1B and IST1. Proceedings of the National Academy of Sciences of the United States of America **119**, e2204536119 (2022).

2. Jimenez AJ, Maiuri P, Lafaurie-Janvore J, Divoux S, Piel M, Perez F. ESCRT machinery is required for plasma membrane repair. Science **343**, 1247136 (2014).

3. Zhang M, et al. A translocation pathway for vesicle-mediated unconventional protein secretion. Cell **181**, 637-652.e615 (2020).

4. Wu S, Hernandez Villegas NC, Sirkis DW, Thomas-Wright I, Wade-Martins R, Schekman R. Unconventional secretion of α-synuclein mediated by palmitoylated DNAJC5 oligomers. eLife **12**, (2023).

REVIEWER COMMENTS

Reviewer #4 (Remarks to the Author):

This is an intriguing study demonstrating that the phase separation of Gal3, mediated by its binding to glycans exposed upon lysosomal damage, facilitates the UcPS (unconventional protein secretion) of Gal3. Overall, the authors have presented a substantial amount of data in the revised version. However, I have a few additional suggestions that could enhance the work:

1. TMED10 Involvement: Multiple studies have shown that TMED10 regulates the release of galectins, including Gal1, Gal3, and Gal9 (PMID: 38926505; PMID: 32272059; PMID: 40335523; PMID: 41364076). It would be beneficial to investigate the role of TMED10 in lysosomal damage-related systems and its potential involvement in Gal3 secretion.

Response: Thank you for your comments. We have performed the RNAi of TMED10 in a lysosomal-damage model. As shown in the figure below, knocking down TMED10 has no effect on Gal-3 secretion, indicating that TMED10 is not involved in lysosomal damage-triggered Gal-3 secretion. This result further demonstrates that the enhanced Gal-3 secretion upon lysosomal damage differs from the constitutive secretion from cells with normal lysosomes. We have added this result in our revised manuscript (lines 553–558, Figure S11).

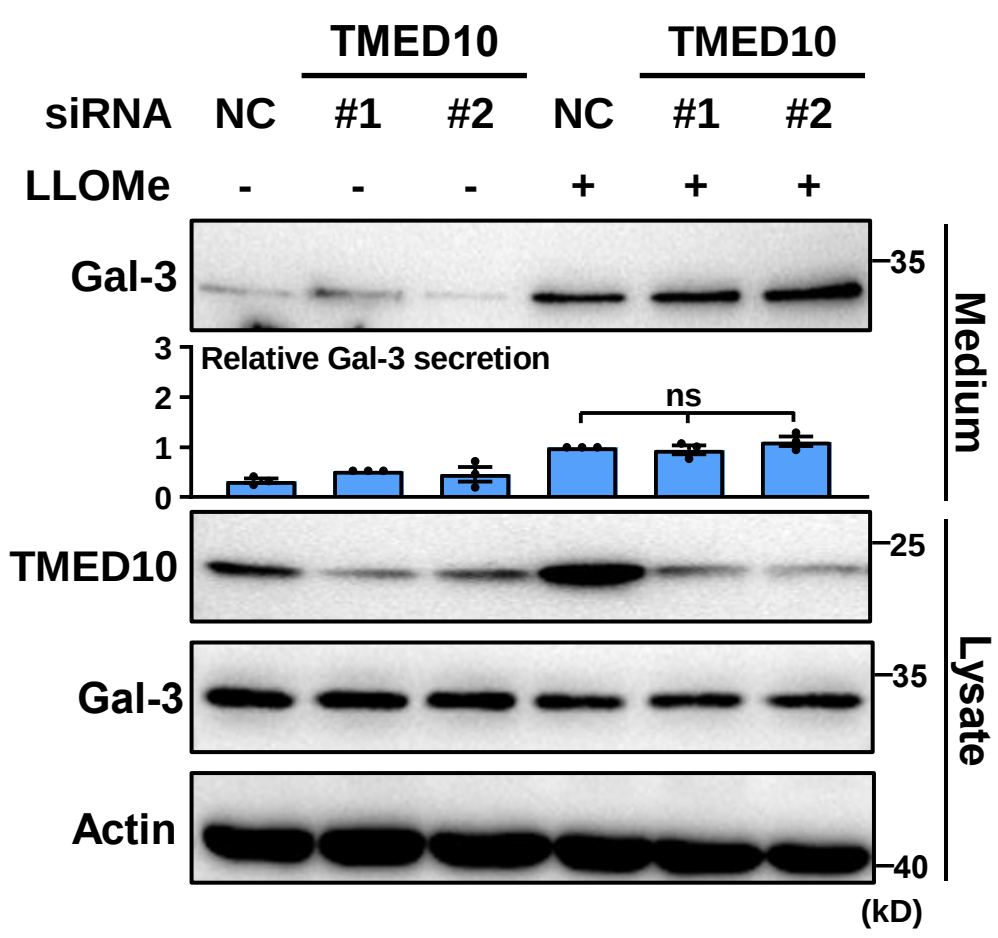

Figure S11A in the revised manuscript

2. ESCRT III Components: Given that ESCRTs are involved in lysosome repair, it would be valuable to test the involvement of ESCRT III components in Gal3 secretion

to further validate the proposed model.

Response: Your thoughtful suggestion precisely addresses the key mechanistic question of how Gal-3 condensates are detached from damaged lysosomal membranes. This is indeed a fundamental and intriguing issue that is essential to fully elucidate the unconventional secretion pathway we proposed, and we highly agree that it deserves in-depth investigation. This important point has become a major focus of our follow-up work, and we have already obtained relevant preliminary experimental data at this stage.

The ESCRT III complex consists of a series of core components including CHMP1-7 and IST1, which are primarily responsible for membrane scission and lysosomal membrane repair. To investigate whether ESCRT III participates in Gal-3 secretion, we initially focused on CHMP4B, a core ESCRT III subunit that has been demonstrated to be recruited to damaged lysosomes in a Gal-3-dependent manner. The figure below shows that knockdown of CHMP4B does not significantly affect Gal-3 secretion under our experimental conditions, indicating that CHMP4B is not involved in Gal-3 secretion.

Nevertheless, we cannot rule out the possibility that other ESCRT III homologs (e.g., CHMP2, CHMP7, IST1) may participate in the process of membrane scission. We are now systematically investigating other ESCRT III components.

[FIGURE REDACTED]

Figure for Reviewer only (unpublished data)

3. ERGIC Localization: The ERGIC localization of Gal3 should also be tested in the context of lysosomal damage, alongside the ER and lysosomal markers already presented in the paper.

Response: To address this point, we performed colocalization analysis between Gal-3 and the classic ERGIC marker ERGIC53 under lysosomal damage conditions. Our results showed no obvious colocalization between Gal-3 and ERGIC53 (Figure S11B), suggesting that lysosomal damage-induced Gal-3 secretion is not through ERGIC. Given that ERGIC is a critical intermediate compartment for the TMED10-channelled UcPS, the independence of ERGIC observed here is in line with the independence of TMED10 observed in response to comment 1 (Figure S11A). Overall, these data support our statement that lysosomal damage-induced Gal-3 secretion occurs via a previously uncharacterized secretory pathway independent of the ERGIC and TMED10-mediated UcPS. We have added these results in the revised manuscript (lines 553–558, Figure S11).

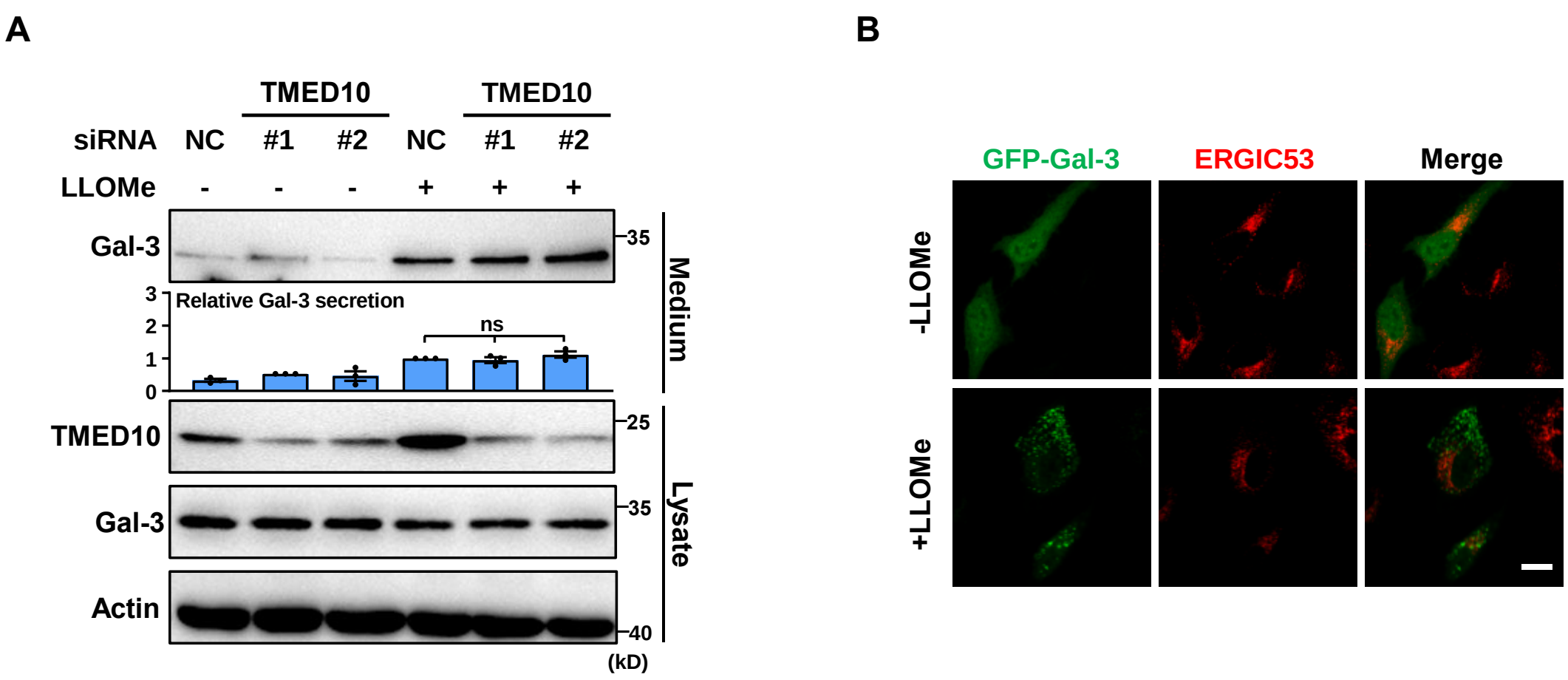

Figure S11 in the revised manuscript

4. ER-Lysosome Contact: Is the ER-lysosome contact essential for Gal3 secretion, or is it merely coincidental? RNAi of VAPA could help determine whether this contact is required for Gal3 secretion.

Response: As you suggested, VAPA knockdown would indeed provide direct evidence to clarify the functional importance of ER-lysosome contact in regulating Gal-3 secretion and would substantially deepen our mechanistic understanding of this unconventional secretory pathway. We fully agree that this is a fascinating and biologically meaningful topic, and we will prioritize it in our future investigations. Considering the limited time frame for manuscript revision, it is impractical for us to complete the relevant VAPA knockdown experiments within the current revision cycle. Instead, we have provided our unpublished ATG2A-related data here, which is closely

relevant to your question.

ATG2A has been reported to mediate lipid transfer from the ER to the damaged lysosome for membrane repair (PMID: 36071159). Although direct evidence for its role in bridging ER and lysosome is not available, ATG2A has been shown to tether the ER to phagophore (PMID: 31271352). Our unpublished data showed that knocking down ATG2A significantly reduced Gal-3 secretion (please see the figure below). This result suggests that ER-lysosome contacts might be involved in Gal-3 secretion, by providing a specialized microenvironment for efficient lipid transfer and subsequent Gal-3 secretion. However, this hypothesis remains to be further verified. We are now engaged in knockdown VAPA to disrupt ER-lysosome contact and further clarify its role in Gal-3 secretion.

[FIGURE REDACTED]

Figure for Reviewer only (unpublished data)

5. Molecular Weight Markers in WB: All molecular weight markers should be clearly labeled in Western blot gels for better clarity.

Response: We have labeled all molecular weight markers in Western blot gels of the revised manuscript.

6. Figure 2 - Glycoprotein Depletion: In Figure 2, numerous glycoproteins are capable of binding Gal3. Why does depleting just one, such as LAMP1, affect Gal3 secretion? There should be other glycoproteins that can still bind Gal3 and facilitate its release.

Response: While Gal-3 can indeed bind various glycoproteins, we propose that the

Gal-3 secretion is sensitive to local density of N-glycans, particularly that provided by highly abundant lysosomal glycoproteins such as LAMP1, LAMP2, and CD71. Regarding the result of knocking down each glycoprotein, our explanation is as follows.

LAMP1, LAMP2, and CD71 are highly abundant lysosome glycoproteins, that form the primary scaffold of the endomembrane glycocalyx to recruit Gal-3. Depleting any one of these glycoproteins leads to a local reduction in overall glycan density on damaged membranes. This compromised glycan platform is less efficient at recruiting Gal-3, thereby impairing phase separation that is essential for secretion. Indeed, our previous data indicated that Gal-3 phase separation is dependent on glycoprotein concentration (PMID: 33952698), i.e., when the concentration of Gal-3 in the surroundings is fixed, the ability of Gal-3 phase separation is proportional to the concentration of glycoproteins.

We propose that removing a highly abundant glycoprotein like LAMP1 breaks the glycan network, reducing the glycan density on damaged membranes. Even if other glycoproteins are present, the local concentration of glycan may fall below the critical threshold required to form or maintain the Gal-3 condensates that are essential for secretion.

7. Figure 1C - Gal3 Decrease: In Figure 1C, there is a noticeable decrease in Gal3 in the medium after 4 hours. Could you provide more insight into the reason for this reduction?

Response: In our experimental design, HeLa cells were treated with 1 mM LLOMe for 30 min, followed by a 12 h incubation in SFM. The conditioned medium was sequentially collected from and fresh medium added to the same cell population in three intervals: 0–4 h, 4–8 h, and 8–12 h. For readers convenience, we have added a schematic diagram to Figure 1C in the revised manuscript.

As shown in Figure 1C, Gal-3 secretion mainly occurs within the first 4 hours, which is directly triggered by lysosomal damage. As widely reported that damaged lysosomes undergo rapid and efficient membrane repair, our data also showed that damaged lysosomes are almost fully repaired after 4 hours (please see the figure below). Gal-3 secretion is low under normal lysosomes, which explains why Gal-3 level is decreased after 4 hours.

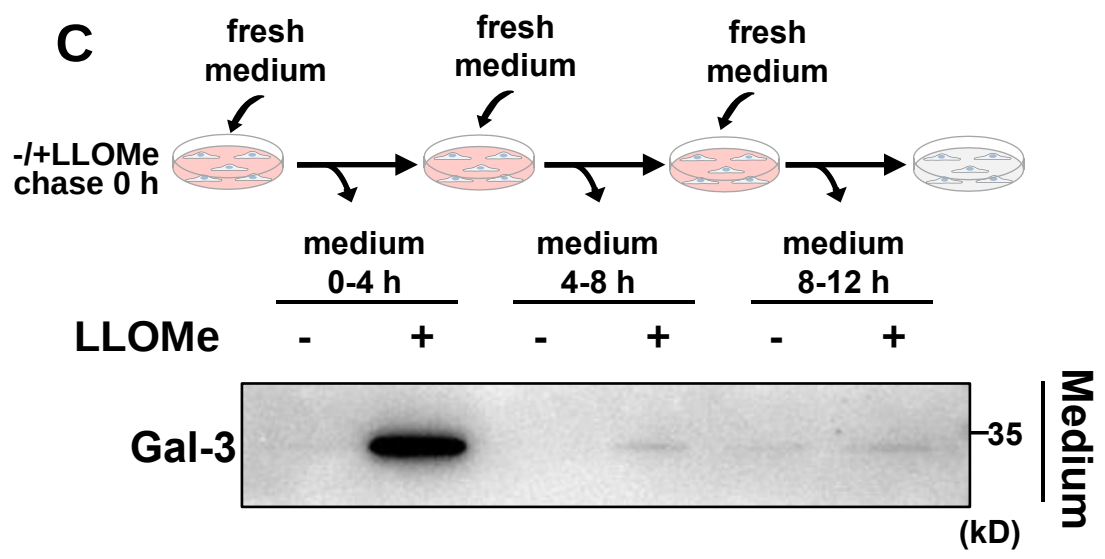

Figure 1C in the revised manuscript

[FIGURE REDACTED]

Figure for Reviewer only (unpublished data)

8. Line 40 - Terminology Update: The term "exosome-mediated release" should be updated to "extracellular vesicle-mediated release" since exosomes represent only a subclass of these vesicles.

Response: We have updated the term to "extracellular vesicle-mediated release" in the revised manuscript.

9. Line 41 - Terminology Update: In line 41, "TMED10-gated" should be changed to "TMED10-mediated."

Response: We have replaced "TMED10-gated" with "TMED10-mediated" in the revised manuscript (lines 40–42).

10. Line 41 - MAPS System: The MAPS system of UcPS (PMID: 27295555) should also be mentioned for completeness.

Response: We have added a description of the MAPS system of UcPS in the revised

manuscript (lines 39–44).

11. Abbreviation for UcPS: Please update the abbreviation "UPS" to "UcPS" as it refers to unconventional protein secretion.

Response: We have unified the abbreviation to "UcPS" in the revised manuscript.

REVIEWER COMMENTS

Reviewer #4 (Remarks to the Author):

The manuscript has been significantly improved, and the authors have addressed the majority of my previous concerns. However, one issue remains that should be addressed before the manuscript can be accepted for publication.

In the TMED10 RNAi experiments, galectin-3 secretion is not reduced under basal conditions prior to LLOME treatment. This raises concerns that the knockdown efficiency may be insufficient to effectively impair the unconventional protein secretion (UcPS) function of TMED10, as multiple previous studies have demonstrated that TMED10 is required for galectin secretion under steady-state conditions. Therefore, the authors should validate their conclusions using TMED10 knockout cells together with TMED10 re-expression rescue experiments.

Alternatively, if the authors prefer to continue using RNAi, they should improve the knockdown efficiency and demonstrate the expected positive control phenotype — namely, that TMED10 depletion reduces galectin-3 secretion under steady-state conditions — before drawing conclusions regarding the effects of LLOME treatment.

Response: Thank you for your comments. Given the lengthy timeline associated with generating knockout clones, we have enhanced the knockdown efficiency of TMED10 in our model by adjusting transfection conditions. As shown in Figure S11A (n=3 biological replicates), TMED10 depletion reduces Gal-3 secretion under steady-state conditions, consistent with previous studies. Upon LLOMe-induced lysosomal damage, Gal-3 secretion levels show no significant change in TMED10-depleted cells (Figure S11A). These results are now included in the updated Figure S11A.

A

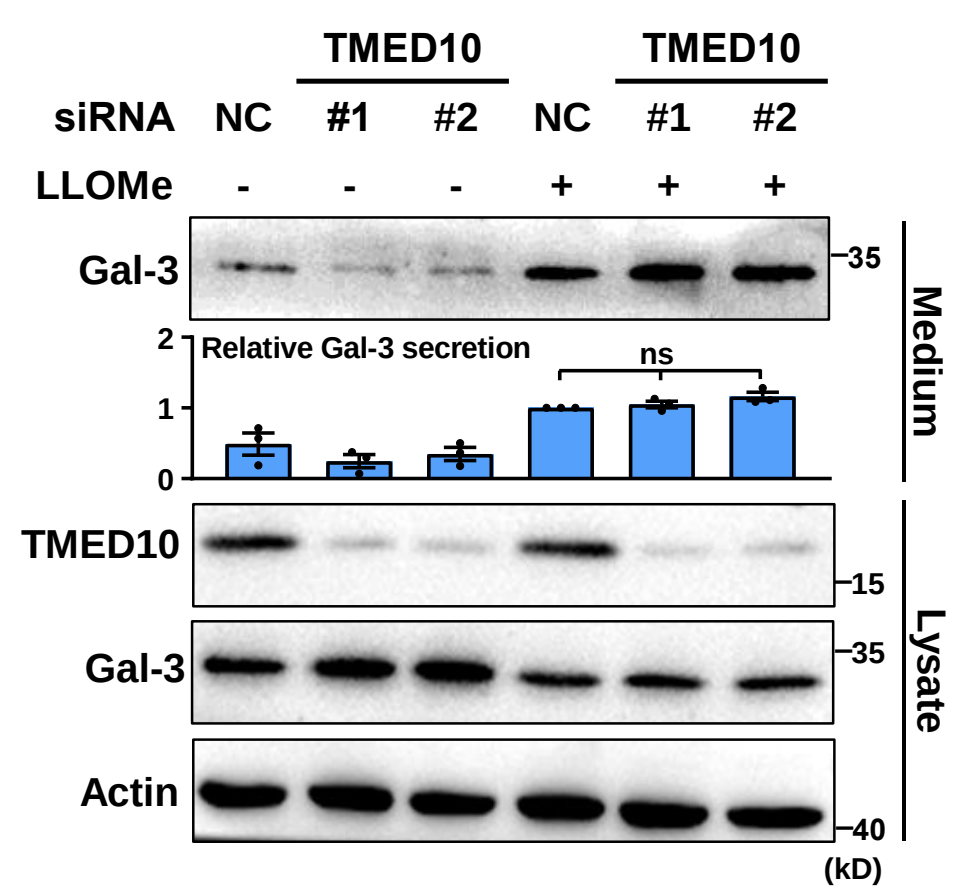

Figure S11A in the revised manuscript
